# Recent Advances in Limiting Fatigue Damage Accumulation Induced by Self-Heating in Polymer–Matrix Composites

**DOI:** 10.3390/polym14245384

**Published:** 2022-12-09

**Authors:** Jafar Amraei, Andrzej Katunin

**Affiliations:** Department of Fundamentals of Machinery Design, Faculty of Mechanical Engineering, Silesian University of Technology, Konarskiego 18A, 44-100 Gliwice, Poland

**Keywords:** self-heating effect, polymer–matrix composites (PMCs), viscoelasticity, structural degradation of PMCs, scale-based fatigue damage mechanism, cooling techniques, materials design, thermal conductivity

## Abstract

The self-heating effect can be considered as a catastrophic phenomenon that occurs in polymers and polymer–matrix composites (PMCs) subjected to fatigue loading or vibrations. This phenomenon appears in the form of temperature growth in such structures due to their relatively low thermal conductivities. The appearance of thermal stress resulting from temperature growth and the coefficient of thermal expansion (CTE) mismatch between fibers and neighboring polymer matrix initiates and/or accelerates structural degradation and consequently provokes sudden fatigue failure in the structures. Therefore, it is of primary significance for a number of practical applications to first characterize the degradation mechanism at the nano-, micro- and macroscales caused by the self-heating phenomenon and then minimize it through the implementation of numerous approaches. One viable solution is to cool the surfaces of considered structures using various cooling scenarios, such as environmental and operational factors, linked with convection, contributing to enhancing heat removal through convection. Furthermore, if materials are appropriately selected regarding their thermomechanical properties involving thermal conductivity, structural degradation may be prevented or at least minimized. This article presents a benchmarking survey of the conducted research studies associated with the fatigue performance of cyclically loaded PMC structures and an analysis of possible solutions to avoid structural degradation caused by the self-heating effect.

## 1. Introduction

The self-heating phenomenon appears in engineering structures made of polymers and polymer–matrix composites (PMCs) and usually has a negative influence on structural performance and residual life, especially when it dominates a fatigue process. To prevent the intensive degradation of such structures, it is essential to investigate the degradation mechanisms introduced by self-heating and its influence on the thermomechanical response of a structure. Based on this knowledge, it is possible to define preventing approaches for minimizing this degradation, which is of key importance in numerous applications, especially during intensive cyclic loading, e.g., nominal loading or fatigue testing of PMCs. In this section, the consideration of the self-heating effect as a catastrophic phenomenon in civil, mechanical and aerospace engineering applications is rationally justified. Furthermore, the primary motivations for conducting the current research are discussed. Many researchers have investigated this catastrophic phenomenon, which has been reviewed, e.g., in [1].

### 1.1. The Self-Heating Effect

**Figure 1:**
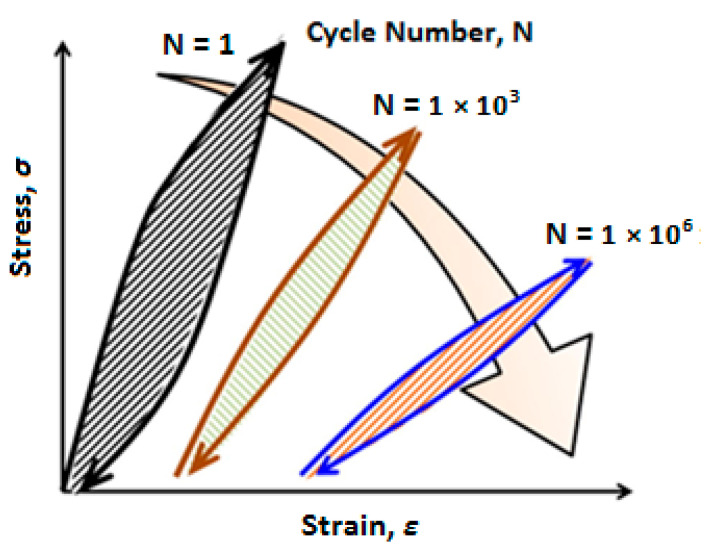
A schematic illustration of dissipated hysteresis thermal energy variations of a cyclically loaded PMC structure, adapted with permission from Ref. [5]. Copyright © 2017 Elsevier Ltd.

**Figure 2:**
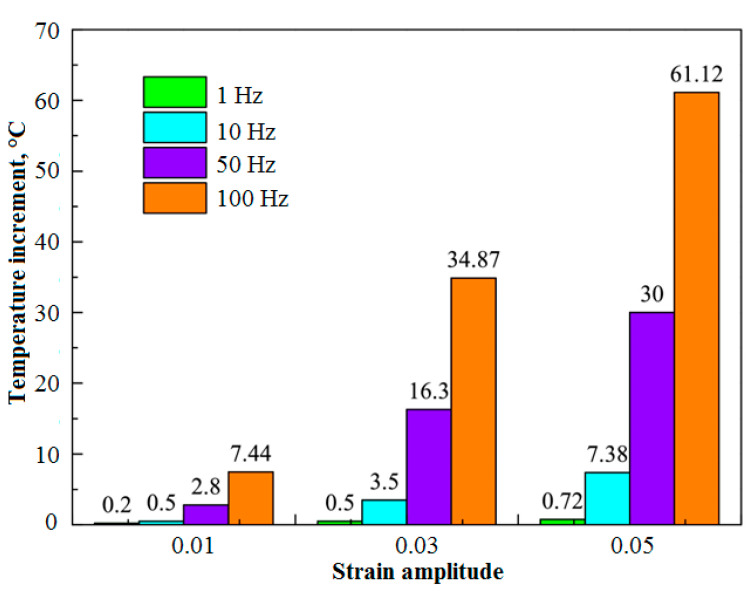
The influence of frequency and strain amplitude on the temperature of hydroxyl-terminated polybutadiene (HTPB) propellant under cyclic fatigue test, adapted with permission from Ref. [7]. Copyright © 2020 Elsevier Ltd.

**Figure 3:**
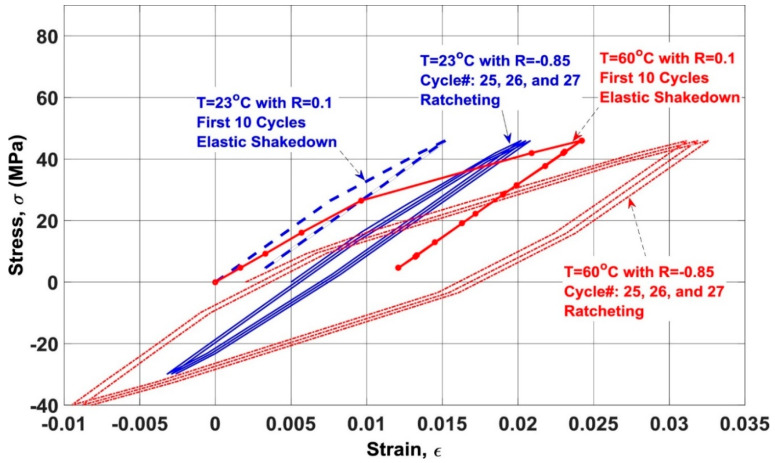
Hysteresis thermal energy loops for highly crystalline thermoplastic DuPont^TM^ Delrin^®^ 100 polymer under fatigue loading at two different temperatures, 23 °C and 60 °C, adapted with permission from Ref. [8]. Copyright © 2017 Elsevier Ltd.

The self-heating effect is an unfavorable phenomenon that occurs in both polymers and PMC structures subjected to vibrations or fatigue loading. The intensity of the self-heating phenomenon in such structures can be a function of material properties, geometry and loading conditions [2,3]. When a PMC specimen is subjected to a cyclic mechanical loading, such a composite due to its viscoelastic nature tends to dissipate a portion of mechanical energy in the form of thermal energy. The rest of the energy is dissipated due to elastoplastic behavior and fracture mechanisms, which occur from the very beginning of the operation. Due to the generally low thermal conductivity (*K*) of polymers, this thermal energy is stored in a structure. Since the stored thermal energy is accumulated, it provokes the heating of a structure and consequently the decrease in its mechanical performance causing irreversible changes in a material at the final stages of degradation [4]. The structural changes in a material influenced by the self-heating are caused by the increase in the activity of polymer chains with the increase in temperature, which, in consequence, results in material softening and, in ultimate cases, in the breaking of polymer chains. This can be considered a source of irreversible structural changes at the macroscopic level. This phenomenon is manifested by mechanical hysteresis when a structure is under cyclic loading. Figure 1 schematically illustrates how hysteresis loops or self-heating intensity of a cyclically loaded PMC can be influenced by changing the number of applied cycles [5]. As can be concluded, if we assume that the temperature of such a structure remains constant at the ambient temperature during its entire life, the total area of a loop, which represents dissipated energy, experiences a significant reduction as the number of applied cycles increases. In other words, it may be rational to expect that the area of a loop represents the dissipated mechanical energy in such a material for relatively low frequency and amplitude of loading to remain unchangeable as the number of cycles increases [6]. The primary reason behind this expectation may be explained by how the damage mechanisms in cyclically loaded PMC structures after some initial fatigue cycles depend on the material properties, load conditions and component geometry [5]. On the other hand, when the amplitude of cyclic loading or frequency is high, the area of dissipated energy will tremendously increase (see Figure 2) [7], as a direct consequence of which the magnitude of temperature will increase. This suggests the higher temperature that such a PMC experiences, the amount of thermal energy dissipated increases. Figure 3 depicts how the area of hysteresis loops for DuPont™ Delrin^®^ (Wilmington, DE, USA) acetal homopolymer resin, as an example, increases as the temperature increases [8].

The loading conditions have a significant influence on the development of the self-heating effect and the structural degradation as a consequence. In this case, one can observe the following relation: with the increase in the amplitude and/or frequency loading a structure is subjected to, the level of degradation caused by the self-heating effect occurring in a PMC structure will increase. It should be mentioned that the extracted thermal energy is irreversible according to the second law of thermodynamics (Clausius–Duhem inequality) [9], which justifies the danger behind the development of the self-heating phenomenon. From the microstructural point of view, the prime roots of the self-heating phenomenon in the polymer-based structures under high fatigue loading can be explained by the high amplitude and/or frequency of fatigue loading, which accelerates mechanical energy dissipation owing to the viscoelastic nature of polymers. This may consequently act as a catalyst in intensifying the structural degradation, provoking the premature destruction of such a structure [3,10].

### 1.2. Self-Heating Phenomenon as a Problem in Industrial Applications

**Figure 4:**
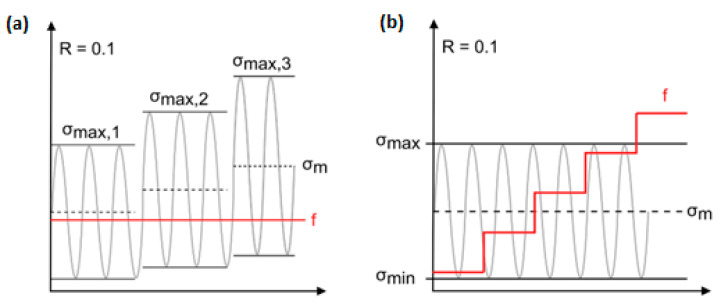
The schematic demonstration of (**a**) applying multiple-amplitude cyclic loads and (**b**) multiple-amplitude frequencies, adapted with permission from Ref. [11]. Copyright © 2020 Elsevier Ltd.

**Figure 5:**
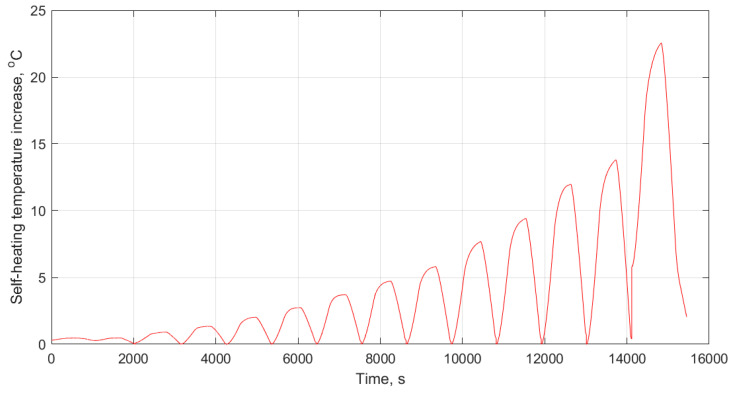
An exemplary illustration of self-generated heating temperature response for elevated loading.

In order to characterize the fatigue behavior of polymer-based structures dominated by the self-heating effect, a variety of stress amplitudes with constant frequency (see Figure 4a) or manifold frequencies with unchangeable stress during the fatigue test can be applied to such a structure, regardless of whether the test is carried out in low- or high-cycle fatigue regimes (see Figure 4b) [11]. An example of a thermal response of a structure under elevated stress levels is presented in Figure 5. Such a response is used in the determination of a fatigue limit in polymers and composites, which is broadly discussed further in Section 2.3.

According to [12], fatigue regimes can be generally classified as follows: very-low-cycle fatigue ($1\leq N_{VLCF}\leq 20$), low-cycle fatigue ($10^{2}\leq N_{VLCF}\leq 10^{5}$), high-cycle fatigue ($10^{6}\leq N_{HCF}\leq 10^{7}$) and very-high-cycle or gigacycle fatigue ($10^{8}\leq N_{VHCF}\leq 10^{11}$). Numerous differences occur for various fatigue regimes, both in loading type and mechanical response, which affect the development of the self-heating effect and subsequently the development of fracture mechanisms, which are analyzed below.

Generally, low-cycle fatigue (LCF) failure is described as a condition wherein a PMC specimen can tolerate a relatively low number of cycles until irrecoverable mechanical/thermal failure. The LCF regime is associated with the loading conditions where the applied stress in a PMC structure may be sufficient to provoke irrecoverable thermal or/and mechanical fatigue failure. To be more precise, LCF loads vary typically between 50% and 95% of the ultimate strength of such a composite, leading to high mechanical strains or thermal failure due to the occurrence of the self-heating effect, which may induce macrocracking and premature failure. In addition, LCF tests are typically conducted at a sufficiently low frequency (i.e., 10 Hz or below [13,14,15,16,17,18,19]); otherwise, premature thermal failure will occur before mechanical fatigue failure. From the other perspective, if such a PMC specimen is subjected to a relatively lower level of stress or strain loading, it may experience a higher number of fatigue cycles before its failure, as opposed to low-cycle fatigue failure wherein the specimen’s lifetime may be relatively short. The LCF failures in the presence of the self-heating effect have been reported in many engineering and military applications [20] involving turbojet engine elements subjected to short-time overloads [21] and solid rocket propulsion elements [22].

In advanced applications, such as cyclically loaded wind turbine blades (WTBs), carbon fiber-reinforced polymer (CFRP) and glass fiber-reinforced polymer (GFRP) composite sections are primarily designed and manufactured to experience high-cycle fatigue (HCF) and very-high-cycle fatigue (VHCF) regimes during their lifetime [23,24]. The primary causative factor contributing to this preference may be explained by how different the damage mechanisms in VHCF and LCF can be. The HCF regime is connected with the loading conditions where the subjected stress is below 30% of the ultimate strength of a composite material [25]; as a result of these conditions, the material remains within its elastic limit [26]. As opposed to LCF tests, wherein the loading frequency is typically lower than 10 Hz, HCF and VHCF tests are mainly conducted using accelerated fatigue testing techniques using comparatively high loading frequencies (i.e., ranging from approximately 10 Hz to 20.5 kHz [10,27,28,29,30,31]). Such testing modes are gaining a high level of interest, as they allow fatigue tests to be relatively affordable, and testing speed will be accelerated due to high loading frequency, which will result in a tremendous shortening of testing time. Implementing VHCF for up to 10^9^ cycles offers the opportunity to effectively characterize the fatigue damage mechanisms during the real lifetime, which may be unable to be determined by LCF testing. Nonetheless, fatigue testing of FRP composites under 10^9^ cycles with a conventional system at a frequency of 5 Hz will take just above 6 years [31]. In other words, it seems impractical, meaning that testing in the VHCF regime is extensively time-consuming and costly [32] unless the experiments are carried out at a higher level of frequencies so as to rationally reduce the time of fatigue experiments of PMCs [31]. From the other perspective, cyclic testing at high frequency (e.g., frequency in the ultrasonic range) will lead to a comparatively higher amount of dissipated energy, as a consequence of which a higher temperature is observed in a structure during testing, which is undesirable. This is especially important to be taken into consideration for many situations where the self-heating effect significantly influences the operation lifetime of a structure or element. The self-heating phenomenon has been reported in a variety of engineering applications including viscoelastic dampers [33,34], drive shafts [35,36,37], helicopter elements (e.g., helicopter tail rotor drivelines) [38,39,40,41], composite reservoirs for storing liquid fuels and methanol for the purpose of preventing self-ignition and explosion [42,43], composite sandwich panel structures in aviation industry [44], thick laminate sections (e.g., roots) of WTBs [45,46], and teeth of CFRP composite gears exposed to fatigue loading and rolling friction [47].

**Figure 6:**
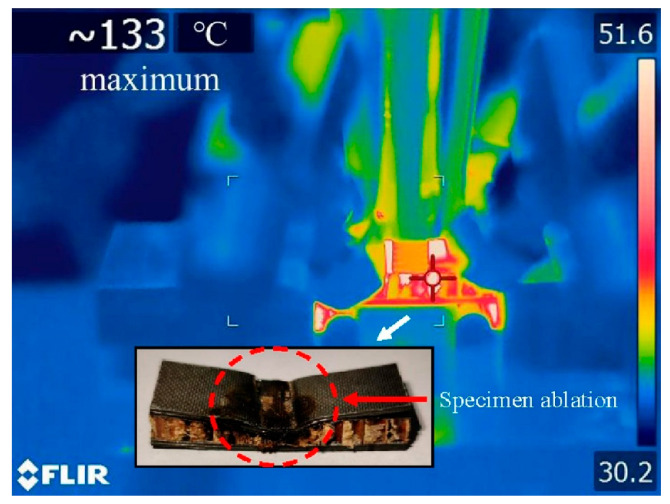
An exemplary thermal fatigue failure induced by self-heating effect in CFRP-aramid honeycomb sandwich structure under three-point cyclic bending in ultrasonic HCF regime, adapted with permission from Ref. [44]. Copyright © 2022 Elsevier Ltd.

Sandwich structures (e.g., CFRP-aramid honeycomb), which are often used in the aviation industry due to their superior flexural resistance to HCF and VHCF, may also experience accelerated degradation due to self-heating, e.g., when subjected to ultrasonic frequency loading; see, for instance, Figure 6 [44].

**Figure 7:**
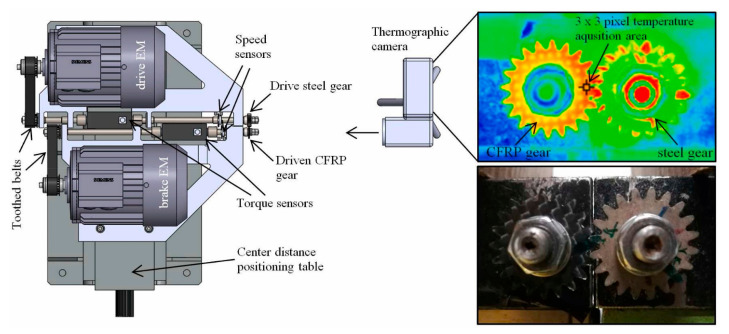
Model, illustration and thermogram illustrating the occurrence of the self-heating effect during the operation of a CFRP composite gear [47].

CFRP composite driveshafts are primarily implemented due to their outstanding specific stiffness and strength against torsion/bending loading. Nonetheless, the overall mechanical performance of such load-bearing driveshafts is highly temperature-dependent. This means that as the amount of heat generated on the surface of CFRP driveshafts increases, the level of strength degradation reached increases [47]. A thermogram that illustrates the occurrence of the self-heating effect in a CFRP-epoxy gear with a carbon volume fraction of 48% is presented in Figure 7.

**Figure 8:**
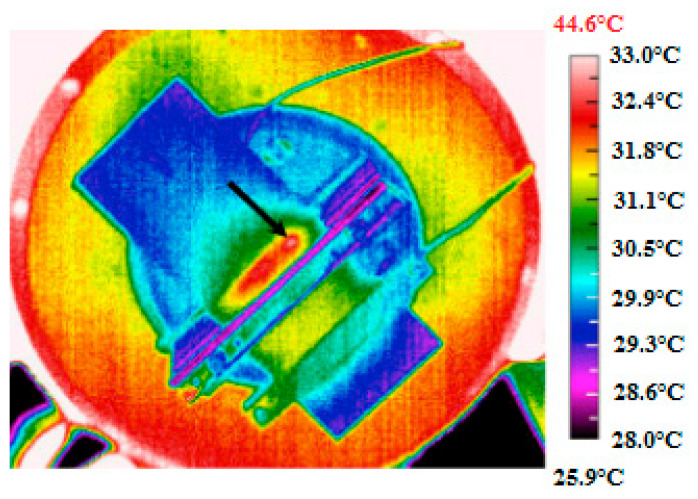
Thermal failure in a composite blade due to the self-heating effect shown by the arrow, adapted with permission from Ref. [28]. Copyright © 2016 Elsevier Ltd.

Another example presented in Figure 8 reveals the top surface of a composite blade under fatigue loading, wherein the thermal failure occurred because of the self-heating effect (e.g., the friction between delaminated plies) [28].

In conclusion, the self-heating effect can appear in all the above-mentioned fatigue regimes. However, depending on specific loading parameters, the evolution of the self-heating effect may significantly vary and accelerate the structural degradation and, in consequence, may lead to a sudden failure. Therefore, the characterization of the self-heating phenomenon in PMCs induced by cyclic fatigue (both stress- and strain-driven) loading is of utmost importance. In the following parts of this paper, the possibilities of limiting or preventing the self-heating effect during fatigue loading are discussed.

### 1.3. Motivation

The performed survey demonstrates that polymer and PMC structures subjected to fatigue loading may reach a critical condition in which the self-heating effect will dominate the fatigue process and significantly accelerate it and consequently lead to structural failure. In such a case, the main factor influencing fatigue acceleration is the self-heating temperature growth, which induces mechanical degradation, resulting in a special type of failure known as thermal failure [1]. This is especially important in the case of previously mentioned accelerated fatigue tests [29,31,48] when temperature growth has a rapid character due to generally high loading frequency. The occurrence of such problematic issues in the above-described engineering applications of PMC structures and, on the other hand, testing such structures under extreme loading conditions motivated the authors to carry out the current overview of the viable solutions for tackling the self-heating phenomenon with accompanying fracture mechanisms as well as analyzing reliable possibilities for reducing its influence on structures under fatigue loading. For this objective, the fatigue damage mechanisms with the presence of the self-heating effect at different scales are analyzed. The solutions are then introduced and categorized into two different aspects. Firstly, implementing different cooling scenarios may be the prime direction of studies for the purpose of effectively addressing the self-heating effect. Having provided an effective coolant system, such structures can even operate in extreme loading conditions; in particular, they were tested in a VHCF regime and resulted in a significant reduction in the time required for testing as well as expenditure. Secondly, optimal materials design and selection can be favorably considered as the viable solution to effectively avoid micro- and macroscopic structural degradation. This can be explained and achieved by the implementation of high thermally conductive and stable materials. The primary cores of the current study are focused on the comprehensive understanding of the scale-based fatigue damage mechanism and the possible viable solutions through cooling scenarios and optimal materials selection and design proposed by the authors to prevent or at least minimize the self-heating effect in such structures.

## 2. State-of-the-Art Review on the Self-Heating Effect

### 2.1. Phenomenological Analysis of the Self-Heating Effect

**Figure 9:**
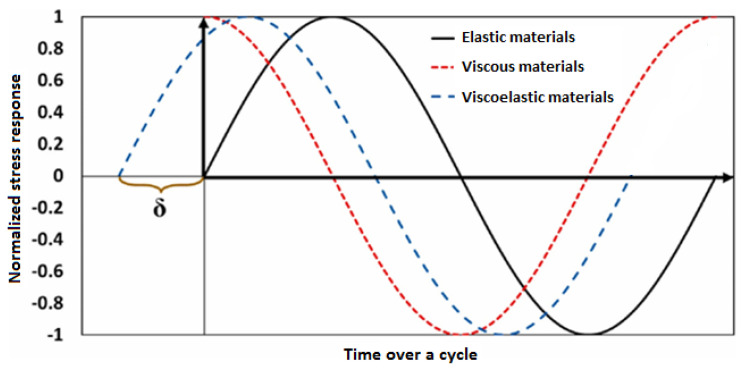
Schematic diagram of resulting stress under applied cyclic strain-driven loading for elastic, viscous and viscoelastic materials, adapted with permission from Ref. [49]. Copyright © 2021 Elsevier Ltd.

The generated thermal energy in polymeric and/or PMC structures due to their viscoelastic behavior caused by the self-heating phenomenon can be mathematically formulated regardless of whether the polymer matrix is thermoset or thermoplastic. For example, when a cyclic strain-driven loading is applied to a PMC specimen, the resultant stress would be similar, but with a phase lag (δ) (see Equation (1) and Figure 9) [49]. By δ, materials can be generally categorized into purely elastic $\left(\delta =0\right)$, purely viscous $\left(\delta =\frac{\pi }{2}\right)$ and viscoelastic $\left(0<\delta <\frac{\pi }{2}\right)$ (see Figure 9). The stress–strain relation for these classes of materials is as follows:

(1) $$\varepsilon =\varepsilon_{0}\sin\left(\omega t\right)\to \sigma =\sigma_{0}\sin\left(\omega t+\delta \right),$$

where ε and $\varepsilon_{0}$ denote the instantaneous strain and the amplitude of applied strain, respectively; σ and $\sigma_{0}$ indicate the instantaneous stress and stress amplitude of the loaded material, respectively; ω and t denote frequency and time, respectively. By implementation of the angle addition and subtraction theorem, the relation of stress can be expanded and then rewritten as follows:

(2) $$\begin{array}{l} \sigma =E^{*}\varepsilon_{0}=\sigma_{0}\sin\left(\omega t+\delta \right)\sin\left(\omega t\right)\to \;\\ \;\;\;\;\underset{Elastic\;\;\;Responce}{\underbrace{\sigma_{0}\cos\delta }}\sin\left(\omega t\right)+\underset{Viscous\;\;\;\;Responce}{\underbrace{\sigma_{0}\sin\delta }}\sin\left(\omega t+\frac{\pi }{2}\right), \end{array}$$

The mechanical response of viscoelastic materials can be alternatively represented by a complex modulus as follows:

(3) $$E^{*}=E^{\prime }+iE^{\prime \prime },$$

where $E^{*}$ is the complex modulus; $E^{\prime }$ is the storage modulus and $E^{\prime \prime }$ is the loss modulus which are proportional to elastic response and viscous response, respectively. From (2) and (3), the following relation is extracted for such a PMC material:

(4) $$\sigma_{0}\cos\delta =E^{\prime }\varepsilon_{0};\;\;\;\;\sigma_{0}\sin\delta =E^{\prime \prime }\varepsilon_{0}\to \;\delta =\tan^{-1}\frac{E^{\prime \prime }}{E^{\prime }},$$

It should be mentioned that the storage and loss moduli, which are the real and imaginary parts of the complex modulus, respectively, are dependent on numerous factors, particularly temperature, frequency and strain intensity [50,51,52,53,54].

Over the entire applied cyclic strain loading, the elastic and inelastic energy of polymeric materials can be extracted after integration and implementation (4) as follows:

(5) $$\begin{array}{l} \int_{0}^{\varepsilon_{0}}\sigma d\varepsilon =\int_{0}^{\pi /2\omega }\sigma \frac{d\varepsilon }{dt}\;\;=\;\;\omega \varepsilon_{0}\sigma_{0}\int_{0}^{\pi /2\omega }\left(\cos\omega t\sin\omega t\cos\delta +\sin^{2}\omega t\sin\delta \right)dt\\ \;\;\;\;\;\;\;\;\;\;\;\;\;\;\;\;\;=\;\underset{Elastic\;\;Dissipated\;Energy}{\underbrace{\varepsilon_{0}\sigma_{0}\frac{\cos\delta }{2}}}\;+\;\;\underset{Inelastic\;\;\;Dissipated\;\;Thermal\;\;Energy}{\underbrace{\varepsilon_{0}\sigma_{0}\frac{\pi \sin\delta }{4}}};\;\;\;\;0<\delta <\frac{\pi }{2}, \end{array}$$

**Figure 10:**
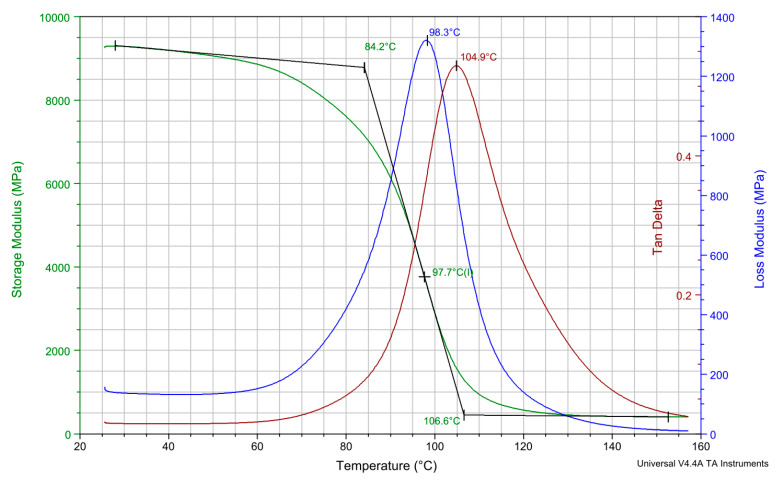
The exemplary DMA test results for the GFRP specimen.

The dynamic mechanical analysis (DMA) test is primarily used in order to determine the value of phase lag, δ. As the temperature increases, *δ* of a polymeric material exponentially increases to reach its glass transition (*Tg*) or melting (*Tm*) temperature, and then it exhibits a rubbery or glassy condition depending on the polymer behavior (i.e., storage and loss moduli) (see Figure 10 for example).

**Figure 11:**
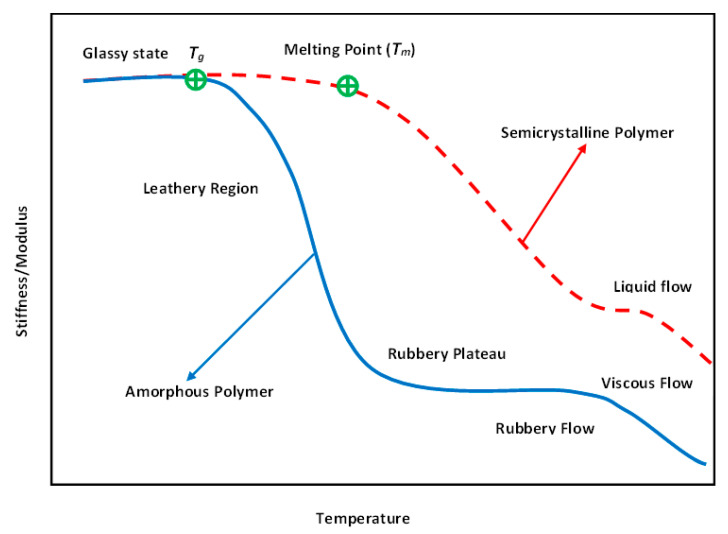
Melting and softening behavior of semi-crystalline and amorphous polymers.

From the microstructural point of view, polymers may reveal amorphous, crystalline or semi-crystalline behavior. The glass transition region mainly appears in an amorphous polymer exposed to heat wherein a viscous, liquid or rubbery state may occur (see Figure 11). This can be explained by how such polymers possess random molecular structures. As a result of an increase in temperature, such amorphous polymers soften gently (e.g., polymethyl methacrylate (PMMM)). Crystalline polymers have a highly ordered molecular morphology, as a result of which the softening may not occur as the temperature increases. The melting point ($T_{m}$) is mainly defined for crystalline polymers, which may be significantly higher than the glass transition temperature, i.e., $T_{m}>T_{g}$. Furthermore, for semi-crystalline polymers, both $T_{m}$ and $T_{g}$ are defined since they also have amorphous chains in their structures (e.g., polyetheretherketone (PEEK) and polyphenylene sulfide (PPS)). It is worth mentioning that the ratio of $T_{g}/T_{m}$ for semi-crystalline polymers varies from 0.5 up to 0.75 depending on the degree of crystallinity, so a polymer with a higher level of crystallinity reveals a lower value [55].

**Figure 12:**
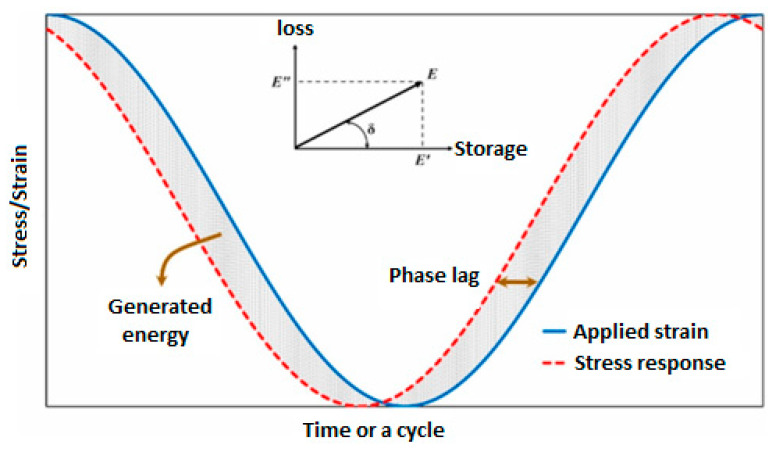
Schematic demonstration of the generated thermal energy for a polymer-based structure due to phase lag between stress and strain over an entire cycle, adapted with permission from Ref. [49]. Copyright © 2021 Elsevier Ltd.

**Figure 13:**
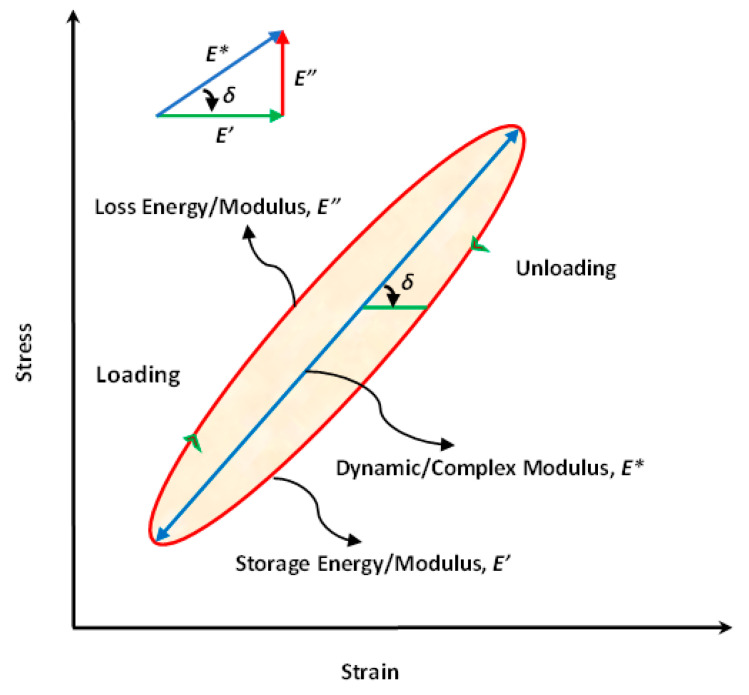
Schematic representation of the generated hysteresis loop and energy-based parameters.

Figure 12 schematically depicts the level of energy generation versus time for a polymer-based structure due to phase lag between stress and strain over an entire cycle [49]. This corresponds to a hysteresis resulting from the viscoelastic response of a structure, which is schematically presented in Figure 13 with an indication of energy-based parameters [11]. As can be vividly extracted from the diagram, the energy-based parameters can be categorized into storage and loss properties. The storage energy, which is proportional to storage modulus, can be explained when the cyclically loaded polymeric material is indeed unloaded, illustrated as the hatched area in Figure 13. From the other perspective, the elliptical area in Figure 13 (which has been obtained as the result of both loading and unloading over a cycle) is known as the loss energy, represented by a hysteresis loop, which is proportional to the loss modulus. To put it differently, if we connect the two vertices of the ellipse which form the major axis, the dynamic/complex modulus (overall viscoelastic performance) can be then defined and computed as the differences between the maximum and minimum response stresses divided by the differences between the maximum and minimum applied strains over a cycle. This parameter consists of both storage and loss moduli. Thus, the lower boundary of the hysteresis loop can locally illustrate the storage modulus during the relaxation time over an entire cycle, while the upper boundary of the hysteresis loop can locally demonstrate the level of loss modulus. It should be mentioned that since a PMC may experience more than 1 million cycles during its life, the estimation of the dissipated thermal energy over the entire life is of key importance.

From the thermodynamics point of view, by taking into account the self-heating effect in PMC structures, the generated energy ($q_{gen}$) over a fatigue cycle can be calculated by the summation of mechanical energy dissipation ($q_{dissip}$) and the rate of internal energy change per unit volume ($\Delta \dot{u}$) at loading frequency *f* as follows [56]:

(6) $$q_{gen}=q_{dissip}+\Delta \dot{u},$$

The amount of heat generation ($q_{gen}$) over a fatigue cycle per unit volume can be computed by multiplying the loading frequency by the total area of the hysteresis loop corresponding to such a frequency, as follows [56,57]:

(7) $$q_{gen}=f\left(\int_{0}^{2\pi /\omega }\sigma \frac{\partial \varepsilon }{\partial t}dt\right)=fA_{hystersis},$$

where f is the loading frequency applied to the specimen, ω is the angular frequency (i.e., ω=2πf), σ is the stress, ε is the strain, t is time and $A_{hystersis}$ is the area of hysteresis loop (stress–strain curve) over a cycle. Furthermore, the heat generation over a cycle per unit volume can also be determined using the loss modulus ($E^{\prime \prime }$) and the amplitude of applied strain ($\varepsilon_{0}$) in the following form [58,59]:

(8) $$q_{gen}=\pi f\varepsilon_{0}{}^{2}E^{\prime \prime },$$

The energy dissipated over a fatigue cycle per unit volume (*V*) can be determined by summing the heat transfer via conduction ($Q_{cond}$), convection ($Q_{conv}$) and radiation ($Q_{rad}$), as follows:

(9) $$q_{dissip}=\frac{1}{V}\left(Q_{cond}+Q_{conv}+Q_{rad}\right),$$

where $Q_{cond}$, $Q_{conv}$, $Q_{conv}$ and $\Delta \dot{u}$ are described in the following forms:

(10) $$Q_{cond}=-KA_{c}\;\nabla \;T,\;$$

(11) $$Q_{conv}=\bar{h}A_{l}\left(T-T_{\infty }\right)\;,\;$$

(12) $$Q_{rad}=\sigma eA_{l}\left(T^{4}-T_{\infty }^{4}\right)\;,\;$$

(13) $$\Delta u=\rho c_{p}\left(T-T_{\infty }\right)\to \Delta \dot{u}=\rho c_{p}\frac{\partial T}{\partial t},$$

where K is the thermal conductivity over the cross-section area of the specimen ($A_{c}$) and $\bar{h}$ denotes the average convective heat transfer coefficient over the lateral surfaces of the specimen ($A_{l}$); T and $T_{\infty }$ indicate the maximum surface temperature of such a composite specimen and the ambient temperature, respectively; σ and e denote the Stefan–Boltzmann constant ($5{.67\;\times \;10}^{-8}\;{W/m}^{2}K^{4}$) and emissivity, respectively; u is internal energy, ρ is the density, $c_{p}$ is the specific heat capacity of a material and t is time. Therefore, the temperature growth as the result of fatigue cycling can be determined as follows:

(14) $$\frac{\partial T}{\partial t}=\frac{\Delta \dot{u}}{\rho c_{p}}=\frac{q_{gen}-q_{dissp}}{\rho c_{p}}=\frac{fA_{hystersis}-q_{dissp}}{\rho c_{p}},$$

Noticeably, the heat flux via conduction during tension–tension fatigue loading can be negligible [60]. This can be explained by constant stresses across the cross-section of the composite specimen and the unchanged power of internal heat sources. In other words, the temperature gradient through the specimen thickness during tension fatigue loading is approximately zero. On the other hand, heat dissipation via radiation can be eliminated. Therefore, Equation (14) can be simplified for a composite specimen under merely tension–tension fatigue loading as follows:

(15) ∂T∂t=fAhystersis−qconvVρcp,

In order to logically formulate the caused self-heating effect under stationary or nonstationary conditions in a cyclically loaded polymeric structure [61], a couple of scenarios can be taken into consideration, which are broadly discussed in Section 4.

Generally, low-cycle fatigue failure, described as the state wherein a tested structure can tolerate a relatively low cycle until failure, occurs due to the application of high-frequency loading and/or a high amplitude of stress/strain during experiments. When the loading frequency is sufficiently low (e.g., below 5 Hz), low-cycle fatigue failure normally occurs as the result of applying a high level of cyclic stress/strain, which should be lower than the ultimate strength/strain. In such a case, the self-heating effect does not occur and fatigue has a purely mechanical character. By contrast, if such a structure is subjected to a relatively lower level of stress- or strain-driven loading, it may experience higher fatigue cycles before its failure, as opposed to low-cycle fatigue failure during which a structural lifetime may be relatively short. Since PMC structures are mostly designed and implemented for the long term, testing in HCF and VHCF regimes may be more favorable. The primary causative factor contributing to this preference may be explained by how different the damage mechanisms in VHCF and LCF can be. Characterization of failure mechanisms in the LCF regime may possess some complexities involving insufficient damage visibility. Testing specimens in the VHCF regime, however, may unfavorably be quite time-consuming and costly, unless the experiments are carried out at a higher frequency level, so as to rationally reduce the time of fatigue experiments of PMCs [31]. This loading frequency may vary from just below 1 Hz up to 20 kHz [16,29,62,63]. Lower frequency levels (normally less than 5 Hz) are primarily implemented for obtaining stress–life (S-N) curves to avoid the hysteresis effect [64], while the accelerated fatigue limit techniques using comparatively high loading frequency [58] as potential candidates have gained a high level of interest. Implementing the accelerated fatigue tests will result in comparatively high testing speed, low testing time [65] and consequently lower cost for testing.

### 2.2. Prevention of Self-Heating by Controlling Loading Parameters

The primary factors contributing to the lifetime of a polymeric component can be sought in (a) materials (e.g., type of polymer and reinforcement, volume fraction of fiber, stacking sequence of layers, hybridization of fibers and/or polymers, crosslinking of polymers), (b) processing parameters (e.g., void content, manufacturing defects) and (c) loading parameters (e.g., temperature, strain rate, frequency, stress ratio). Although all mentioned aspects take center stage simultaneously in the life assessment of such a composite under fatigue loading, the influence of loading parameters is deeply investigated in this section due to the scope of the current paper.

The classical approach to the evaluation of fatigue in structures is based on the determination of S-N curves, representing a decrease in residual stress as a function of a number of loading cycles. The S-N curve approach can be regarded as a cycle-dependent but time-independent technique for estimating the fatigue life of materials, including PMCs, which can be characterized according to ASTM and ISO standards. Such standards have been developed for cyclic axial loading (tension and compression) consisting of standards D3479 [66], D3039/D3039M-14 [13] and D3410 [67]. Similarly, the recommended ISO standards for fatigue testing include ISO 13003:2003 [68] and ISO EN 527-4:1997 [69]. Since the S-N method is conventional, it requires comparatively less advanced equipment for evaluating the fatigue performance of FRP composites. Considering that S-N data are mostly determined for low frequency loading (mainly 5 Hz or below), characterizing the fatigue behavior of PMC structures using this technique would be a time-consuming process.

*For many decades, the following question has been asked: How are the data obtained during fatigue testing influenced by loading frequency?*

Although countless studies have been carried out in order to explore a trend to categorize and standardize the dependency of fatigue lifetime of a component on frequency, no reliable approach has been reported for this objective yet. Interestingly, the vast majority of studies advocate that at a low frequency regime, the fatigue performance of polymeric components is approximately frequency-independent. Some researchers have reported that roughly 5 Hz is the upper limit of frequency in which fatigue tests can be carried out without dependency on frequency [64]. For this reason, Zhou and Mallick [70,71] investigated the influence of load frequency on the fatigue behavior of 40 wt.% talc-filled polypropylene (PP) and 33 wt.% short glass fiber-reinforced polyamide-6.6 (SGFR-PA6.6) with stress ratio *R* of 0.1. They showed that increasing frequency up to 2 Hz will directly lead to an improvement in the fatigue performance of the talc-filled PP composite at maximum stress levels of 80% and 85% of ultimate strength at the ambient temperature. They also revealed an opposite trend for the fatigue life of such a composite when the frequency is in the range of 2 < *f* < 5 Hz, remaining unchanged for the rest of frequency ranges (up to 20 Hz). Tao et al., however, investigated the effect of frequency and stress ratio *R* on tensile fatigue of carbon cord-reinforced hydrogenated nitrile butadiene rubber (CC-HNBR) composites [72]. They tested the composite specimens with different frequencies ranging up to 20 Hz. According to their results, this frequency regime had an unimportant influence on the fatigue performance of such polymeric specimens because of the generation of an insignificant amount of thermal heat during the entire fatigue test [72]. However, many studies reported the upper limit of frequency at which the mechanical properties will be unimportantly affected is 5 Hz. This means that if the loading frequency is increased (normally more than 5 Hz), this may intensively increase the total area of hysteresis loops generated in this high frequency regime, as a result of which the temperature on the surface of such a structure may increase sharply [72]. This may provoke thermal failure or even jeopardize the structural integrity of a cyclically loaded composite if a temperature overtakes $T_{g}$ [3,49] or $T_{m}$ of polymers. However, the results of previous research studies by the authors [1] clearly showed that a critical self-heating temperature was understood as a temperature at which a significant intensification of degradation mechanisms occurs and also leads to a thermal failure. As reported previously, this temperature is usually much lower than *T_g_*. The concept of critical self-heating temperature is discussed in detail in Section 3.2.

**Figure 14:**
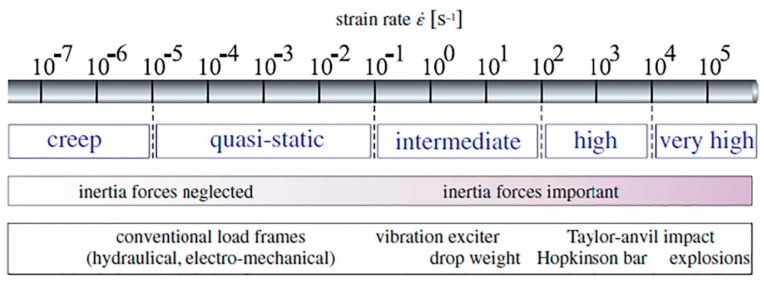
Classifying the strain rate regimes, adapted with permission from Ref. [73]. Copyright © 2021 Elsevier Ltd.

It can be concluded that the hysteretic effects can be negligible when such a composite is implemented at a very low frequency regime. In other words, during testing, the total area of hysteresis loops of such a composite caused by a very low loading frequency would be comparatively insignificant. In order to obtain the S-N curve for such a PMC specimen, the frequency of fatigue loading is assumed to be roughly 5 Hz or even less because of preventing the unfavorable influence caused by the self-heating phenomenon. The intensity of loading using the concept of strain rate ($\dot{\varepsilon }$), regardless of the types of polymers and fibers, as one of the fatigue loading parameters can be classified into different types. As can be seen in Figure 14 based on recommendations [13,66,67,68,69] (ASTM and ISO standards mentioned earlier), static tests of such composites can be carried out in a quasi-static regime, while cyclic tests, involving fatigue tests, are mainly performed in an intermediate regime [73]. However, as the strain rate (or strain intensity) applied to a structure during cyclic testing increases, the amount of mechanical energy dissipated in the form of thermal energy increases, resulting in an increase in surface specimen temperature. This elevated temperature primarily acts as a catalyst in the degradation of mechanical properties (i.e., strength, storage modulus and residual life) of such a structure. This leads to the following conclusion: as the temperature that a PMC experiences increases, the level of structural degradation or stiffness degradation that takes place increases regardless of whether that temperature has been applied externally [74] or internally caused by the self-heating effect.

**Figure 15:**
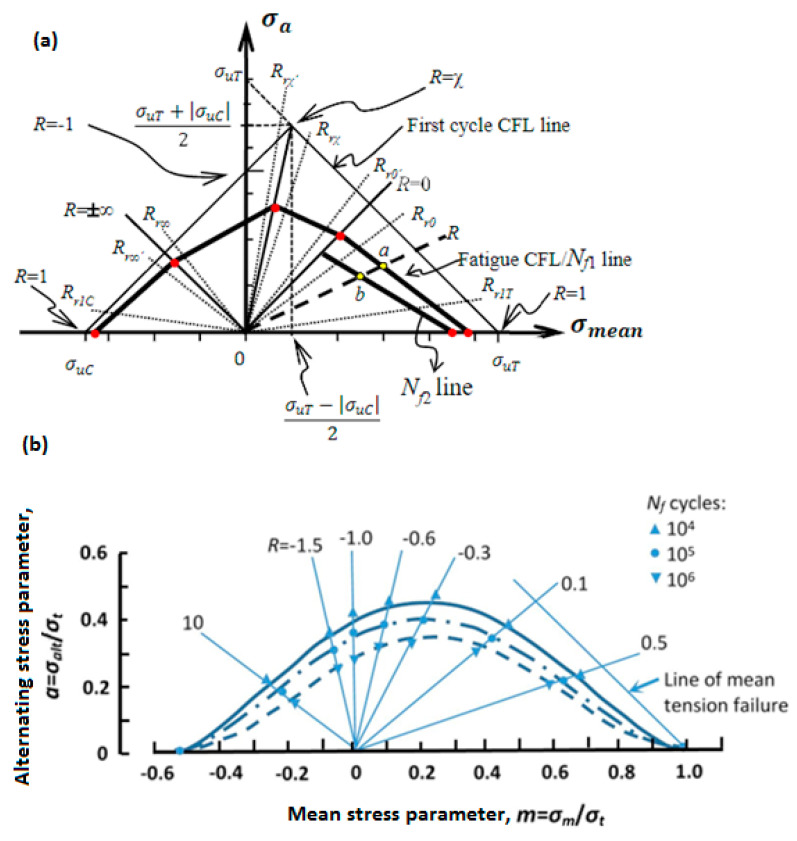
(**a**) A schematical illustration of cyclic loading ratios (adapted with permission from Ref. [75]. Copyright © 2019 Elsevier Ltd.) and (**b**) an exemplary illustration of fatigue life based on the Goodman diagram for carbon/Kevlar-49/epoxy laminate (adapted with permission from Ref. [64]. Copyright © 2020 Elsevier Ltd.).

From the point of view of the fatigue loading ratio, Figure 15a schematically illustrates the cyclic loading ratios [75], wherein R denotes the fatigue stress/strain ratio $\left(\begin{array}{l} R=\sigma_{\min}/\sigma_{\max} \end{array}\right)$, and $\sigma_{uT}$ and $\sigma_{uC}$ indicate the ultimate tensile and compression strengths of the specimen, respectively. It should be mentioned that $\sigma_{\max}$ indicates either $\sigma_{uT}$ or $\sigma_{uC}$, depending on the type of loading. The stress ratio $\left(\begin{array}{l} R=\sigma_{\min}/\sigma_{\max} \end{array}\right)$ takes a center stage in obtaining the S-N curve during fatigue testing. The fatigue loads can be classified by the stress ratio parameter. Noticeably, (+) and (−) are algebraic representations for tension and compression loading, respectively. R=1 demonstrates the static loading, while R=−1 is an indication for symmetric (known also as alternating fully reversed) strain/stress loading, which may be taken into consideration as the most catastrophic type of fatigue loading. In other words, when R=−1, the fatigue life of the PMC specimen would be at the minimum level compared with other forms of loading. R=0 depicts the repeated (zero-tension) cyclic loading, while R=−∞ reveals repeated (zero-compression) stress/strain loading, which may be described as the same as R=0. In addition,0<R<1 demonstrates tension–tension cyclic loading (the most common type of fatigue testing), while 1<R<∞ shows compression–compression cyclic loading. However, −∞<R<0 indicates compression–tension loading, which has not yet been standardized due to its complexity. Figure 15b demonstrates the fatigue life for hybrid carbon/Kevlar-49/epoxy laminate with various mean stress ($\sigma_{m}$) effects with constant life (Goodman) diagrams shifting from left to right [64,76]. The main causative factor contributing to this left-to-right shifting in Goodman diagrams can be rooted in how PMC structures normally reveal a greater magnitude of strength during cyclic tension loading, as opposed to compression fatigue loading [64].

**Figure 16:**
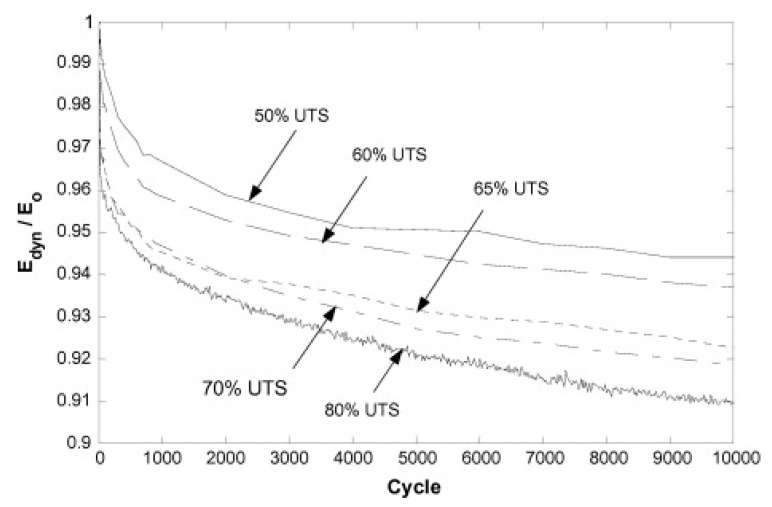
An exemplary demonstration of normalized stiffness reduction under stress ratios of one-half or above (0.5 ≤ *R* ≤ 0.8), adapted with permission from Ref. [74]. Copyright © 2013 Elsevier Ltd.

**Figure 17:**
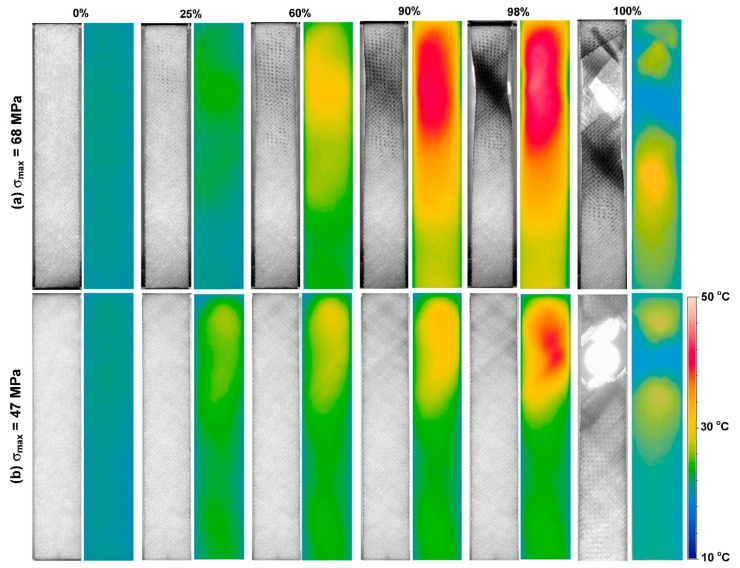
An experimental demonstration recorded using an infrared thermal camera for the self-heating effect at various percentages of fatigue life of ${\left(\theta =\pm \;45\right)}_{2s}$ GFR/epoxy composite, adapted with permission from Ref. [77]. Copyright © 2018 Elsevier Ltd.

Figure 16 vividly depicts how the stiffness of a specific FRP laminated composite is undesirably degraded as the cyclic load ratio increases [74]. Movahedi-Rad et al. [77] investigated how the energy dissipation of angle-ply glass/epoxy composite laminates with stacking sequence of ${\left(\pm 45\right)}_{2s}$ was influenced by different stress levels with an unchanged ratio of 0.1 so as to cover many fatigue lives, ranging from 500 cycles up to 10^6^ cycles. They showed that as the number of cycles and/or stress ratio increased, a relatively larger amount of mechanical energy was dissipated, and consequently a greater amount of thermal energy was stored, which was explained by much more friction in the area of unbounded zones as a consequence of damage growth. As shown in Figure 17, the specimens experienced the maximum temperature values of 30 °C and 45 °C when they were subjected to low/intermediate stress levels and higher stress levels, respectively. 

### 2.3. Fatigue Limit and Role of Self-Heating

**Figure 18:**
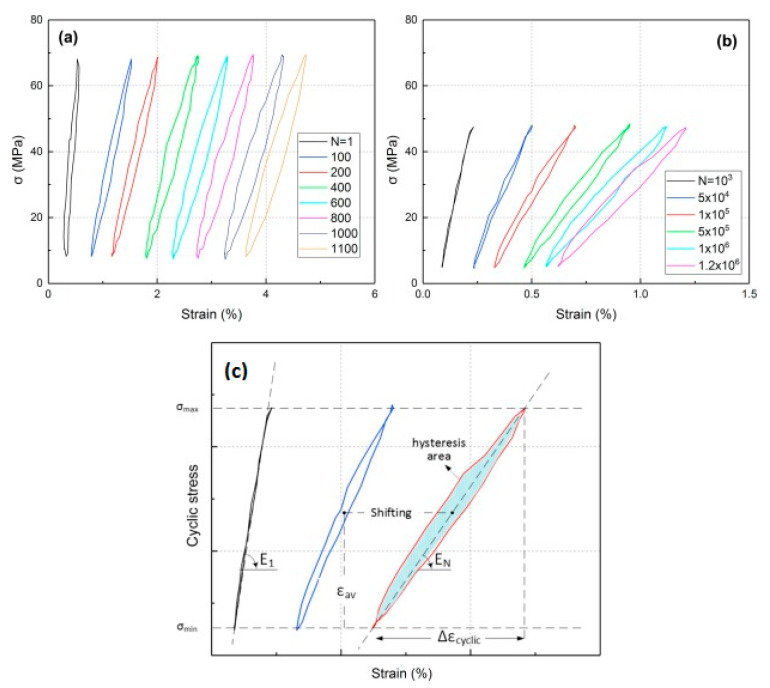
Hysteresis area alteration (**a**) applied to $\sigma_{\max}=68\;\mathrm{MPa}$ and (**b**) $\sigma_{\max}=47\;\mathrm{MPa}$ and (**c**) slope variation as the result of an increase in the number of cycles, adapted with permission from Ref. [77]. Copyright © 2018 Elsevier Ltd.

**Figure 19:**
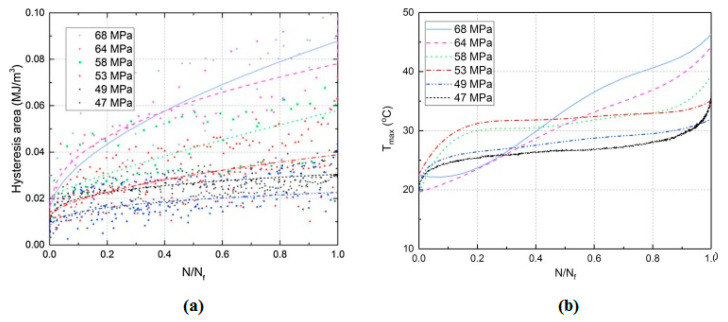
(**a**) Alteration of hysteresis area and (**b**) variation of maximum self-heating temperature as the function of applied stress level and cycle ratio (*N*/*N_f_*), adapted with permission from Ref. [77]. Copyright © 2018 Elsevier Ltd.

The FRP laminated specimens at different stress regimes should be tested before being implemented in potential engineering applications to obtain an acceptable safety factor. Since the FRP composites due to their extraordinary mechanical properties are normally subjected to HCF and VHCF regimes, implementing the conventional S-N curve method technique at very low frequency may be extensively time-consuming and consequently expensive. The number of cycles is the key factor when it comes to fatigue life assessment so that the residual life of such a polymeric structure can be computed. Figure 18 reveals how the hysteresis effect is influenced by variation of the applied stress and the number of fatigue cycles, which can be indicated by the slope of the major axis of elliptical-like hysteresis loops [77]. As can be concluded, an increase in the number of cycles resulted in a decrease in the slope of the major axis of the elliptical hysteresis loop, which is directly connected with the stiffness of the specimen. This means that the residual stiffness of a PMC specimen decreased as the number of cycles increased, which is a result of the viscoelastic nature of a polymeric matrix of a PMC. More importantly, it had been demonstrated that as the stress and number of cycles experienced by the typical PMC structure increased, the amount of thermal energy that could be generated increased (see Figure 19a). The same trend was reported for the self-heating temperature. As can be seen, the effect of self-heating had been accelerated as the number of fatigue cycles and the stress levels increased (see Figure 19b).

To effectively address this problematic issue and accelerate the speed of cyclic loading tests, researchers have considered the development of rapid fatigue limit techniques for the characterization of the fatigue damage evolution, which is considered an irreversible process through which energy is dissipated. For this objective, many intrinsic thermodynamic-based techniques (e.g., purely experimental or combined theoretical/numerical/experimental methodologies) have been developed and implemented involving acoustic emission (AE), digital image correlation (DIC), X-ray industrial computed tomography (ICT), infrared thermography (IR), self-heating-based thermography (SHT) and surface crack density, which have been reviewed in [1,78,79,80]. Among them, the SHT methods have been recently gaining great attention due to their comparative simplicity and acceptable accuracy. The SHT methods were first proposed for metals; however, they were then developed for PMCs.

Unlike conventional S-N curves for FRP composites which are both time-demanding and costly due to requiring numerous specimens for testing at various levels of *R* [81,82], the developed fatigue limit methods require much less time for testing compared to the conventional S-N curve method [29,31]. This can be explained by how the fatigue limit of such a composite can be determined using merely a single specimen, as opposed to constructing an S-N curve for which numerous specimens should be tested, and the approximation technique should then be implemented.

The fatigue limit in a PMC structure can be described as the highest level of stress at which the temperature gradient on the specimen surface is approximately zero. As a direct consequence of implementing energy-based fatigue limit techniques, temperature–cycle (T–N) and temperature–strength (T−σ) curves can be normally achieved.

**Figure 20:**
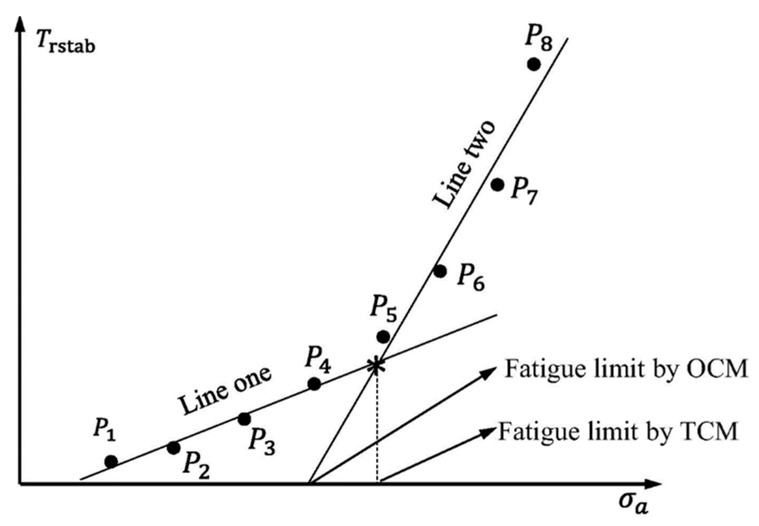
A schematic illustration of fatigue limit prediction using energy-based Risitano’s and Luong’s models, adapted with permission from Ref. [81]. Copyright © 2017 Elsevier Ltd.

**Figure 21:**
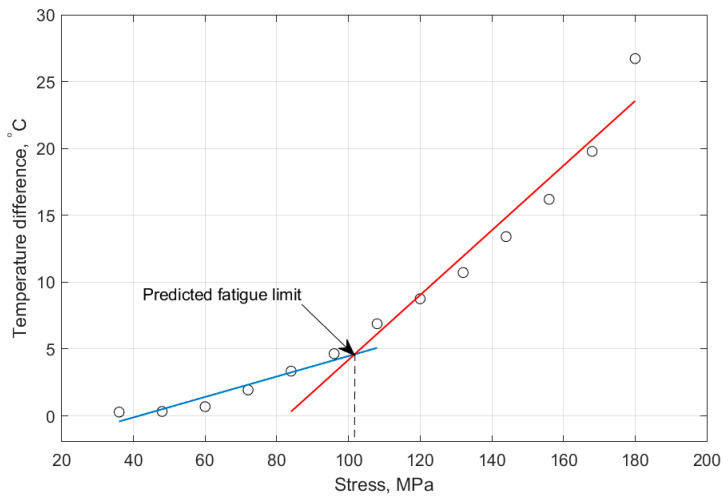
An exemplary fatigue limit determination of CFRP composite.

Risitano’s model [83,84] and Luong’s approach [85,86] have been well known as thermodynamic-based techniques for characterizing the fatigue limit during cyclic loading based on a temperature–strength (T−σ) curve. Figure 20 schematically shows how the fatigue limit can be characterized by implementing the energy-based Risitano’s and Luong’s models, which are known as the one curve model (OCM) and two curve model (TCM), respectively [81]. According to Risitano’s model, the fatigue limit can be calculated with the aim of a crossover between the horizontal axis and the second or enhanced temperature line induced by dominated thermoplastic (friction and microstructural evolution) under comparatively high stress loading amplitudes [83,84]. This approach may be quite conservative. On the other hand, two lines are implemented in Luong’s model, which is comparatively less conservative [85,86]. In addition to OCM and TCM, the temperature integral technique has received great interest from researchers. Unlike OCM and TCM which are completely intrinsic, the temperature integral technique implements the internal FRP composite thermal conduction properties and the external heat transfer (mainly in the form of convection) with the surrounding environment [87,88]. For this purpose, Huang et al. [89] determined the fatigue limit of angle-ply CFRP laminates with the stacking sequence of [±45°]_8_ subjected to cyclic shear loading with the presence of a self-heating phenomenon. The exemplary results for the determination of the fatigue limit are presented in Figure 21. This figure reveals the relationship between maximum loading amplitude and stabilized temperature rising. As can be seen from the provided graph, the heat generation rate increased gradually at comparatively lower stress values and increased significantly at greater stress levels. The maximum changing point between those two different stabilized temperature increases was assumed as the fatigue limit, which was just above 100 MPa.

**Figure 22:**
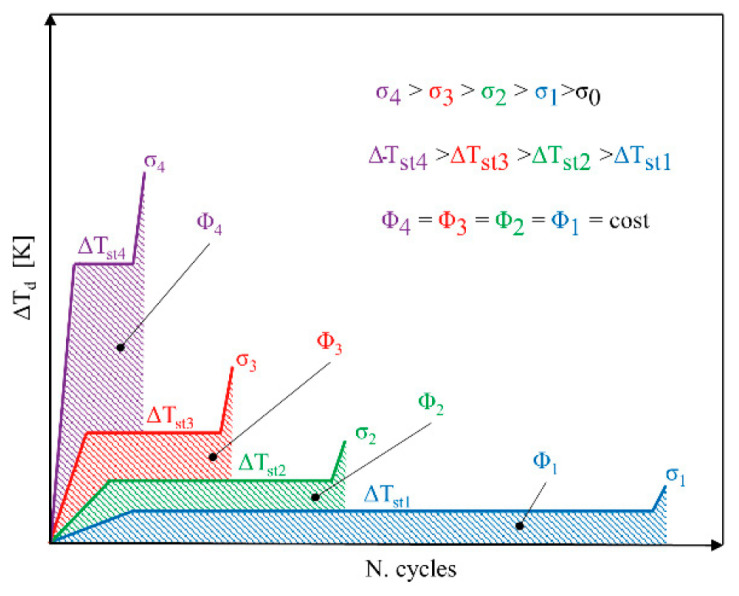
A schematic representation of temperature–cycle (T−N) curves under different applied stress levels, adapted with permission from Ref. [90]. Copyright © 2020 Elsevier Ltd.

In addition to the T−σ curve, the temperature–cycle (T−N) curves can be used for the sake of characterizing the fatigue behavior of composites. Figure 22 illustrates a schematic representation of temperature–cycle (T−N) curves for different applied stress levels [90]. In order to extract T−N curves, the assumption of three different phases existing is implemented (see Figure 22). Firstly, a rapid temperature change on the specimen surface occurs, whose rate is dependent on the applied stress level. After the initial increment, the temperature value will approximately remain unchanged for a comparatively high number of fatigue cycles, called temperature stabilization ($\Delta T_{st}$) or stationary condition. In the final phase, the temperature significantly increases until mechanical/thermal fatigue failure occurs, which is called a nonstationary situation. Additionally, as the level of stress amplitude applied to a tested structure increases, there will be an increase in the value stable temperature appearing on the specimen surface, which can be described by the energy parameter (*Φ*). It is assumed to be independent of applied stress, the stress ratio (*R*) and the loading frequency (*f*). By knowing *Φ* and the stabilized temperature associated with a subjected stress level, it is possible to determine the number of cycles at which the specimen fails (i.e., *N* = *Φ/*ΔT).

**Figure 23:**
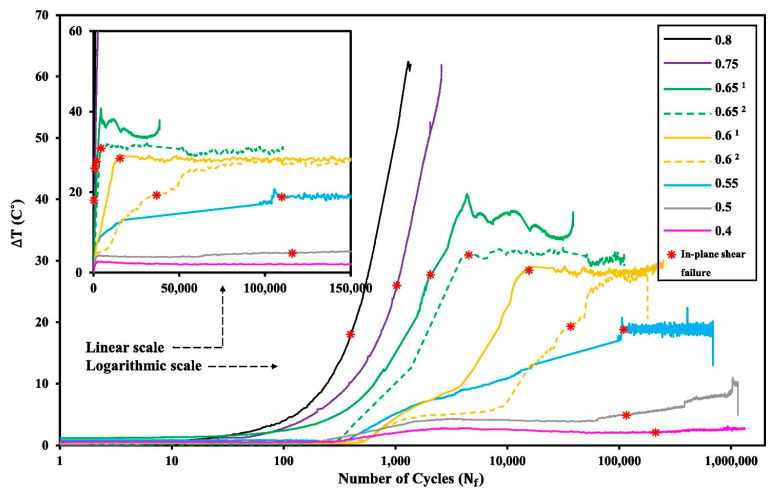
An exemplary plot of temperature evolution vs. stress alteration for the angle-ply [±45]_2s_ CFRP composite, adapted with permission from Ref. [91]. Copyright © 2022 Elsevier Ltd.

For this purpose, Mandegarian et al. [91] investigated the in-plane shear fatigue behavior of ${\left[\pm 45\right]}_{2s}$ angle-ply CFRP composite under fully reversed loading (R=−1). A number of staircase-like stress amplitudes were applied to such a composite specimen so as to obtain the same number of cycles wherein the fatigue failure occurs in such a laminated CFRP composite. Figure 23 illustrates how the number of cycles until failure can vary as a function of applied stress levels (e.g., ranging from *R* of 0.4 to 0.8) [91].

From the other perspective, a PMC structure, depending on the type of applied mechanical fatigue loading (i.e., bending, tension, shear tension–compression or multiaxial loading) and the magnitude of applied loading (i.e., frequency and stress/strain amplitude), may indeed experience both mechanical and thermal loading, as the result of which various fracture mechanisms may occur [92,93,94], which are deeply discussed in Section 3. Due to the entropy generation in the irreversible thermodynamic process caused by the self-heating effect [19,54,95], a structure may face mechanical failure or thermal failure. According to this, a mechanical fracture may occur when the influence of mechanical loading is comparatively more dominant than thermal loading, while thermal failure will occur if the self-heating effect is more dominant [64,89]. Thus, mechanical and thermal failures are associated with stationary and nonstationary scenarios, respectively.

**Figure 24:**
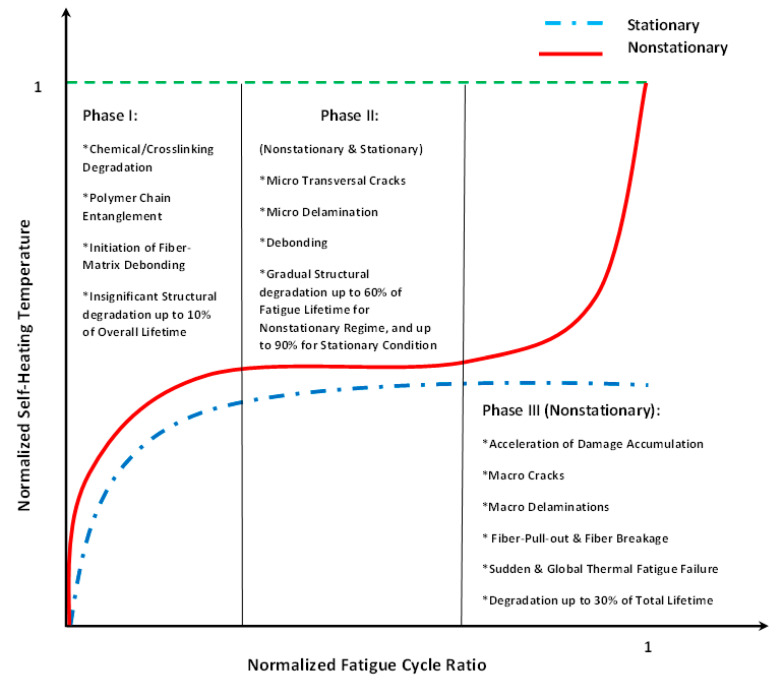
A schematic process of fatigue damage propagation in both stationary and nonstationary self-heating regimes.

As can be extracted from both stationary and nonstationary self-heating conditions illustrated in Figure 24 [87], the incremental temperature gradients begin steeply and decline slightly as the number of cycles increases in the first phase for both scenarios, which can be characterized and quantified by the implementation of equilibrium thermodynamics. During this stage, crosslinking degradation of polymer chains occurs and fiber–matrix debonding begins. During the next stage of the stationary self-heating scenario, a steady-state evolution called the stabilized self-heating temperature takes place over a prolonged period of time or a multitude of fatigue cycles. This trend can be explained by a thermal equilibrium point between the generated thermal energy on the polymer-based specimen surface and the heat removal through convective and conductive heat transfers as well as radiation into the surrounding environment [87]. Notably, the magnitude of heat transfer via radiation is comparatively lower than other forms, which can be negligible [87].

On the other hand, during the second phase of the nonstationary self-heating regime, the temperature profile experiences an upward trend linearly as the number of fatigue cycles increases (see Figure 24). The primary causative factors contributing to this trend can be sought in the synergy between mechanical fatigue and entropy generation (irreversible morphological and chemical variations [3]) because of the viscoelastic nature of polymers. Therefore, due to the high level of entropy generation caused by accumulated microcracking and delamination [3], structural performance may begin to remarkably degrade, which will be more catastrophic as the applied frequency and stress/strain amplitude of fatigue increase. The third phase of the nonstationary self-heating scenario begins when the macrocracks and macrodelaminations appear initially and their numbers increase with the increase in cycle number. The main causative parameters contributing to the continuous macrocrack formation process in FRP composites can be rooted in the high frequency and stress/strain ratio during testing, which indeed act as catalysts for propagating and interacting among the transverse and interlaminar microcracks and delamination in the vicinity of macrocracks, leading to releasing a large amount of energy in the structure. The released heat energy will undesirably provoke mechanical softening among polymer chains and consequently result in a sharp exponential temperature growth on the PMC surface, leading to structural degradation and sudden thermal failure.

**Figure 25:**
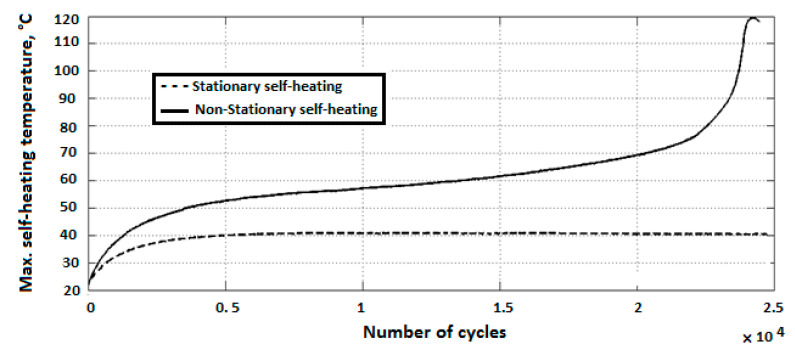
Exemplary self-heating temperature history curves for stationary and nonstationary self-heating scenarios, adapted with permission from Ref. [97]. Copyright © 2017 Elsevier Ltd.

**Figure 26:**
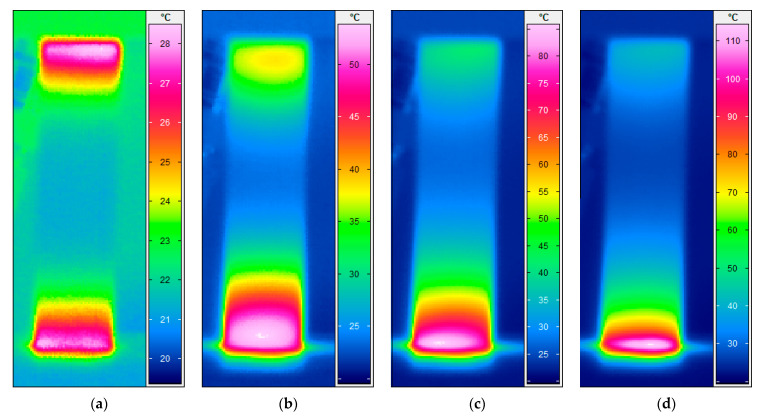
Infrared images of the cyclically loaded GFRP specimen with self-heating effect at (**a**) the beginning of loading (phase I), (**b**) near the end of phase II, (**c**) in the middle of phase III and (**d**) at failure.

Unlike the stationary condition induced by mechanical fatigue loading wherein a gradual linear temperature growth occurs [61], the nonstationary scenario is a complex phenomenon wherein a large amount of heat generation appears in the form of internal energy (see Equations (6) and (13), extracted from the first principle of conservation of energy thermodynamics [96]), which is highly dependent on environmental factors (e.g., ambient temperature, convective heat transfer coefficient), material properties (e.g., thermal conductivity), geometry (particularly thickness) and loading parameters (e.g., the magnitude of applied frequency and stress/strain ratio). This can be additionally explained by entropy inequality (i.e., second law of thermodynamics) [5]. In a stationary self-heating scenario, two different phases may occur, while three separate stages will take place during a nonstationary scenario in a cyclically loaded polymeric structure [3]. These two or three different phases can be extracted from the beginning of cyclic fatigue testing until the final breakage/failure in a loaded structure by using some devices, such as an infrared (IR) thermographic camera [97] (see Figure 24). Figure 25 illustrates the exemplary measured temperature responses during stationary and nonstationary self-heating regimes [97]; in addition, an exemplary IR image of the polymeric specimen surface temperature in different phases in the presence of the self-heating effect is shown in Figure 26.

**Figure 27:**
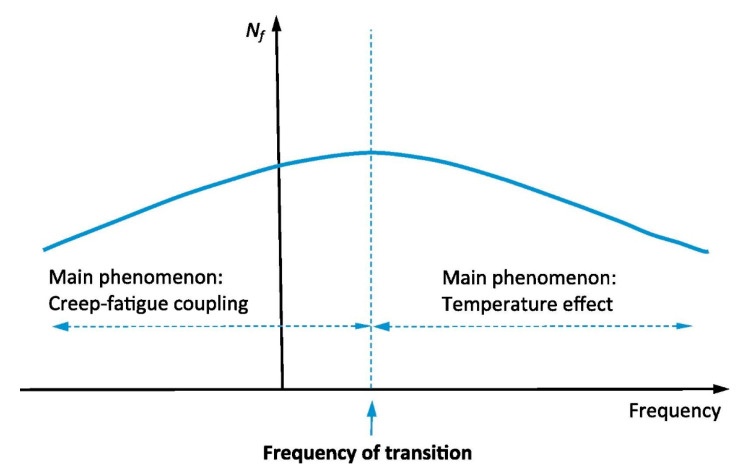
The schematic representation of the frequency transition effect on of critical self-heating temperature induced by failure modes, adapted with permission from Ref. [64]. Copyright © 2020 Elsevier Ltd.

A scheme of failure modes resulting from frequency alteration is presented in Figure 27 [64]. For example, regardless of the effect of the load ratio, the critical condition occurs at the frequency transition. This means that if the applied frequency is greater than the frequency transition, the thermal fatigue failure mode would be dominant in such a structure. Otherwise, at a constant level of stress when the frequency is sufficiently low, the decrease in fatigue life is due to the creep influences.

## 3. Fatigue Behavior of Polymers and PMCs Dominated by Self-Heating Effect

**Figure 28:**
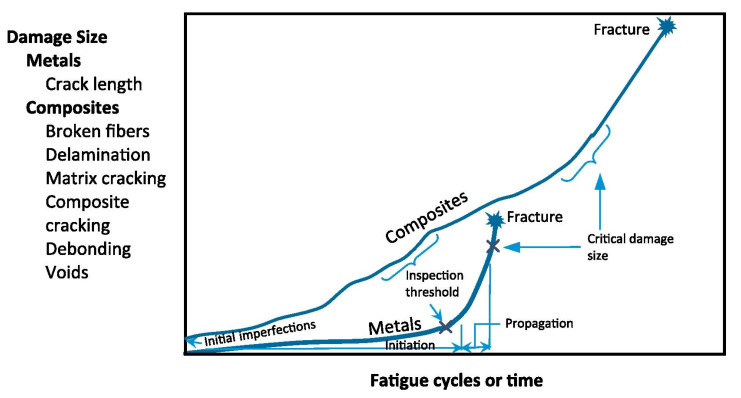
The schematic illustration of the difference between fatigue damage mechanisms between PMCs and metals, adapted with permission from Ref. [64]. Copyright © 2020 Elsevier Ltd.

The fatigue damage mechanisms of the PMC structures may be thoroughly different compared with metallic ones (see Figure 28). Unlike metals wherein crack propagation appears in a predictable way until final failure, damage propagation in the cyclically loaded PMC structures may be unpredictable due to the heterogeneity and anisotropic properties of such materials, as well as the viscoelastic nature of polymers. Noticeably, the critical size of damage in a typical PMC structure (e.g., fiber breakage, delamination, debonding, matrix cracking and voids) is comparatively greater, as opposed to the sizes of metal damage (mainly cracks) [64].

One of the primary open questions associated with evaluating the fatigue performance of FRP composites experiencing the self-heating effect has been as follows for many years:

*What exactly occurs in a cyclically loaded PMC structure with the presence of the self-heating phenomenon at different scales?*

To answer this question, it may be more rational to define the level of structural degradation induced by the self-heating effect in PMC structures at different scales.

### 3.1. Definition of Degradation in Nano-, Micro- and Macroscales

**Figure 29:**
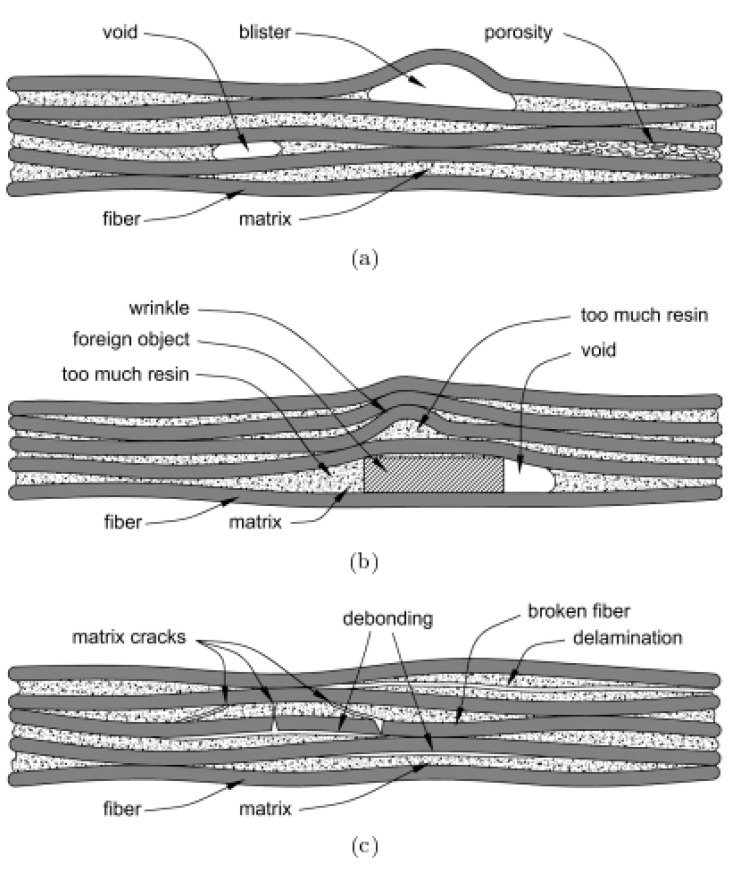
Typical damage in PMCs induced during the manufacturing process due to (**a**) improper curing processing, (**b**) incorrect fiber volume distribution and foreign bodies, and (**c**) insufficient wetting of fibers and lack of adhering between fibers and polymer matrix [98].

In general, a fabricated FRP composite structure before experiencing fatigue loading may possess various types of damage (see Figure 29a,b) which can be classified based on the effects of air entrapment and fiber volume fraction as follows:

- Porosity, blisters and voids caused by air entrapment;
- Resin-rich and -poor zones caused by inhomogeneous distribution of a reinforcement in composites.

**Figure 30:**
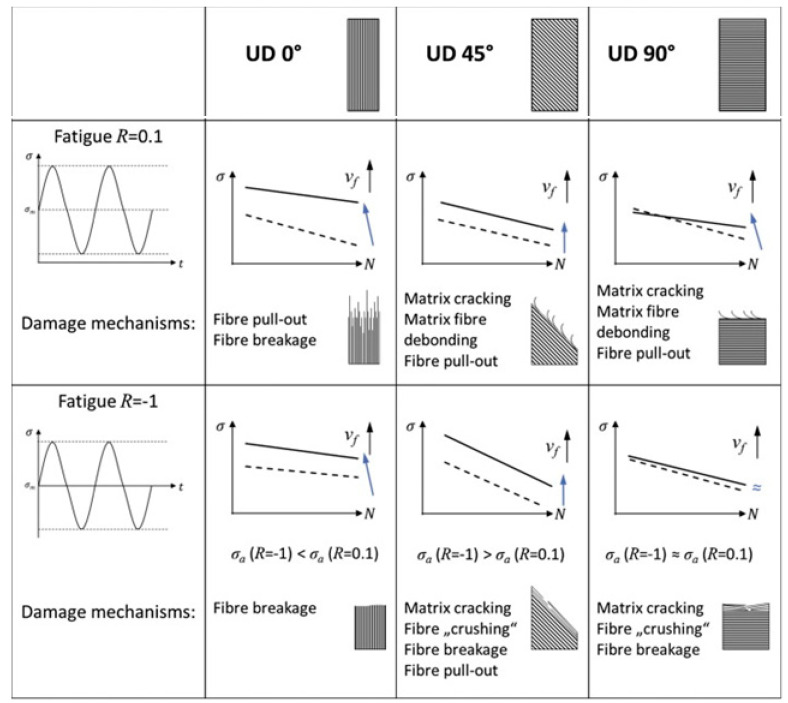
Schematic fatigue damage mechanisms for laminated CFRP composites depending on stress ratio, fiber volume fraction and test angle, adapted with permission from Ref. [101]. Copyright © 2015 Elsevier Ltd.

Although it is assumed that the PMC specimens are devoid of any pre-existing damage before conducting the fatigue tests, such PMC structures may even face a mixed damage types (combination of interface/fiber/matrix damage) during manufacturing process (see Figure 29c):

- Reinforcement and polymer–matrix damage: fiber breakage and matrix microcracks;
- Interface damage: fiber bridging/pull-out, delamination/interlaminar cracking, debonding;

The above-mentioned damage types (see Figure 29c) can appear in a cyclically loaded PMC structure. Regardless of the effect of loading frequency, a typical PMC structure may experience different modes of failure depending on the type of applied loading, stress ratio (*R*), fiber volume fraction (*V_f_*) and stacking sequence (known also as the test angle). For this reason, Zaghloul et al. [99] investigated the influence of fiber volume fraction on the fatigue performance of glass fiber-reinforced polyester at five different stress levels (i.e., 75%, 65%, 50%, 40% and 25%) during tension–tension fatigue testing. They reported that the fatigue strength increased by 100.4% as the result of an increase in the fiber volume fraction from 20% up to 50% subjected to a high stress level. Nevertheless, the fatigue strength increased by only 38.2% when subjected to low stress level as the fiber volume fraction increased from 20% to 50%. In another study, Zaghloul et al. [100] experimentally showed that adding 4% cellulose nanocrystals to a polyester polymer matrix resulted in optimum fatigue performance. They reported that the low fiber volume fraction caused interfacial debonding and matrix cracking, while the high fiber volume fraction resulted in fiber pull-out [99]. Brunbauer and Pinter [101] conducted a comparative study on laminated CFRP composite in order to investigate the effect of the above-mentioned parameters. Samples with fiber volume fractions of 30 and 55% were prepared to be tested at two different levels of R (i.e., *R* = 0.1 and *R* = −1) under three separate test angles (i.e., 0°, 45°, and 90°). They reported that both *R* and *V_f_* play a key role in the type of damage mechanisms (see Figure 30). For example, for a test angle of 0° (i.e., loading is parallel with the fiber direction), the main damage mechanism is fiber breakage for fully reversed fatigue loading (*R* = −1), while both fiber pull-out and fiber breakage were observed under tensile loading (*R* = 0.1). Moreover, fiber crushing was observed under tensile–compressive load (*R* = −1).

The induced damage types during fatigue loading of PMC structures can be classified from nano-, micro- and/or macroscopic points of view as follows.

#### 3.1.1. Nanoscale

The concept of degradation in cyclically loaded FRP composites at the nanoscale can be mainly associated with the dynamic behavior of polymer chains and the interface/interphase between fibers and polymer chains [102] during both stationary (phases I and II) and nonstationary (phase III) self-heating regimes. According to this, the overall performance of a polymer matrix is highly dependent on the macromolecular crosslinking chains, intermolecular forces and dynamic behavior of polymer chains (e.g., chemical degradation of polymer–matrix performance at elevated temperatures) [103].

**Figure 31:**
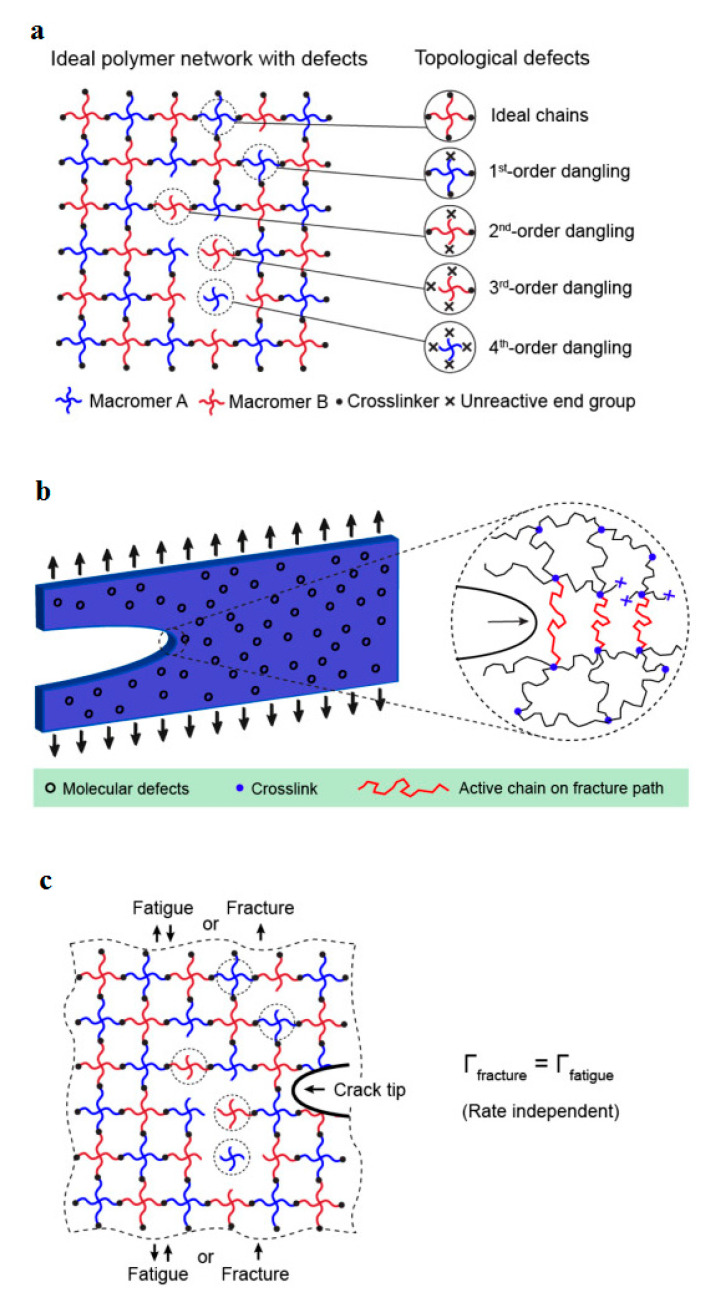
(**a**) Schematic representation of ideal polymer networks involving the imperfection of dangling chains (adapted with permission from Ref. [107]. Copyright © 2021 Elsevier Ltd.). (**b**) Illustration of defect-network fracture model (adapted with permission from Ref. [108]. Copyright © 2020 American Physical Society). (**c**) Schematic illustration of the equality of fracture toughness and fatigue threshold of the ideal polymer networks (adapted with permission from Ref. [107]. Copyright © 2021 Elsevier Ltd.).

Most engineering polymeric materials have randomly crosslinked networks or imperfection structures (e.g., epoxy [104], polyacrylamide hydrogels [105] and elastomers [106]). Structural imperfections involve non-uniform chain length (i.e., the total number of monomers of a chain connected through neighboring crosslinks), non-uniform functionality (i.e., the number of chains connected to a crosslink), chain entanglements and/or uncontrolled topological defects (e.g., dangling chains, cyclic loops) [107]. In this context, Lin et al. [107] investigated the fracture and fatigue threshold, defined as the intrinsic energy needed for fracture of a layer of polymer chains, behavior of ideal polymer networks (i.e., possessing uniform chain length and uniform functionality, without chain entanglement) with controlled dangling chain defect densities (see Figure 31a). Figure 31b demonstrates a schematic representation of the key concept for the defect-network fracture model: a crack propagates by fracturing unaffected polymer chains as well as affected chains due to defects that they used [108]. According to their experimental measurements (Figure 31c), no significant difference was observed between the fracture toughness (*Γ*_fracture_) and fatigue threshold (*Γ*_fatigue_) of an ideal polymer network with low-density dangling-chain defects (i.e., *Γ*_fracture_ = *Γ*_fatigue_). In addition, both fracture toughness and fatigue threshold were independent of the cyclic loading ratio for the ideal polymer networks without chain entanglements.

**Figure 32:**
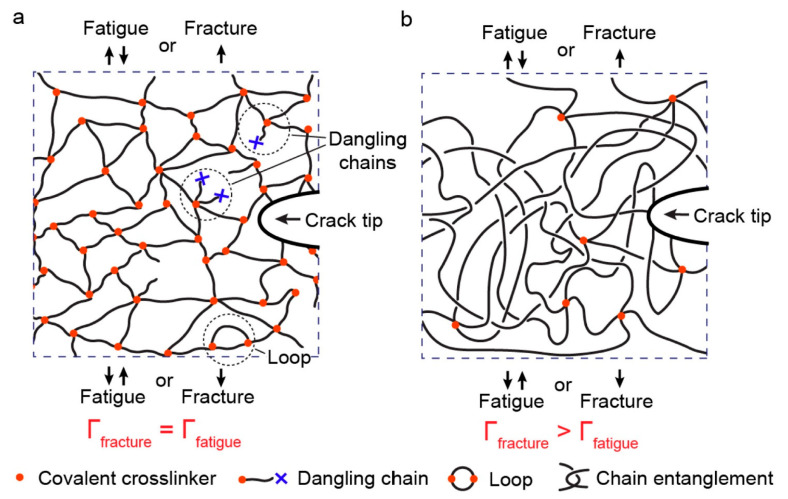
Fracture and fatigue of (**a**) entangled and (**b**) unentangled polymer networks under cyclic loading, adapted with permission from Ref. [110]. Copyright © 2022 Elsevier Ltd.

In another study, Zhang et al. [109] implemented polyacrylamide (PAAm) hydrogels to investigate the influence of chain entanglement on fracture and fatigue of polymer networks under fatigue loading. Although the nearly unentangled polymer networks involve structural heterogeneity (i.e., non-uniform chain lengths and non-uniform functionalities) and topological defects (i.e., dangling chains and cyclic loops), they showed that the equality of fracture toughness and fatigue threshold is valid even in the presence of such structural imperfections (see Figure 32a [110]). Additionally, although the maximum stress–stretch hysteresis ratio of the entangled polymer network (i.e., the ratio of the dissipated mechanical energy to the total mechanical work done to the material) due to bulk dissipation of polymer networks was below 10%, the measured fracture toughness of an entangled polymer network was 16 times greater than its fatigue threshold (Figure 32b [110]). This discrepancy was because of the near-crack energy dissipation.

**Figure 33:**
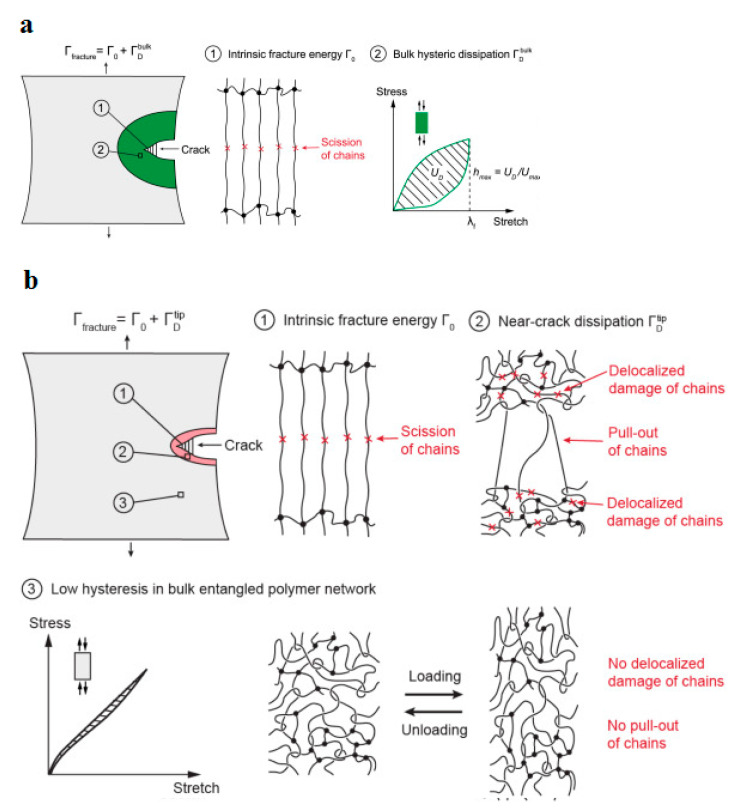
Schematic demonstrations of (**a**) bulk dissipation model and (**b**) the near-crack energy dissipation mechanism for entangled polymer networks, adapted with permission from Ref. [110]. Copyright © 2022 Elsevier Ltd.

The fracture mechanism in an entangled polymer network under cyclic loading–unloading tests can be characterized using the bulk dissipation model [111] and near-crack dissipation. The former model refers to the toughening mechanism by the implementation of the large stress–stretch hysteresis of a bulk material, consisting of two physical processes (see Figure 33a). Firstly, the scission of a layer of polymer chains in the crack path causes the intrinsic fracture energy of the material *Γ*_0_ equal to the fatigue threshold (i.e., *Γ*_D_ = *Γ*_fatigue_). In the second step, material elements in a process zone around the crack experience cyclic loading as the crack propagates, as a consequence of which a significant portion of mechanical energy is dissipated in the form of a hysteresis effect. Thus, the total fracture toughness of a soft material can be described as the sum of bulk hysteretic mechanical dissipation ($\Gamma_{D}^{\mathrm{bulk}}$) and intrinsic fracture energy (*Γ*_fatigue_), i.e., *Γ*_fracture_ = *Γ*_fatigue_ + $\Gamma_{D}^{\mathrm{bulk}}$. According to the latter model (i.e., near-crack dissipation), two physical mechanisms occur during the fatigue loading of soft materials (see Figure 33b). The first process is similar to the former one. During the second physical process, highly entangled polymer chains across the crack plane are pulled out during crack propagation, dissipating substantial mechanical energy because of numerous intermolecular interactions among neighboring chains. Moreover, scissions of chains can be delocalized to multiple adjacent layers around the crack plane as the result of remarkably stretched entangled polymer chains, leading to the dissipation of more energy than fracturing a single layer of chains. However, the pull-out and/or delocalized damage of chains in the bulk entangled polymer network under stretches might be negligible due to the comparatively lower stretch applied on the bulk entangled polymer network before failure rather than the stretch of the crack tip, resulting in relatively small stress–stretch hysteresis of the polymer chains. In the near-crack dissipation model, the overall fracture energy can be defined as the sum of the fatigue threshold and the dissipative fracture energy due to pull-out and/or delocalized damage of chains near the crack tip ($\Gamma_{D}^{\mathrm{tip}}$), i.e., *Γ*_fracture_ = *Γ*_0_ + $\Gamma_{D}^{\mathrm{tip}}$.

**Figure 34:**
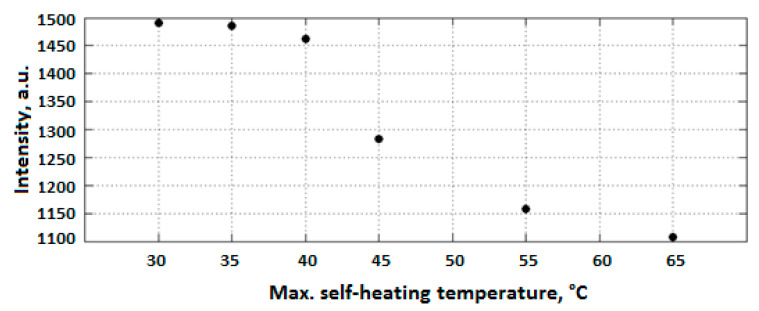
Intensity of Raman band versus self-heating temperature induced during fatigue loading for epoxy [1,113].

Since the induced self-heating effect during fatigue loading may accelerate the dynamic behavior of polymer chains, it is worth discussing the effect of self-heating temperature. In soft materials, particularly amorphous and semi-crystalline PMCs, the rate of temperature growth caused by the self-heating effect during fatigue loading is relatively low when the ratio of maximum measured temperature through the thickness (T) to the glass transition temperature ($T_{g}$) is approximately lower than 0.8 (i.e., $T<0.8T_{g}$) [112]. It should be mentioned that the maximum temperature during cyclic loading normally occurs in the midplane of such a composite specimen, and thus determining it experimentally is very difficult due to the need to insert sensors in the midplane during the specimen manufacturing process. This may even have negative influence on the fracture mechanisms of such structures. To address this issue, it may be rational to link $T_{g}$ with the specimen surface temperature ($T_{s}$), which can be experimentally measured with less complexity using IR thermography, and its value is relatively lower than the maximum temperature in midplane due to exposure to a coolant agent (e.g., air). It can be assumed that when $T_{s}<0.5T_{g}$ (i.e., stationary self-heating mode and lower than critical self-heating temperature [1]), the dynamic modulus of polymer is primarily controlled by bond stretching and bending. This directly reflects the stiffness of van der Waals bonds binding one molecular chain to another [112]. On the other hand, when the temperature induced by the self-heating effect sees a sudden rise (phase III), thermal expansion increases the molecular separation and lowers the van der Waals forces, and consequently bonds begin to be weaker. The polymer chains slide relative to each other, and the crosslinking degree of chains changes, so van der Waals bonds may be melted thoroughly. This can lead to chemical degradation, which is directly connected with structural degradation. For this purpose, Katunin et al. [113] conducted studies for the purpose of analyzing the chemical degradation of glass–epoxy composite specimens under fatigue loading in the presence of the self-heating effect. They measured the residual cross-linking of epoxy using Raman spectra with band of 1256 cm^−1^ at the location of highest mechanical stress concentration for a set of specimens with variable degradation degrees. As can be concluded from the extracted results illustrated in Figure 34, a sudden drop appeared at the maximal self-heating temperature of 45 °C, associated with the residual cross-linking of epoxy in GFRP composite. In another study, Turczyn et al. [114] investigated how the structural and chemical degradation of PMCs are affected by self-heating phenomenon using Raman and FTIR spectroscopy. They reported that no significant structural integrity degradation was observed below the self-heating temperature of 80 °C because of residual cross-linking reactions. By contrast, intensity ratios of peaks degraded by 30% when the temperature rose to 95 °C, which was due to the reduction in the degree of cross-linking chains [115]. This confirms that the domination of thermal effects rather than mechanical effects beyond the temperature of 80 °C influences the structural integrity at lower temperatures; the self-heating temperatures below 80 °C have a negligible effect on the intensities assigned to epoxy groups. Starting from 80 °C, the relative intensity decreases, reaching its minimum at 95 °C. At this temperature, the peak intensity dropped by 30%, probably due to the occurrence of a residual cross-linking reaction.

Furthermore, since the coefficient of thermal expansion (CTE) of polymers is greater than that of fibers, the residual thermal stresses may exist at the interface/interphase region between the fiber and polymer [102]. This may provoke intermittent debonding and cracking in the interphase zone at the submicron scale, and the order of the interphase zone in nanocomposites is mainly in the nanoscale [116,117]. One of the most viable solutions for the interfacial bond between the fiber and polymer matrix is to utilize chemical functionalization [118]. Therefore, measuring the level of remaining crosslinking of the polymer matrix as well as the fiber/matrix interface behavior during fatigue loading with the presence of the self-heating effect will provide comprehensive information for understanding the degree of structural degradation. Since there is no specific study quantifying the influence of the interface/interphase zone on the overall fatigue performance of FRP composites, this can be considered in further studies.

#### 3.1.2. Microscale

**Figure 35:**
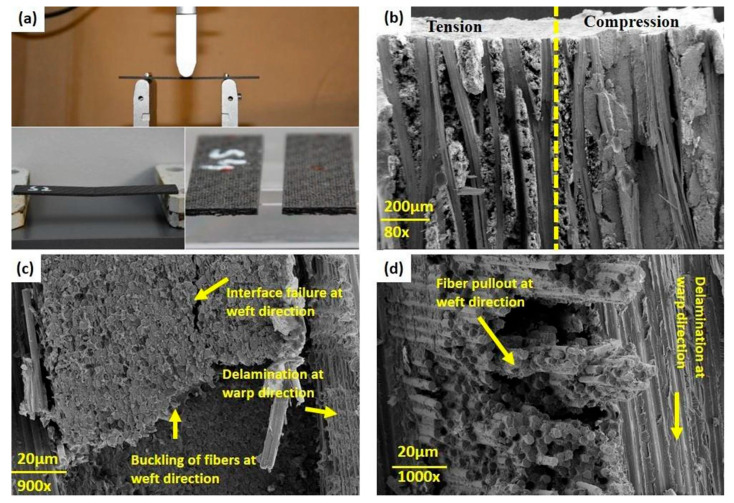
(**a**) Fractured/damaged composite specimen; (**b**–**d**) an illustration of different failure modes induced by three-point cyclic bending loading, adapted with permission from Ref. [119]. Copyright © 2019 Elsevier Ltd.

The failure mechanism of PMC structures under fatigue loading at the microscale consists of crack nucleation, microcracks and open cracks. A good illustration of the different fatigue damage types in the microscale appearing in a CFRP composite, as an example, subjected to cyclic bending loading is presented in Figure 35 [78].

**Figure 36:**
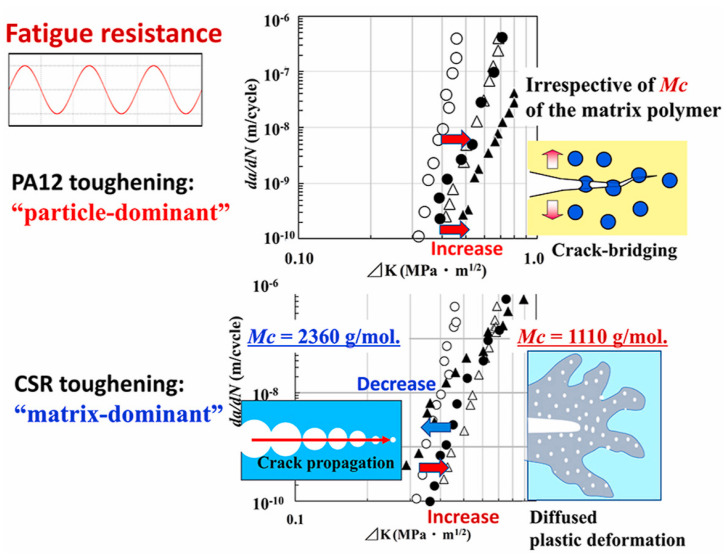
An exemplary illustration of the fatigue crack propagation (FCP) and fatigue threshold of moderately and highly toughened epoxy blends with PA12 and CSR polymer particles, adapted with permission from Ref. [120]. Copyright © 2021 Elsevier Ltd.

Regardless of the effect of voids and defects during the fabrication process, the level of crack nucleation is highly dependent on the stress intensity factor ($K_{I}$), which is a function of the topography of surfaces or interfaces [26]. For this reason, Kishi et al. [120] studied the fatigue threshold and fatigue crack propagation (FCP) of toughened epoxy blends with polyamide 12 (PA12) and core–shell rubber (CSR) polymer particles. They reported that unlike fatigue thresholds of toughened epoxies with PA12 particles which were induced by the greater stress intensity factor (Δ*K*) values, fatigue thresholds of epoxies modified with CSR polymer particles were influenced by molecular weight between crosslinks (*Mc*) (see Figure 36). The gently (*Mc* of 2360 g/mol) and highly (*Mc* of 1110 g/mol) crosslinked CSR-toughened epoxy blends were extracted from comparatively lower and higher Δ*K* ranges, respectively, as opposed to pure epoxy blends. Crack bridging appeared in toughened epoxy blends with PA12 due to particle dominance, while crack propagation took place in toughened epoxy blends with CSR particles because of matrix dominance.

**Figure 37:**
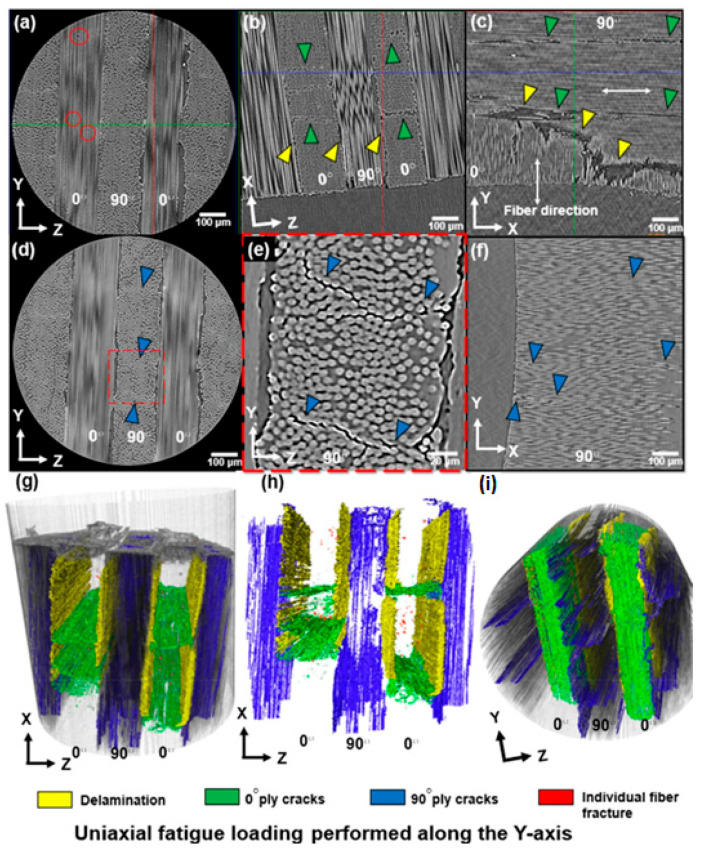
The 3D imaging and segmentation of the milled CFRP sample after fatigue loading process (**a**) fiber breakage in ZY plane, (**b**) combination of 0° ply cracks and delamination in ZX plane, (**c**) delamination at the interface between two orthogonal adjacent plies in XY plane, (**d**) ZY plane showing another location on 90° ply with interfiber cracking magnified in (**e**), (**f**) 90° ply cracking running horizontally in XY plane, (**g**–**i**) segmented volume renderings showing the ZX plane region of interest with minimal fiber damage on cut surface (top) and all the mechanisms in play, adapted with permission from Ref. [121]. Copyright © 2021 Elsevier Ltd.

Furthermore, Rose et al. [121] studied the damage mechanisms of the milled CFRP and the laser-cut specimens under fatigue loading. In their investigation, they revealed that although the milled CFRP specimens showed some common failure modes with laser-cut samples, such specimens experienced some different failure modes as well. Figure 37a–c demonstrate the 2D virtual cross-sections in which single-fiber breakage (red circles), delamination (yellow arrows) and 0° ply cracks (green arrows) are visible. Although the 0° ply cracks were closely similar to those demonstrated in the remote laser-cut specimens, such cracks showed relatively smaller crack opening displacement and comparatively lower fiber breakage and reorientation within the ply. This specimen was also investigated in the transverse direction (90° ply cracks). Figure 37d–f illustrate these cracks on the three orthogonal planes. It was matrix cracking that took place in areas of the ply consisting of a higher density of fibers in the ZY plane, and Figure 37g–i reveal volume renderings in different planes. The ZX plane demonstrates a zone with minimal fiber damage on the cut surface (top) and all the mechanisms at play.

**Figure 38:**
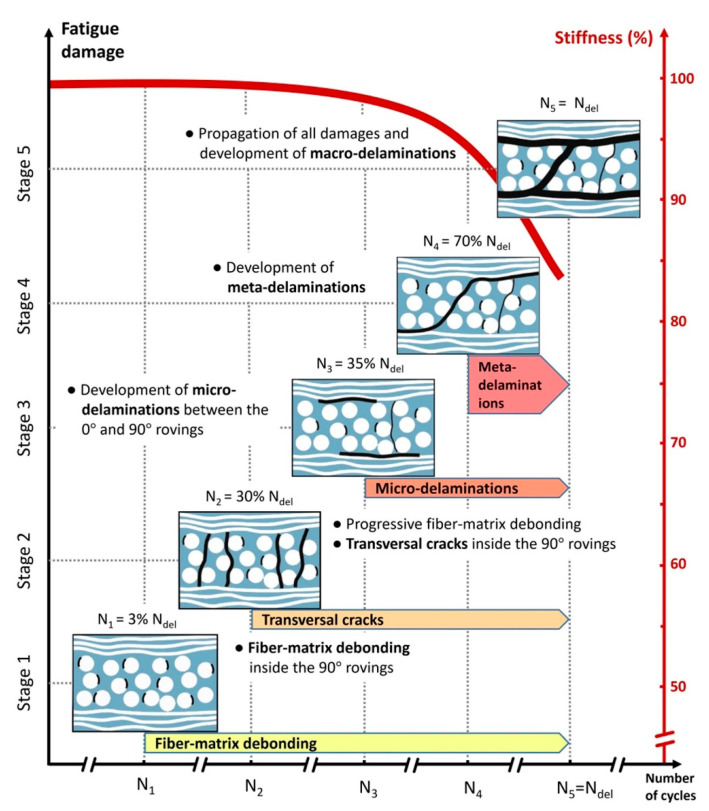
Characterizing fatigue damage propagation of CF-PPS within different stages, adapted with permission from Ref. [58]. Copyright © 2021 Elsevier Ltd. [123].

The formation of microcracks and microdelaminations, and consequently macrocracks, in PMC structures can be taken into account as a continuous process, which can be intensified by loading conditions. To put it differently, according to the Clausius inequality statement (second law of thermodynamics), the gradient of generated entropy and intrinsic dissipation in an irreversible thermodynamic process would be constantly positive. As the result of produced entropy induced by the self-heating effect, a cyclically loaded PMC structure may experience an irreversible microstructure evolution or degradation regardless of whether a high magnitude of frequency or stress is applied. These irreversible alternations in the microscale will collectively result in entropy generation, so the entropy generation rate can remarkably change based on the amplitude of frequency or stress. In other words, as such a PMC structure is subjected to a higher frequency regime or stress/strain ratio, a greater level of entropy and consequently greater temperature value will be generated. High structural degradation in the microscale or stiffness reduction can be directly explained by an increase in the entropy generation rate [122]. For the purpose of characterizing the structural degradation of PMC structures, Balle and Backe [123] investigated the fatigue fracture mechanisms of carbon fiber-reinforced polyphenylene sulfide (CF-PPS) laminated composites by computing stiffness degradation under the VHCF regime wherein the loading frequency, maximum shear stress and load ratio were considered to be 20 kHz, 8 MPa and 0.51, respectively. During the fatigue fracture process of such a PMC specimen, they reported five separate phases, which are schematically illustrated in Figure 38. The first stage of the process was explained by the initialization and consequent accumulation of fiber–polymer interface debonding induced by the comparatively high shear stress value in the transverse direction of carbon fibers, followed by second phase, wherein the rate of fiber–matrix debonding increased, as the result of which the first cracks in transverse direction of carbon fibers appeared. CF-PPS specimen life was reduced by just below one-third (30%) due to being occupied with such damage. During the third phase, the appearance of microdelaminations between the longitudinal and transverse directions of reinforcement was reported, approximately after 35% of the entire CF-PPS specimen’s lifetime, meaning less than 5% of degradation occurred in this stage involving crack lengths of 9 μm or below. This comparatively slow damage propagation and consequent stiffness degradation trend can be explained by the appearance of crack density saturation and delaminations in the interface of fiber and polymer chains [124]. The fourth stage took place when the metadelaminations propagated, occurring after approximately the two-thirds (70%) of the entire specimen fatigue life, containing maximum crack lengths of about 25 μm or less. It should be mentioned that the crack length which provokes the failure is highly dependent on the dimension of the specimen. For example, when the dimension of the structure is high, the crack length of 25 μm may not cause failure.

**Figure 39:**
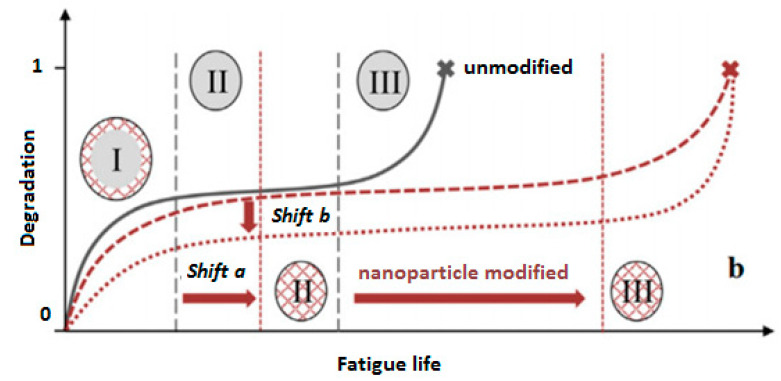
A schematic representation of extending fatigue life of CFRP composite led by adding through an extension of phase I and II (shift a) and a less pronounced degradation increase (shift b), adapted with permission from Ref. [125]. Copyright © 2014 Elsevier Ltd.

As a guideline, it should be mentioned that when the size of the crack is microscale, using nanoparticles such as CNTs can extend the fatigue life of the PMC structure. Figure 39 schematically reveals how the fatigue life of a CFRP composite can be extended as the result of adding CNT to a polymer [125]. The black line depicts the degradation process of an unmodified polymer matrix, while the dotted red lines illustrate the degradation mechanisms of a matrix modified with CNTs. It is noteworthy to conduct comprehensive studies in this area due to its practical importance. For example, depending on the loading conditions and geometry, the influence of adding different nanoparticles can be investigated.

#### 3.1.3. Macroscale

The accumulation of the above-mentioned micro damage types (stages I to IV in Figure 38) in the PMC structure can continually develop macrocracks and delaminations [126], resulting in the ultimate failure of the material (stage V in Figure 38). Within the final stage, a high level of stiffness degradation is observed, which can be justified by the formation of macrodelaminations. Since the growth rate of fatigue damage accumulation was very high in this stage, structural failure occurred, which is also named sudden death. In general, the occurrence of ultimate failure is the direct consequence of the contributions of mechanical fatigue loading, thermal gradients, chemical ingress and environmental conditions [26]. Nevertheless, in such a problem, stages IV and V (i.e., the main stages contributing to the failure) may be justified by the generated high self-heating temperature values induced during fatigue loading on the surface of the composite specimen $\left(\Delta T>20\;{}^{\circ}C\right)$.

**Figure 40:**
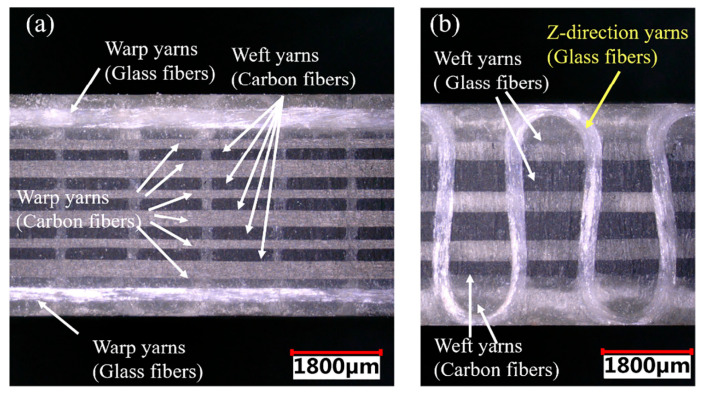
The front (**a**) and back (**b**) cross-sections of the 3D hybrid composite from the weft yarn direction views, adapted with permission from Ref. [127]. Copyright © 2019 Elsevier Ltd.

Using a 3D microscope for the purpose of monitoring failure propagation and morphologies, Fan et al. [127] investigated the fatigue behavior of a 3D orthogonal carbon/glass fiber hybrid composite under three-point bending fatigue. Figure 40 depicts the front and back cross-sections of the used 3D hybrid composite from the weft yarn direction views.

**Figure 41:**
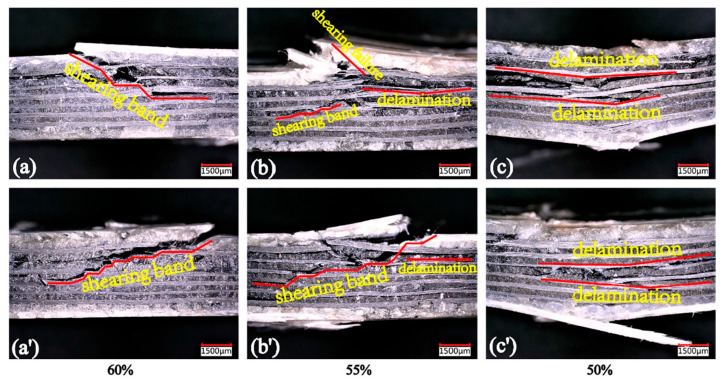
Exemplary fatigue failure mechanism of a laminated hybrid PMC structure under different stress levels: (**a**,**a’**) 60%; (**b**,**b’**) 55%; (**c**,**c’**) 50%, adapted with permission from Ref. [127]. Copyright © 2019 Elsevier Ltd.

The exemplary ultimate fatigue failure morphologies of the laminated hybrid composite along with warp yarn directions on a macroscopic scale at three different stress levels, namely (a) and (a’) 60%, (b) and (b’) 55%, (c) and (c’) 50%, are presented in Figure 41. According to the results extracted for the laminated hybrid composite, a shear damage band was observed in the specimen subjected to the stress level of 60%, while both shear damage and local delamination were captured in such a specimen when subjected to 55% of the maximum stress. When the stress levels decreased to 50%, the damage of the composite slowly propagated to the entire region area of such a specimen in the form of delamination. This means that the relatively lower stress was unable to cut the fibers at one time, leading to propagation of the crack parallel to the longitudinal direction of the fibers.

**Figure 42:**
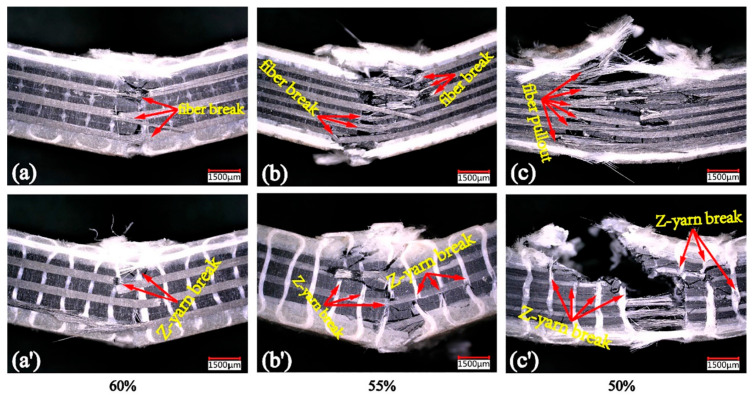
Exemplary fatigue failure process of a 3D hybrid PMC structure under different stress levels: (**a**,**a’**) 60%; (**b**,**b’**) 55%; (**c**,**c’**) 50%, adapted with permission from Ref. [127]. Copyright © 2019 Elsevier Ltd.

Similarly, lateral fracture morphologies of the 3D hybrid composite in the macroscopic scale at three different stress levels, namely (a) and (a’) 60%, (b) and (b’) 55%, (c) and (c’) 50%, are presented in Figure 42. Figure 42a–c illustrate the failure of the surface without the Z-direction yarns, while Figure 42a’–c’ denote the surface damage involving the Z-binder yarns. The results showed that the stress level of 50% was reported to be comparatively the most critical condition for both laminated and 3D hybrid composites. This can be explained by how the greater stress level provoked the failure without damage evolution. Due to the Z-direction yarn-controlled propagation of the crack in the longitudinal direction of fibers, no delamination damage was monitored.

**Figure 43:**
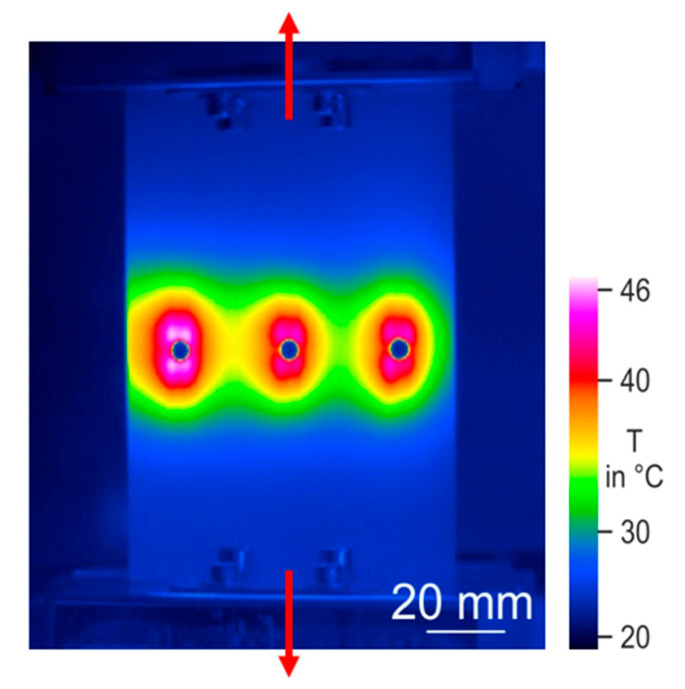
Self-heating temperature profile induced by the cyclic fatigue test extracted from thermography, adapted with permission from Ref. [121]. Copyright © 2021 Elsevier Ltd.

On the other hand, drilled holes are macrodefects, leading to reduction in the ultimate strength of a PMC structure by 50, depending on the stacking sequence [128]. Due to the stress concentration near the areas of holes, the self-heating effect may be comparatively more critical in such regions. An exemplary thermographic image for a cyclically loaded CFRP specimen involving open holes with stress concentrations dominated by self-heating temperature is presented in Figure 43 [121]. Since PMC structures may consist of holes in their real lifetimes, it is noteworthy to conduct further studies in this field.

### 3.2. Criticality of the Self-Heating Effect in Polymers and PMCs

**Figure 44:**
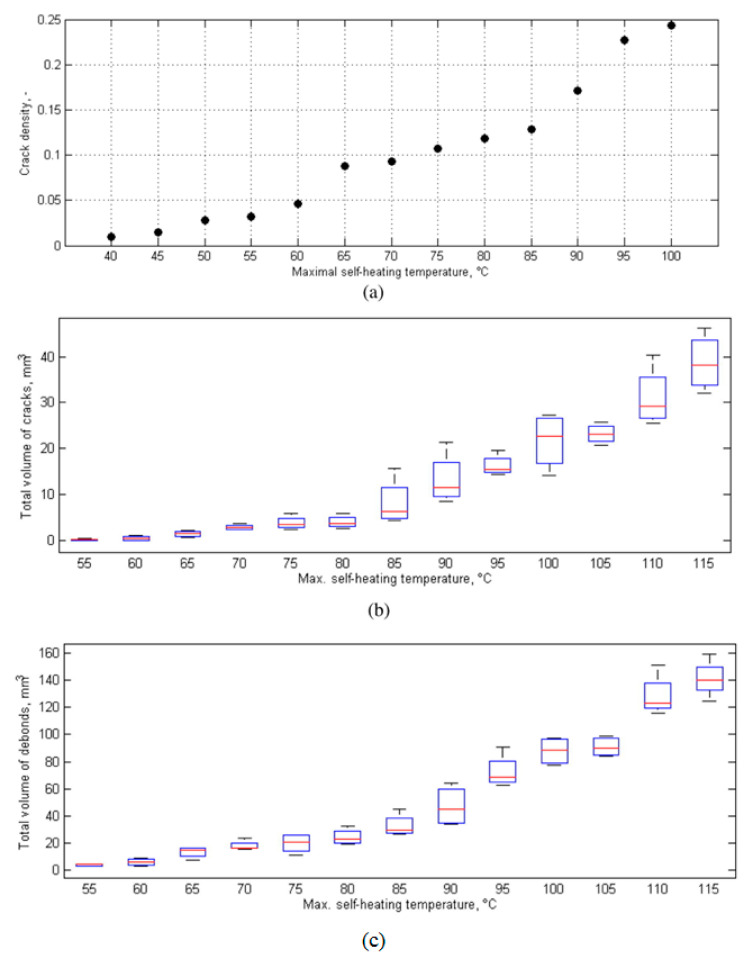
(**a**) Crack density [1], (**b**) total volume of cracks and (**c**) total volume of debonds induced by fatigue loading dominated by self-heating temperature, adapted with permission from Ref. [3]. Copyright © 2017 Elsevier Ltd.

The concept of the critical self-heating effect was initially introduced by Ratner et al. [129], who described it as a critical stress at thermal fracture or a critical temperature value appearing under this critical stress. Moreover, Gumenyuk et al. [130] explained the criticality of the self-heating effect by a remarkable temperature growth beyond which the self-heating temperature history profile becomes unstable, provoking softening in viscoelastic materials. Katunin [131] described the self-heating effect as a measure of degradation degree of a PMC structure subjected to cyclic loading (e.g., bending fatigue). Later, Kahirdeh and Khonsari [132] developed a similar criterion based on thermal and AE responses for characterizing the structural degradation criticality of PMCs under fully reversed bending fatigue loading. In general, the critical self-heating in PMCs can be defined as the temperature at which damage initiates, and accordingly below which such PMC specimens can be safely maintained [1]. The critical self-heating temperature value can be determined using a variety of techniques, reviewed in [1]. As tested for GFRP composites by the authors, the most effective of these techniques are X-ray ICT, morphological analysis (SEM), approximation of self-heating temperature history curves and variability of self-heating temperature distributions. Katunin and Wronkowicz [3] performed fatigue tests for GFRP specimens until obtaining the critical self-heating temperature value on the tested specimens’ surfaces using X-ray CT scans. They proposed a general phenomenology for characterizing the structural degradation of PMC structures dominated by the self-heating effect. They reported that a higher temperature obtained on the specimen’s surfaces induced by the self-heating effect resulted in higher crack density, volume of cracks and volume of delaminations (see Figure 44). Thus, critical self-heating temperature can act as a catalyst in terms of accelerating the volume of cracks and delaminations in a PMC specimen under fatigue. This increase in crack and delamination densities will provoke the formation of macrocracks or structural degradation at the macroscale. This can also be presented in the form of residual stiffness, which can be implemented as input for FEM software packages for further mechanical analysis [133]. To the best of the authors’ knowledge, cracks and delaminations exist below this temperature, but they do not have influence on the mechanical properties of a considered structure and can be negligible.

**Figure 45:**
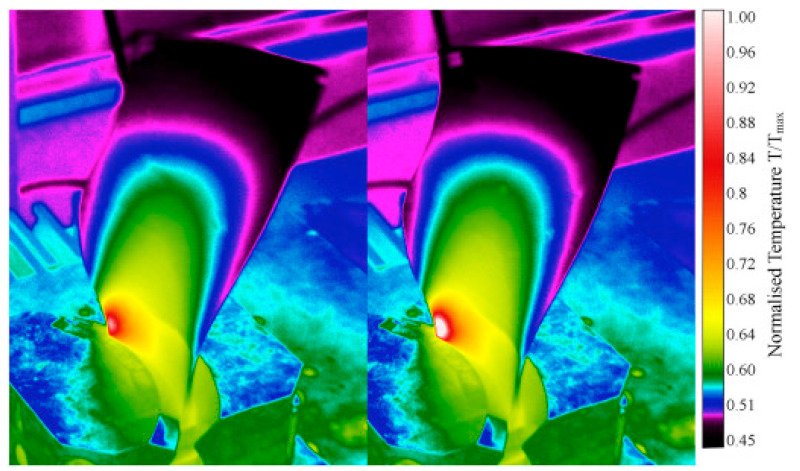
An exemplary illustration of dominating self-heating process in a composite blade: (**left**) before and (**right**) after the occurrence of the critical self-heating temperature, adapted with permission from Ref. [28]. Copyright © 2016 Elsevier Ltd.

Furthermore, Magi et al. [28] proposed a model for monitoring the structural degradation of a laminated composite blade under fatigue loading, working based on critical self-heating temperature. In other words, the rate of structural degradation at a given excitation level suddenly changed as the critical self-heating temperature appeared on the specimen surface (see Figure 45). As the recommendation, the specimen surface temperature should be monitored and controlled to experience a safe range of temperature (below the critical self-heating temperature). Otherwise, thermal fatigue failure may occur before mechanical failure, which is undesirable. For this objective, the viable solutions are deeply reviewed and elaborated in Section 4.

### 3.3. Application of the Self-Heating Effect in Nondestructive Testing (NDT)

**Figure 46:**
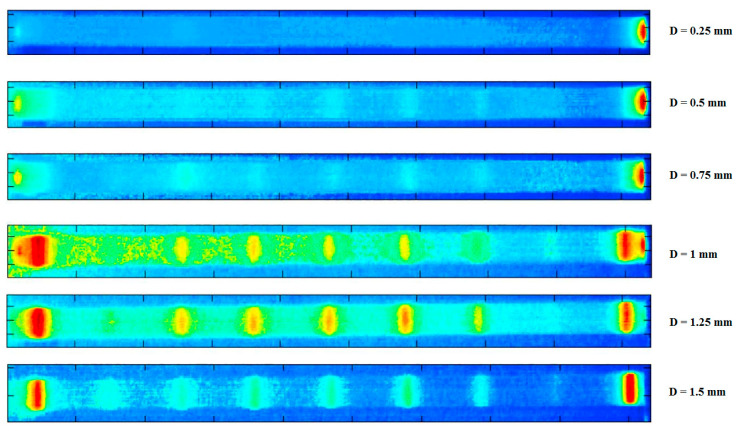
The exemplary results of defect detectability for notch-type damage with different depths using the SHVT method, adapted with permission from Ref. [138]. Copyright © 2018 Elsevier Ltd.

Although the self-heating effect may negatively provoke structural degradation, this concept has been interestingly employed for a variety of engineering applications involving NDT damage characterization in CFRP composites [28,134]. This concept was coupled with classical vibrothermography as an NDT technique for detecting debonding-like defects in a hybrid plate (aluminum alloy glued with a cork plate) [135] and damage detection in polymer-based structures [136,137]. Additionally, Katunin and Wachla [138] developed in-house self-heating-based vibrothermography (SHVT) as an accelerated NDT technique based on the excitation of a structure at multiple resonant frequencies [139] for monitoring fatigue cracks and damage in such structures. As a result of the cyclic mechanical excitation of such a structure, a noticeable amount of mechanical energy is dissipated, which highly depends on the magnitude of resonant frequencies. In this way, the higher magnitudes of resonant frequencies of such a structure are excited, the higher amount of thermal energy will be stored due to the viscoelastic behavior of the polymer. The extracted thermal energy is the primary source of the self-heating phenomenon (as was elaborated earlier). Vaddi et al. [140] also implemented SHVT for detecting fatigue damages in viscoelastic polymer coatings. It should be mentioned that SHVT can be implemented in a wide range of frequencies. For example, Katunin and Wachla [138] implemented SHVT in a comparatively low frequency regime (varying from 0 to 1.25 kHz), while Vaddi et al. [140] employed it for high frequency loading (ranging from 19 kHz to 38 kHz). Figure 46 depicts the exemplary results of damage detectability enhancement using the SHVT technique for notch-type damage with different depths (D), ranging from 0.25 mm to 1.5 mm. As can be seen, the thermal signatures of the introduced defects were completely detectable when the depth of defects was 1 mm or more, which can reveal the effectiveness of the developed approach [138]. It should be mentioned the temperature appearing on the specimen surface during the application of the SHVT technique is significantly lower than the critical self-heating temperature, avoiding premature thermal failure. Furthermore, Maio et al. [141] implemented the SHVT technique for the purpose of monitoring the dynamic properties of laminated CFRP composites during the crack propagation caused by fatigue.

## 4. Concepts of Preventing Structural Degradation in Cyclically Loaded PMCs

As shown in previous sections, the appearance of the self-heating effect can significantly shorten the structural lifetime, which is especially important during fatigue testing of polymers and PMCs. Therefore, a limitation of the influence of the self-heating effect on a structure subjected to fatigue loading is of primary interest in numerous applications. In this section, two different concepts consisting of cooling scenarios and optimal materials selection and design are analyzed for the purpose of preventing or limiting the intensive structural degradation induced by the self-heating effect in both polymers and FRP composite structures under high-magnitude vibrations or fatigue loading.

### 4.1. Cooling Scenarios and Techniques Considering Thermodynamics

Investigating the effect of different cooling scenarios in cyclically loaded polymer–matrix structures is of key interest and practical importance since this topic has not been studied deeply yet. When the value of the surrounding temperature during the fatigue testing of a polymeric or composite specimen is greater than room temperature (RT), it plays the role of a heating system, which intensifies the self-heating effect due to thermal energy generation caused by such a high temperature. This will consequently provoke a noticeable level of structural degradation in PMCs. Therefore, the initial assumption when implementing a cooling scenario is to conduct such high-magnitude vibration excitations or fatigue tests at RT or below. In addition, to control surrounding temperature, it is also of paramount importance to monitor and limit the polymeric specimen surface temperature during such cyclic tests in order to prevent the structural degradation induced by the self-heating effect. For this objective, the influence of cooling scenarios in terms of environmental and operational aspects in cyclically loaded PMC structures is discussed, allowing us to define efficient coolant systems for such tests.

#### 4.1.1. Environmental Factors

Regarding the conditions in which such structures are planned to be implemented, the structures may experience different humidity levels and operating temperatures even before being subjected to any mechanical fatigue loading. The moisture content [142] and ambient temperature can be assumed to be the environmental factors that may have an influence on the degradation of polymeric structures under such cyclic tests. A good illustration of this can be found in marine applications involving static off-shore structures and sea-going vessels [143,144]. This may call into question whether moisture content has a negative influence on the mechanical performance of such structures. For this purpose, Chen et al. [145] experimentally investigated how the cyclic softening/hardening and mechanical energy dissipation of polyamide-6 (PA6) under different applied strain amplitudes at room temperature (RT) are affected by the relative humidity (RH) levels. They showed that the lower RH level relatively resulted in a noticeable temperature increase on the specimen surface. In addition, as the level of RH increased, the level of recoverable viscoelastic deformation increased. Moreover, they illustrated that the average stress relaxation of PA6, regardless of the level of RH, took place in a symmetric cyclic loading with mean tensile strain so that its magnitude saw a reduction as RH increased, while it increased as the result of increasing mean strain. By contrast, the mean temperature and cyclic softening/hardening of PA6 were affected insignificantly by the applied mean strain. In another study, Gholami et al. [146] developed a micromechanical finite element method (FEM)-based degradation model in order to investigate how the elastic properties of FRP composites are influenced by the hygrothermal conditions. They revealed that increasing both temperature and humidity simultaneously provoked a significant reduction in the transverse Young’s modulus and shear modulus of such polymeric specimens. In other words, when an FRP composite was tested under the RH of 95% and the operating temperature ranging from 23 °C to 150 °C, for merely a period of one hour, the transverse Young’s modulus and shear modulus of such a composite were degraded by 90% and 80%, respectively. By contrast, during the desorption stage, although such mechanical properties were also initially degraded because of the extreme temperature value, the stiffness (Young’s moduli) of such a composite increased as the direct consequence of the acceleration of the moisture desorption mechanism. Nonetheless, when such structures are subjected to the high cyclic fatigue regimes or extremely high-magnitude vibration excitations, they may experience relatively high temperature values on their surfaces due to presence of the catastrophic self-heating phenomenon (e.g., rocket propellant composites [22] or a driver shaft [35,36]). In such applications unlike marine ones, moisture may even have an advantageously positive influence, playing the role of a coolant agent for such structures (with the assumption of limiting the presence of moisture content merely on the polymeric specimen surface, but not inside a structure); as a result, the level of structural degradation of such structures may favorably illustrate a downward trend. This leads to the following observation: as the area of a cyclically loaded PMC structure exposed to the humidity of surrounding directly (the area between two grips) and indirectly (the regions located in holding grips) increases, the temperature growth occurring on the specimen surface may decrease [145]. As the direct consequence of this, a relatively lower structural degradation may occur. Further research is required to effectively quantify how the structural degradation of cyclically loaded PMC structures dominated by the self-heating effect are influenced by the moisture content.

In [87], Katunin and Wachla discussed how the ambient temperature can take a center stage as a causative factor contributing to the structural degradation of PMC structures under cyclic loading, which may have a significant influence on the overall mechanical and fatigue performance of operating structures. In general, variation in ambient temperature can play the role of a coolant system or a heater. For this purpose, Charalambous et al. [147] employed the four-point bending test in order to investigate the fatigue performance of CFRP composites under various ambient temperatures (−50 °C, 20 °C, 50 °C and 80 °C). The authors demonstrated that the ambient temperature may act as a catalyst in accelerating or postponing the influence of delamination growth in PMCs, which may be highly dependent on whether the loading belongs to a quasi-static or fatigue loading regime. They reported that the delamination rate of such a composite under fatigue test saw an upward trend as the direct consequence of an increase in ambient temperature. Additionally, the rate of crack propagation at 80 °C was twice greater than the same figure at RT for an unchanged normalized energy release rate value. Voudouris et al. [27] experimentally studied how the failure time of cyclically loaded CFRP composites dominated by the self-heating effect is influenced by different elevated ambient temperatures (25 °C, 50 °C, 65 °C and 75 °C). As they reported, the propagation of delamination was comparatively faster as the result of greater ambient temperature under the fixed excitation frequency and strain values.

**Figure 47:**
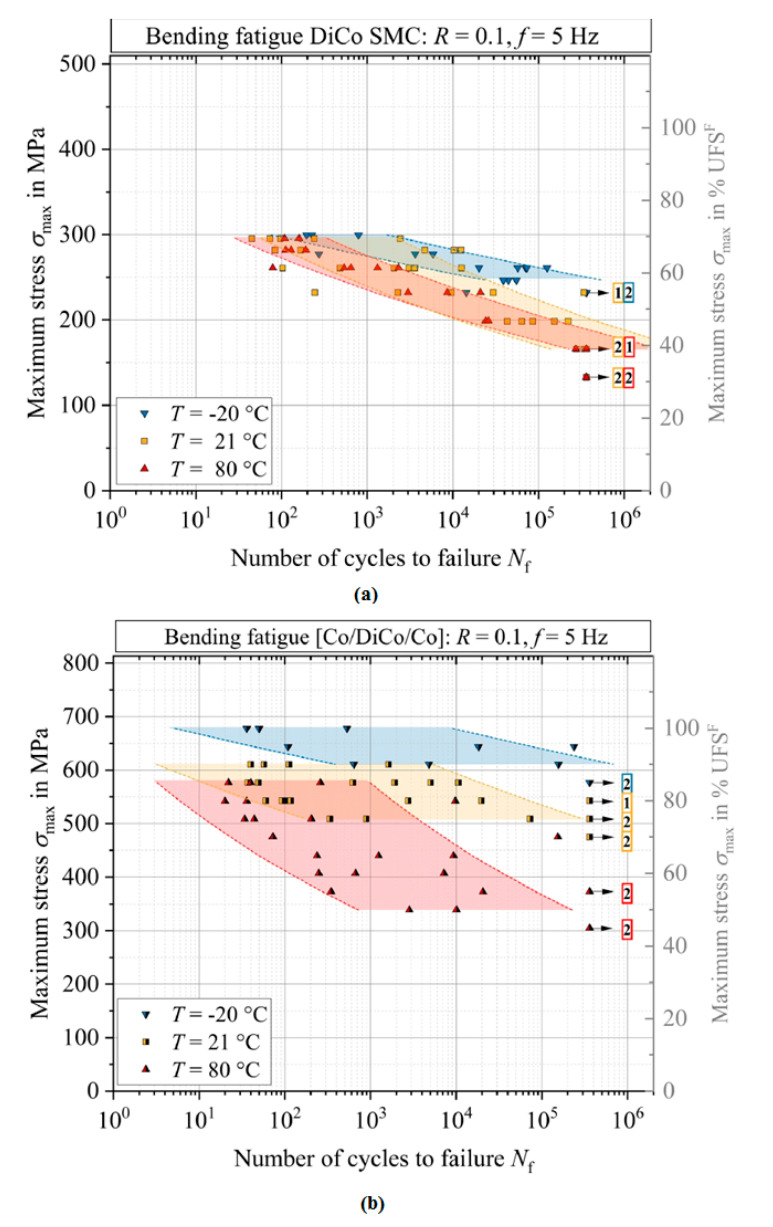
S-N data for (**a**) DGF-reinforced SMC composite specimens and (**b**) hybrid [UCCF/DGF/UCCF] SMC specimens under 3-point bending fatigue load at different temperatures for failure probabilities of *PS* = 10% and *PS* = 90% [17].

If the ambient temperature is lower than RT, this can function as a cooling phenomenon to prevent intensive structural degradation in cyclically loaded PMC structures. Thus, industries and researchers can come forward with viable solutions by providing a cooler ambient temperature in order to address the catastrophic self-heating phenomenon resulting from either fatigue loading or high-magnitude vibration excitations. Regarding providing the system with a relatively low temperature for such experiments, one of the viable solutions is to carry out fatigue tests on samples exposed to different gases, e.g., CO_2_. For this purpose, Charalambous et al. [147] implemented a carbon dioxide (CO_2_) skid comprising 15 cylinders of liquid CO_2_ gas attached to the environmental chamber through a decanting hose to provide the temperature of −50 °C for fatigue testing up to 10^6^ cycles with a frequency of 5 Hz, that is, approximately 57 h for a fatigue test. To effectively address the tendency of formation of an ice layer on the grooved roller surface, anti-icing liquid was used on the rollers and the end-tab area. An unimportant difference regarding the mean bending moment was needed for propagating delamination to observe the results induced at room temperature and at −50 °C. During the fatigue experiments, the gas injection took place in regular intervals in order to keep the temperature at the constant value of −50 °C, they exploited the large bending moment drops following delamination onset. The main causative factor contributing to this can be explained by more a brittle polymer matrix induced by low temperature, leading to increased stick–slip behavior [148]. In another study, Bartkowiak et al. [17] investigated how the three-point bending fatigue properties of hybrid sheet molding compound (SMC) composite-reinforced with discontinuous glass fibers (DGF) in the core and unidirectional continuous carbon fibers (UCCFs) in the face layers up to $2.6\times 10^{6}$ cycles are influenced by different ambient temperatures (−20 °C, 21 °C and 80 °C). They implemented liquid nitrogen (N_2_) as the coolant agent for the purpose of providing a temperature of −20 °C for experiments. They exploited that increasing the ambient temperature from 21 °C to 80 °C for DGF-reinforced SMC and hybrid [UCCF/DGF/UCCF] SMC samples provoked a remarkable reduction in flexural strength by 24% and 7% at a quasi-static strain rate (2 mm/min) and 14% and 12% at a fatigue strain rate (90 mm/min), respectively. Nevertheless, decreasing the ambient temperature to −20 °C resulted in increases in flexural strength by 2% and 3% for quasi-static strain and fatigue strain rates, respectively. They also revealed that when the temperature of the specimen was reduced from 21 °C to −20 °C using liquid nitrogen, the flexural strength of the hybrid SMC composite experienced a downward trend, which was increased by 25% and 14% in quasi-static and fatigue strain regimes. The S-N results obtained for DGF-reinforced SMC composite specimens and hybrid [UCCF/DGF/UCCF] SMC specimens under three-point bending fatigue load (*R* = 0.1 and *f* = 5 Hz) exposed to three separate temperatures with failure probabilities of *PS* = 10% and *PS* = 90% are illustrated in Figure 47 [17]. As can be seen from Figure 47a, the fatigue life expectancy of DGF-reinforced SMC specimens exposed to a lower temperature (−20 °C) was comparatively longer than that of specimens subjected to higher temperature values. When such composites were subjected to temperature values of 21 °C and 80 °C, they revealed roughly similar fatigue behavior. According to Figure 47b, the residual fatigue life of hybrid [UCCF/DGF/UCCF] composites was highly dependent on ambient temperature; i.e., with the decrease in the temperature such a hybrid composite was exposed to, there was an increase in the fatigue life obtained. For example, the fatigue limit of such composites subjected to the liquid nitrogen at −20 °C was roughly 100 MPa higher than such data obtained at RT. Therefore, implementation of liquid nitrogen as a coolant agent has the capability to extend the fatigue life of cyclically loaded discontinuous and/or continuous FRP composites.

#### 4.1.2. Operational Factors

**Figure 48:**
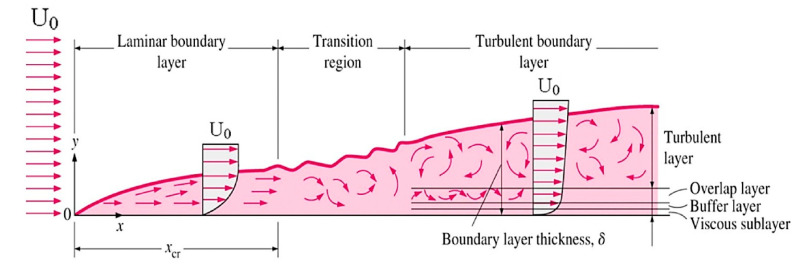
Different types of flows, adapted with permission from Ref. [150]. Copyright © 2017 Elsevier Ltd.

There are a number of operational parameters associated with the coolant systems which might actively function as a catalyst for avoiding or at least minimizing the structural degradation in polymers and PMC structures, which are associated with the average convective heat transfer coefficient ($\bar{h}$) of the fluid around a composite structure. In 1883, Osborne Reynolds illustrated two different fluid flow regimes, namely laminar and turbulent [149]. However, the laminar flow mainly experiences a transition state to be converted into a turbulent stream (see Figure 48). In laminar flow, the fluid layers slide smoothly over each other, taking place normally in low-velocity regimes, while turbulent flow is defined as a complex process in which erratic motion and eddies in different sizes are superimposed onto the streamlines [150]. Additionally, the behavior of a transient stream may be quite similar to that of a turbulent stream.

The Reynolds number, associated with this behavior, can be defined as the ratio of inertial to viscous forces of the fluid [151], depending on four parameters, namely viscosity (μ), speed ($U_{0}$), density (ρ) and characteristic dimension (D) (see Equation (16)).

(16) Re=Inertia ForcesViscous  Forces=ρU0Dμ

Increasing viscosity will lead to a reduction in the Reynolds number, while increasing the value of each three other parameters will lead to an increase in this number. It should be mentioned that for pipes, *D* is the diameter of a cylinder, while for plates, *D* is the length or width of a plate which is parallel to the direction of the fluid. In the case of a pipe or cylinder, if the Reynolds number is below 2300, the fluid particles tend to behave as a laminar flow extracted from smooth and constant fluid motion due to adequate viscous force. Otherwise, the propagating rate of eddies, vortices and other flow instabilities may be intensified, leading to a turbulent flow. However, the critical Reynolds number for a flat plate geometry, which is implemented primarily for cyclic fatigue tests, is 5 × 10^5^ [152]. Otherwise, a transient and/or turbulent flow may take place.

The following is still an open question: how can the generated thermal energy level induced by the self-heating effect in cyclically loaded FRP composite structures be reliably quantified and then be thoroughly removed by the implementation of coolant systems?

Every type of flow may come with its complexities. Among all three types of flows, it is merely the laminar flow that allows us not only to reliably analyze the fluid flow using computational fluid dynamics (CFD) software, but also to mathematically model and predict the location of the fluid particles with the passage of time. As the promising potential of this, the fatigue performance of a cyclically loaded PMC structure dominated by the self-heating phenomenon exposed to a laminar flow cooling system may be more reliably characterized, as opposed to transient or turbulent fluid [153].

Katunin and Wachla [87] reported that the lifetime of a cyclically loaded epoxy-based GFRP composite can be significantly extended by implementing airflow as forced air cooling. According to the authors’ experience, it should be noticed that although it might theoretically seem easy to have a laminar flow, providing such a flow and controlling such conditions for a prolonged period experimentally may not be as easy as expected. On the other hand, it may be difficult and complex to rationally predict the influence of both other cooling scenarios (transient and turbulent flows) on the value of transferred heat from the cyclically loaded FRP composite structures caused by the self-heating phenomenon. The most compelling reason justifying this can be explained by the vortices and eddies in flow, which are normally formed during both transient and turbulent fluid regimes, making the reliable predictability of fluid flow influence as a coolant system on fatigue performance of such a composite almost impossible. In order to logically formulate the caused self-heating effect under stationary or nonstationary condition in a cyclically loaded polymeric structure [61], a couple of scenarios can be taken into consideration.

***The first scenario*** is to assume the generated heat ($q_{gen}$) induced by the self-heating effect in a cyclically loaded polymeric structure is equal to the dissipated energy through convection, conduction and radiation in a loaded structure. One can obtain the ideal scenario due to the appearance of the thermodynamic equilibrium, wherein the effect of internal energy ($\Delta \dot{u}$) can be negligible (see Equation (6)). Therefore, the heat transfer equilibrium in Equation (6) for such a condition can be written in the following form [154]:

(17) $$q_{gen}=q_{dissip}\to \;\;\;q_{gen}=\frac{1}{V}\left(Q_{cond}+Q_{conv}+Q_{rad}\right),$$

It should be mentioned that since the heat transfer via radiation has an insignificant influence in such a condition, its effect can be eliminated due to the simplicity of equation systems [87]. Thus, Equation (17) can be rewritten as follows:

(18) $$\;\;\;q_{gen}\approx \frac{1}{V}\left(Q_{cond}+Q_{conv}\right),$$

This scenario may ideally provide us with the opportunity to completely remove the generated heat caused by the self-heating effect. Therefore, the structural degradation will be desirably prevented and merely limited to the influence of mechanical fatigue loading. To understand whether the overall generated heat has been removed via convection and conduction during fatigue testing, the surface temperature variations of a typical composite should be frequently monitored using the available techniques, including the use of IR thermographic cameras [74,87,89,155]. If there is no significant difference between the surface temperature before testing and the history temperature measurements extracted from IR thermographic data during fatigue testing, the stationary self-heating regime will appear for a long period until mechanical fatigue failure. Otherwise, the catastrophic nonstationary regime will appear.

***In the second scenario***, nonequilibrium conditions will occur between the amount of heat generation and the amount of heat dissipated via convection and conduction if there is a noticeable deviation between the specimen surface temperature during fatigue testing and the initial specimen surface temperature before testing. This is the appearance of a nonstationary regime due to the storage of a relatively high level of internal energy in the specimen. It would be, however, of utmost significance to estimate the difference between the resultant generated heat caused by the self-heating phenomenon and the heat removal via convection and conduction. In the case of heat transfer via conduction, the most influential parameter is thermal conductivity, which is discussed in the optimal materials design and selection section (see Section 4.2). Regarding the total heat removal via convection, what would be more significant is to compute the average convective heat transfer coefficient ($\bar{h}$) in the cyclically loaded PMC structure, which seems to be a time-demanding task. Although proposing empirical or semiempirical formulas for computing $\bar{h}$ on the entire surfaces of PMC structures seems to be a time-consuming task due to a number of uncertainties associated with the surface behavior (which can be rooted in the manufacturing techniques and the types of fibers, woven fibers and polymers used during the manufacturing process), a general technique may be implemented in order to acceptably estimate $\bar{h}$ on the entire surfaces of FRP composite structures regardless of the types of flow and fluid as follows [156]:

(1) The geometry of the fluid stream should be specified in advance (e.g., whether the fluid flows over a thin-walled FRP composite cylinder or a plate with a different thickness (i.e., ranging from FRP laminates to sandwich panel structures)).
(2) Since the Reynolds number is highly dependent on the boundary layer conditions, it should be correctly computed as a function of the characteristic length of the FRP composite structure and the kinematic viscosity and velocity of the flow.
(3) An appropriate reference temperature should be specified for the purpose of calculating the properties of the flow.
(4) In some cases, if a typical FRP composite structure has a complex geometry, it may be of utmost importance to understand whether a local convection coefficient or an average convection coefficient over the surface is needed for simulation.

In general, the average convection coefficient of such a PMC structure exposed to any external fluid flow can be predicted using the Nusselt number [156], expressed as follows:

(19) Nu=h¯ LK=C RemPrn,

where *C*, *m* and *n* are constants, varying based on how the fluid behaves, acting as laminar, transient or turbulent flow. Re and Pr are respectively called the Reynolds and Prandtl numbers.

The average Nusselt number based on the type of fluid flow over the entire isothermal FRP composite specimen can be denoted as follows:

For laminar flow:

(20) Nu=h¯ LK=0.664Re1/2Pr1/3;     Pr≥0.6,

For turbulent flow:

(21) Nu=h¯ LK=0.680Re1/2Pr1/3

and the average convection coefficient for both laminar and turbulent regimes can then be determined as follows:

(22) $$\bar{h}=\frac{\;NuK}{L}$$

where K is the thermal conductivity of the fluid around the specimen and L indicates the characteristic length of cyclically loaded flat FRP composite structure.

On the other hand, the Nusselt number for the thin-walled PMC structures, regardless of the type of cross-section and the type of flow around the thin-walled FRP composite structures, may be described as follows:

(23) Nu=h¯ DK=0.3+0.62Re1/2Pr1/31+0.4/Pr2/31/41+Re282,0005/84/5 ;    RePr≥0.2,

The average convective heat transfer coefficient for a thin-walled composite structure may then be computed as follows:

(24) $$\bar{h}=\frac{\;NuK}{D}$$

where D is the characteristic diameter of a typical cyclically loaded FRP composite structure. Thus, interestingly, having computed the average convection heat transfer coefficient, one could acceptably evaluate the value of heat removal by the forced flow on the entire surface of a cyclically loaded FRP composite structure (see Equation (11)).

As can be concluded from Equation (11), associated with the criticality of $\bar{h}$, as the average convection coefficient increases, the amount of generated heat caused by the self-heating phenomenon in such a structure that can be desirably removed increases. To put it differently, computing the average convective heat transfer coefficient allows us to search for some viable solutions in order to tremendously remove the generated heat caused by such a cyclic loading. Knowing this could be also important to estimate the difference between the generated heat induced by the self-heating phenomenon and the heat removed via convection and conduction.

**Figure 49:**
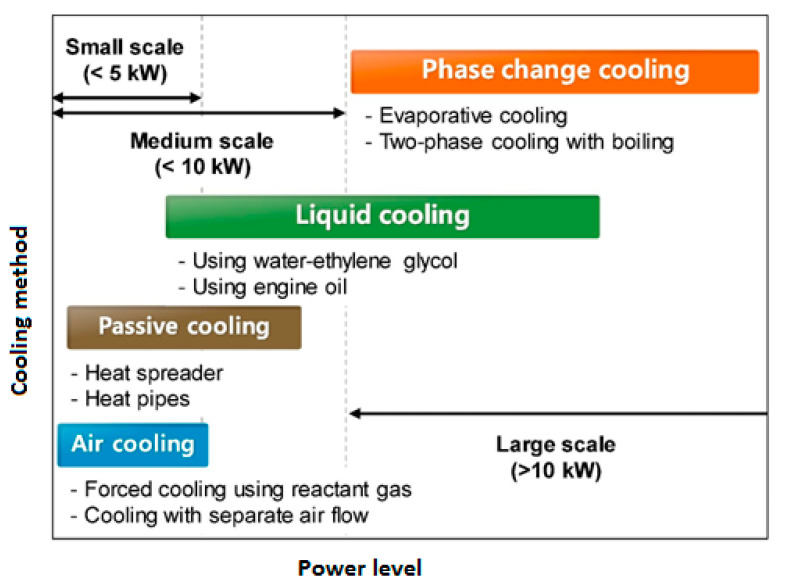
Different cooling techniques, adapted with permission from Ref. [157]. Copyright © 2019 Elsevier Ltd.

**Figure 50:**
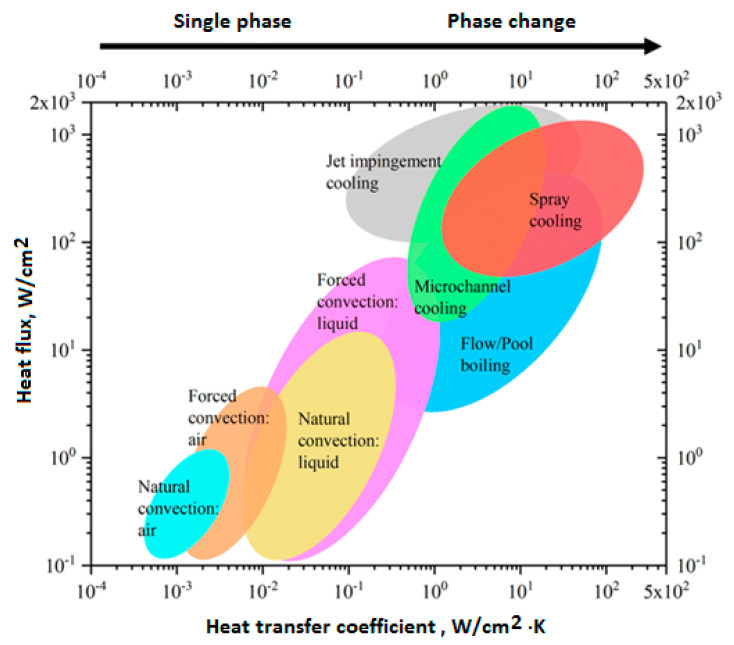
Classifying various cooling techniques according to the heat flux and heat transfer coefficient (adapted with permission from Ref. [158]. Copyright © 2022 Elsevier Ltd.) [159].

In general, numerous cooling methods based on enhancing $\bar{h}$ have been developed, namely airflow, passive, liquid and phase change cooling (see Figure 49 and Figure 50). Figure 50 generally categorizes numerous cooling methods of the heat-flux-removal capabilities per surface area via convection for the specific type of fluids with their heat transfer coefficients. It should be mentioned that although no report has been published to quantitively depict how such cooling techniques may positively increase $\bar{h}$, a number of reports have shown the noticeable overall heat flux removed via convection as the result of implementing the coolant systems [44], which is linearly proportional to $\bar{h}$ (see Equation (11)).

**Figure 51:**
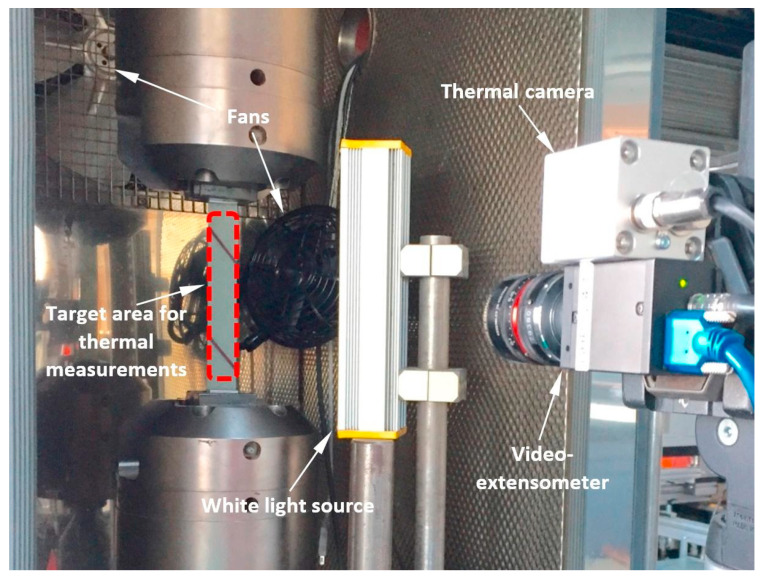
The experimental setup including coolant system for fatigue testing of angle-ply glass/epoxy structures, adapted with permission from Ref. [77]. Copyright © 2018 Elsevier Ltd.

Airflow cooling, for which either a fan or blower is mainly implemented, is the simplest cooling approach. Although this may be the most affordable cooling technique, due to the comparatively lower specific heat of air than water, this technique can be used when the rate of generated thermal heat on the surface of a PMC specimen is roughly small (normally less than 5 kW). For this purpose, Müller et al. [160] implemented air cooling (vortex tube) so as to avoid excessive warming of both angle-ply and cross-ply CFRP specimens in a damaged condition during the HCF regime. Additionally, Apinis [161] implemented forced air cooling when the specimen temperature was greater than 50 °C so as to prevent the self-heating effect resulting from the accelerated fatigue testing of carbon–carbon composites using high loading frequency. Katunin and Wachla [87] reported that the lifetime of a GFRP composite could be extended twice using airflow. Furthermore, Movahedi-Rad et al. [77] employed a couple of fans as the coolant system to circulate the air inside the chamber to remain at the constant temperature of 20 °C and cool down the angle-ply glass/epoxy composite laminates (see Figure 51). According to the obtained data, the values of 30 °C and 45 °C appeared as the maximum specimen surface temperatures at low/intermediate stress levels and higher stress levels before failure, respectively.

**Figure 52:**
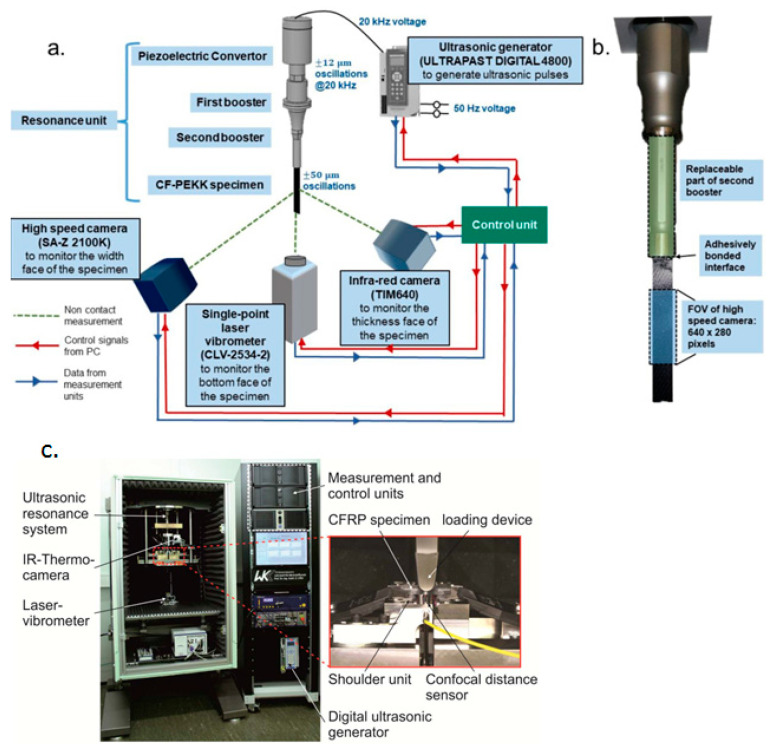
(**a**) Schematic experimental setup of the developed ultrasonic testing facility for axial cyclic loading of PMCs at a frequency of 20 kHz; (**b**) connecting the specimen to the system using the high-speed camera [29]; (**c**) ultrasonic testing setup for 3-point fatigue bending of CFRP (adapted with permission from Ref. [31]. Copyright © 2016 Elsevier Ltd.) [48].

**Figure 53:**
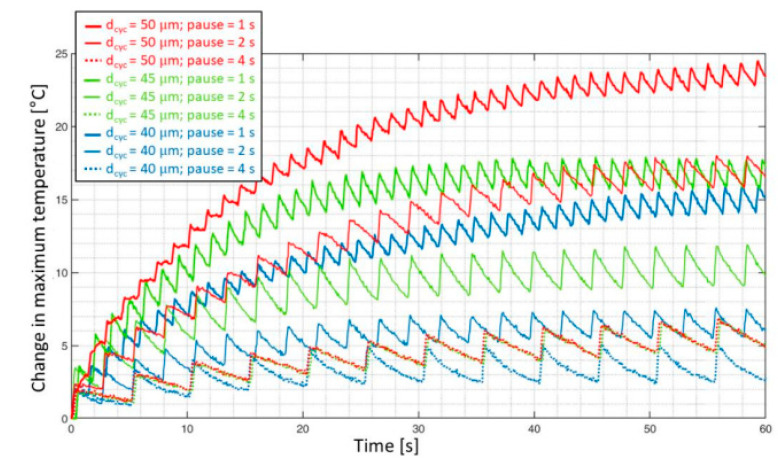
The influence of various load amplitudes and pause times on the surface temperature of a CFRP specimen during cyclic loading at the frequency of 20 kHz during the first minute [65].

**Figure 54:**
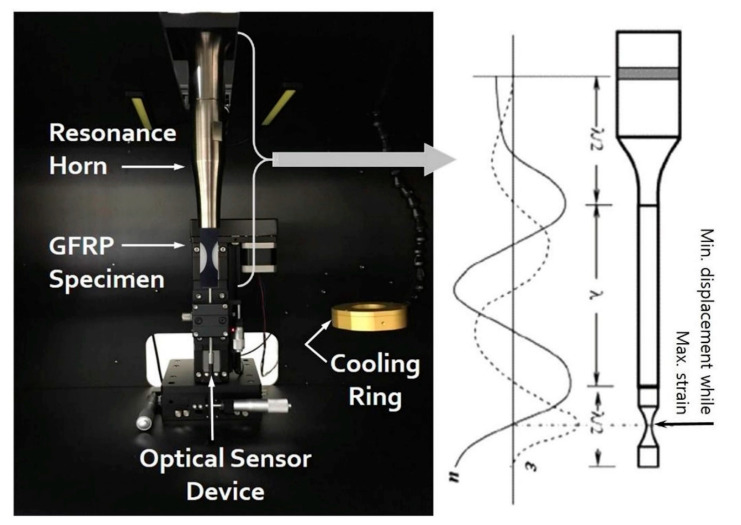
The experimental setup for ultrasonic fatigue testing of GFRP specimens and schematic illustration of resonance test, adapted with permission from Ref. [163]. Copyright © 2019 Elsevier Ltd.

In addition, the pulse–pause air cooling technique has been mainly implemented for ultrasonic fatigue experiments [29,65,123,162]. Premanand and Balle [29] developed an ultrasonic fatigue test setup using the reverse piezoelectric effect to obtain a cyclic mechanical oscillation of 20 kHz. A schematic representation of the developed facility and online-monitoring devices along with their model names is shown in Figure 52. This system involves an ultrasonic generator, a piezoelectric convertor and two boosters. The control unit had been implemented to handle the ultrasonic oscillation parameters from the generator inside the LABVIEW environment. For the purpose of preventing the appearance of the self-heating effect, the entire fatigue experiments were conducted in an unchanged pulse–pause ratio with 200 ms of ultrasonic pulse and 2000 ms of pause. While this constant pulse–pause ratio was applied, dry compressed air was employed as the coolant agent to limit the surface temperature of the specimen to 30 °C. Additionally, Flore et al. [30] used an unchanged pulse–pause ratio including 100 ms of pulse and 2000 ms of pause during fully reversed ultrasonic cyclic tension testing of a GFRP composite for the purpose of limiting temperature to 25 °C on the specimen surface. In another study, Premanand and Balle [65], as opposed to their previous study in which pause time was assumed to be constant with the value of 2000 ms [29], investigated CFRP specimens at three different cyclic amplitudes (40 μm, 45 μm and 50 μm) and three pause time durations (1000 ms, 2000 ms and 4000 ms) using the similar ultrasonic testing setup for three-point fatigue bending of CFRPs developed by Backe et al. [48] (see Figure 52c) to prevent premature thermal failure of specimens induced by the self-heating effect (see Figure 53). As can be seen in Figure 53, the surface temperature of the CFRP specimen was significantly affected by the value of pause time and cyclic amplitude loading. The longer pause duration advantageously resulted in lower temperature growth. For example, the temperature growth of almost 5 °C at the end of the shown time period was measured as the result of applying a cyclic oscillation of 50 μm and a pause time of 4 s, which was approximately one-fifth of the value obtained when 1 s pause was used. In addition, with the increase in the loading amplitude applied to the specimen, the temperature growth measured increased. Lee et al. [163] implemented a Peltier ring-type cooler, also known as a thermoelectric-based air cooler [164], in order to positively generate a high cooling effect for polyamide 66/glass fiber composite structures (see Figure 54). They used a constant pulse–pause ratio with a pulse time of 300 ms and a pause time of 3000 ms to avoid specimen heating.

**Figure 55:**
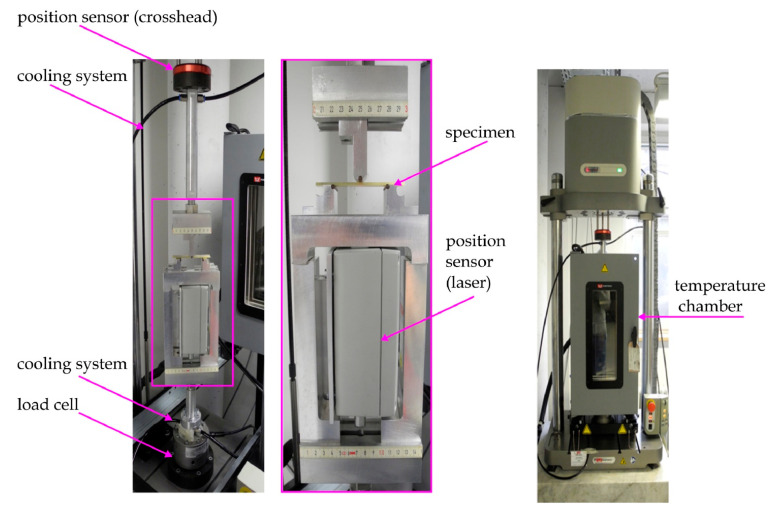
The experimental setup for characterizing DMTA using a water-based coolant system [169].

**Figure 56:**
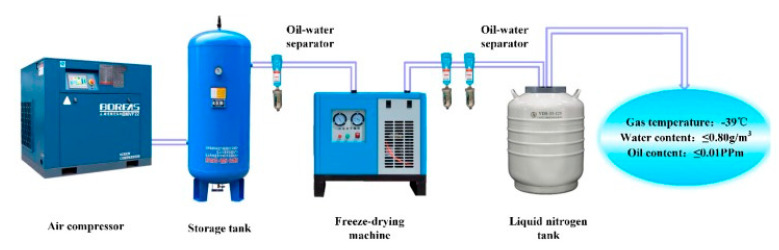
An exemplary illustration of a liquid nitrogen cooling system [170].

The generated heat caused by the self-heating effect can be also removed via liquid cooling technique. As can be seen in Figure 49 and Figure 50, the level of heat removal resulting from the use of this cooling method can be comparatively higher than that resulting from the use of air cooling. The main causative root justifying this is the relatively greater convective heat transfer coefficient of liquids ($\bar{h}$) as opposed to air. Implementing liquids as the coolant systems may relatively result in more uniform temperature distribution on the specimen surface as compared to air due to their higher $\bar{h}$ [165]. According to the recommendations, the implementation of liquid-based coolant systems may be taken into consideration when the rate of generated thermal energy is higher than 5 kW since the developed air cooling systems may probably be unable to remove such a large amount of heat flux via convention [166]. As a general rule, high thermal capacity, low viscosity, chemical inertness, anticorrosion properties, nontoxicity and affordable price are the main factors in the selection of an appropriate coolant system [167]. Water has been the primary candidate among all liquids due to its high thermal capacity and low price. For this purpose, Just et al. [168] used a water-cooled base plate attached underneath the VHCF-tested cross-ply CFRP specimen so as to limit the generated heat flux induced by shaker. Additionally, Trauth et al. [169] implemented a water cooling system (see Figure 55) to study the effect of temperature on cyclically loaded continuous–discontinuous sheet molding composites (SMCs) by providing an integrated temperature chamber ranging from −100 °C to 350 °C for dynamic mechanical thermal analysis (DMTA). In another study, Cui et al. [170] developed a liquid nitrogen coolant system (see Figure 56) to limit the surface temperature of a CFRP specimen to lower than 40 °C during three-point bending fatigue testing in the VHCF regime. As shown in Figure 56, during the test, they inserted a metal tube into a liquid nitrogen tank; the liquid nitrogen absorbed heat, and the value of fluid temperature dropped remarkably with the passage of time, cooling the fluid to −39 °C. They justified the implementation of liquid nitrogen due to observing a remarkable temperature rise up to 106 °C in only 7 s on the surface of a CFRP specimen subjected to high loading frequency. This means that implementing compressed air cooling during ultrasonic loading of CFRP could not guarantee the normal conditions of a test (i.e., preventing the self-heating effect). As mentioned above (see the Section 4.1.1), Bartkowiak et al. [82] implemented liquid nitrogen (N_2_) as the coolant system for the purpose of providing a temperature of −20 °C for fatigue testing. In another study, Charalambous et al. [79] used liquid carbon dioxide as a coolant agent in order to limit the negative influence of the self-heating effect during cyclic fatigue testing of CFRP specimens by obtaining temperatures up to −50 °C.

The use of liquids with relatively high thermal conductivity (*K*) which may, similar to $\bar{h}$, act as a catalyst in removing heat generation on the surface of a PMC structure under cyclic loading experiments has been suggested. Nowadays, nanofluids, which are defined as the colloidal suspensions of nanoscale particles in a base fluid such as silicon dioxide (SiO_2_), carbon nanotube (CNT), graphene (GN) and alumina (Al_2_O_3_) nanoparticles in water, have been gaining great attention due to possessing a capability for enhancing the *K* of such a fluid [171,172,173]. This has advantageously resulted in the need for a relatively smaller coolant system, compared to traditional liquid cooling systems due to lower *K*. Although such techniques have been developed, they have not been implemented during fatigue testing of PMC structures. Therefore, implementing such nanoparticles in water or other liquid fluids can be taken into consideration as a coolant system during accelerated fatigue tests with high loading frequency in future trials.

**Figure 57:**
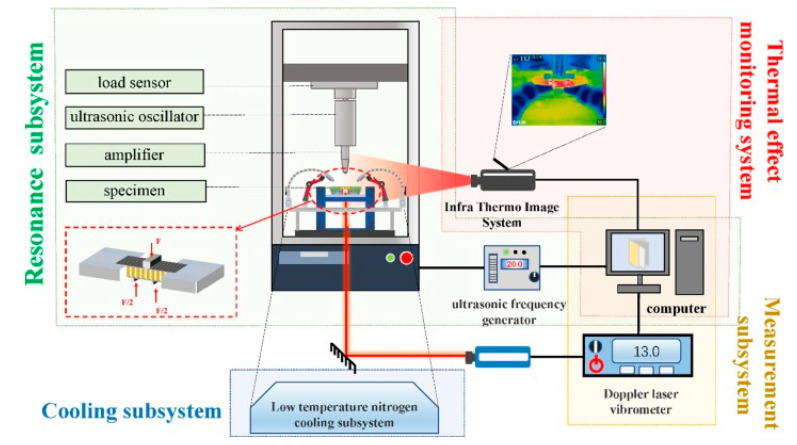
Schematic illustration of ultrasonic fatigue testing system, adapted with permission from Ref. [44]. Copyright © 2022 Elsevier Ltd.

**Figure 58:**
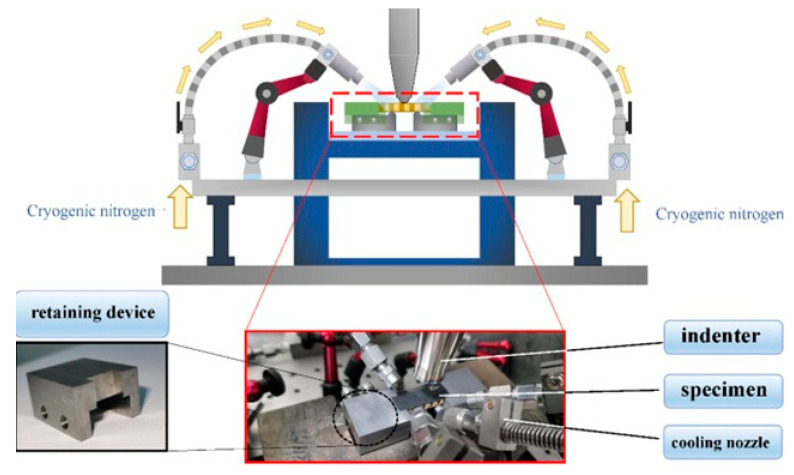
Schematic illustration of supporting device and cryogenic nitrogen as a coolant agent, adapted with permission from Ref. [44]. Copyright © 2022 Elsevier Ltd.

In another study, Wang et al. [44] investigated the HCF and VHCF properties of a CFRP-aramid honeycomb sandwich structure under three-point bending ultrasonic loading with the applied loading frequency ranging from 18.5 to 20.5 kHz. They implemented cryogenic nitrogen and intermittent loading (i.e., pulse–pause technique) simultaneously to cool down the specimen for the purpose of limiting the self-heating temperature to below 40 °C (see Figure 57). The schematic diagram of the supporting device and cryogenic nitrogen as a coolant agent is shown in Figure 58. It should be mentioned that none of the authors of the previous studies reported the exact information about the rate of heat removal by the applied coolant systems during fatigue testing of PMC specimens, which is of key interest in conducting comprehensive studies in order to characterize the pros and cons of the available cooling techniques in terms of their simplicity, cost, etc.

Another cooling technique is passive cooling or edge cooling, which mainly implements heat spreaders with microchannel-based heat sinks [174,175]. High heat spreaders involving copper, aluminum, silver and silica, due to their high thermal conductivities, can be the prime candidates for implementation as thermal management for PMC specimens subjected to fatigue testing, which are also analyzed in the section on materials design and selection. For this purpose, Bahiraei et al. [174] implemented hybrid graphene–silver nanoparticles in a nanofluid base via a microchannel heat sink in a laminar flow with a Reynolds number of 100. They reported that adding 0.1% graphene nanoplatelets to the base fluid increased the average heat coefficient by 17%, which is proportional to heat removal by convection.

**Figure 59:**
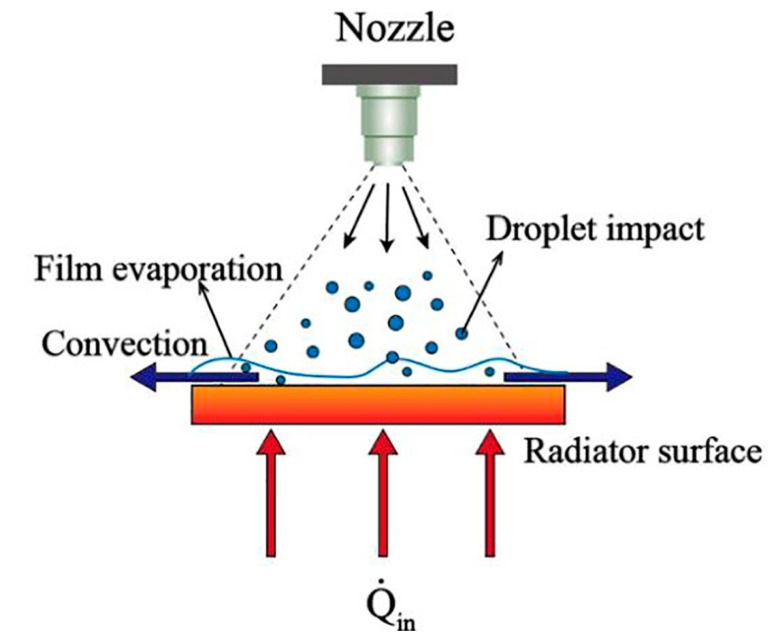
A schematic demonstration of spray cooling process, adapted with permission from Ref. [158]. Copyright © 2022 Elsevier Ltd.

Another viable cooling technique is phase change cooling, for which coolant pumps are advantageously not required [176]. It may be interesting to know that the phase change technique implements significantly greater latent heat during the two-phase heat removal mechanism, as opposed to air and water cooling. For instance, the latent heat of water is 2250 kJ/kg, which is more than 500 times greater in comparison to the sensible heat absorbed by liquid water with a temperature increase of 1 °C, meaning a comparatively lower coolant flow rate is required [166]. Among phase change cooling techniques, including microchannel flow boiling, heat pipe cooling and spray cooling, it is spray cooling that has been receiving great attention due to its excellent cooling capacity, uniformity, fluid utilization efficiency and system flexibility [159]. As can be seen from Figure 59, a spray of droplets generated by pressure/gas atomized nozzles is forced to impinge onto the targeted heating surface (i.e., the surface of the composite specimen), cooling it efficiently with a phase change of the coolant. Spray cooling can be implemented for both steady-state and transient cooling conditions. Steady-state cooling is employed when the specimen surface temperature is kept below the critical heat flux limit, while transient spray cooling is associated with the quenching of a target with a high initial temperature where the target is gradually cooled down. Since the initial specimen temperature during the fatigue test is often equal to RT, steady-state spray cooling can be efficiently implemented. For example, Putnam et al. [177] implemented steady-state spray cooling based on water microdroplet evaporation. They reported that when the heat flux reaches the critical heat flux in the heating-up direction, the continuous liquid film may degrade to isolated microdroplets, resulting in a high heat flux (${1000\;W/\mathrm{cm}}^{2}$) and convective heat transfer coefficient (${60\;W/\mathrm{cm}}^{2}K$).

**Figure 60:**
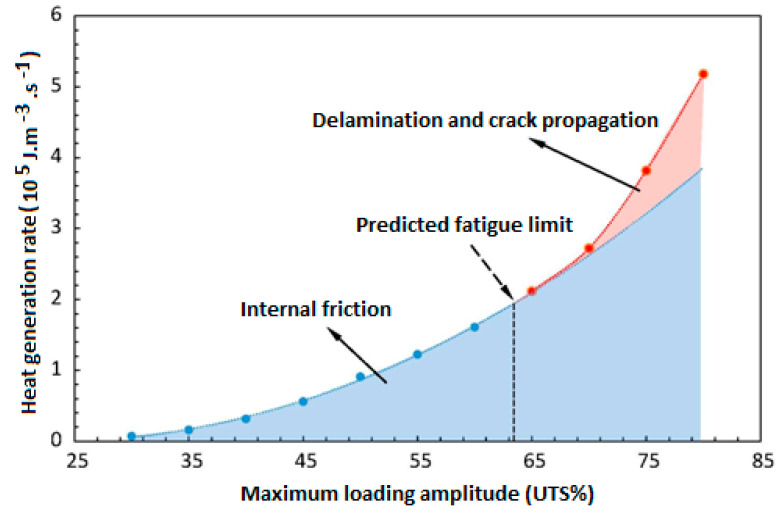
The exemplary illustration of heat generation rates depending on loading amplitude, adapted with permission from Ref. [89]. Copyright © 2020 Elsevier Ltd.

As a recommendation, it may be more practical if the researchers quantify and report the heat flux during fatigue testing to rationally justify the reason behind selecting a specific cooling technique in future studies. As a result of having information about the generated heat flux rate induced by the self-heating effect under cyclic tests, reliable classification and standardization according to the material selection, the type of fatigue loading and the specimen geometry can be desirably carried out. This will provide us with the opportunity to select the most appropriate (optimal) coolant system for a specific cyclically loaded PMC structure. For this purpose, Huang et al. [89] numerically quantified the heat generation per unit volume during fatigue testing for CFRP laminates. They illustrated how the rate of generated heat in a PMC may significantly vary as the direct consequence of the viscoelastic behavior of the polymer, and damage propagation simultaneously, during fatigue testing [89] (see Figure 60). As they reported, the heat generation rates under different stress levels can be explained by internal friction and damage mechanisms (e.g., delamination and crack propagation). The heat generation rate resulting from internal friction and delamination/crack propagation is separately shown in Figure 60, wherein the blue region depicts the heat generated caused by internal friction (i.e., the self-heating effect) and the red area reveals the generated heat caused by damage. Those two factors are proportional to the applied stress magnitude. As the stress level applied to such a PMC specimen increased, the measured heat generation increased. As they reported, the heat generation rate caused by damage noticeably increased when the stress levels exceeded the fatigue limit. The primary reason behind the heat generation associated with internal friction can be sought in recoverable microstructure motion (e.g., the interface between fiber and polymer matrix and the interlaminar interfaces between two adjacent layers).

**Figure 61:**
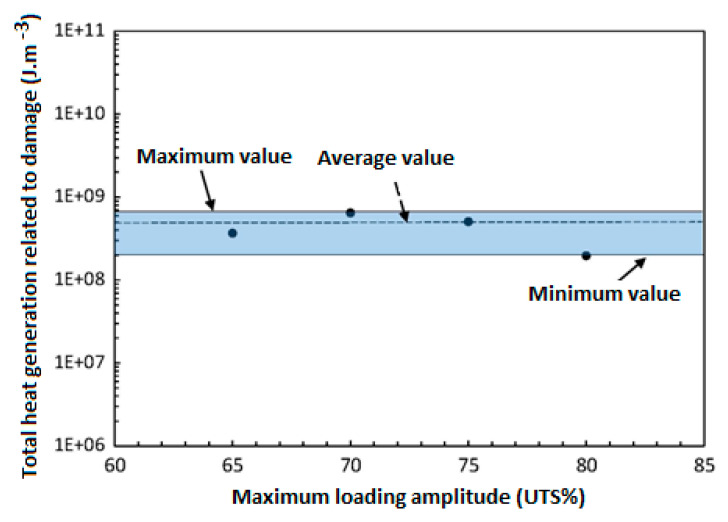
The exemplary heat generation plot for CFRP composite induced by damage over the entire fatigue life, adapted with permission from Ref. [89]. Copyright © 2020 Elsevier Ltd.

Figure 61 reveals the total heat generation per unit volume for a CFRP laminated composite caused by damage during fatigue life under different stress levels based on numerical data. As can be concluded, the average value of heat generation after the fatigue limit is exceeded is dominated by other heat sources and seems to be less dependent on applied stress levels. This means that for quantifying the total heat generation by damage, a fatigue test at one specific stress level would be sufficient.

In order to appropriately implement a cooling method, the entire mechanical energy dissipation associated with both internal friction and damage should be reported and quantified, not merely the heat generation caused by damage, which can be taken into consideration for the future studies.

### 4.2. Limiting Structural Degradation Caused by Self-Heating via Optimal Materials Design and Selection

The effects of loading conditions on the level of heat generation induced by fatigue loading were discussed for LCF, HCF and VHCF regimes. As discussed, depending on the loading conditions, a high level of temperature may appear on the surface of a composite structure during fatigue testing in all mentioned fatigue regimes, which may accelerate structural degradation in such a structure. Materials design and selection, as well as implementing cooling scenarios, can effectively limit the structural degradation of PMC structures resulting from the self-heating effect. Thus, if materials are appropriately implemented (e.g., using materials with high thermal conductivity (*K*)) during the manufacturing process of PMC structures, structural degradation might be avoided or at least minimized. Otherwise, when the manufacturing processes of PMC structures have been thoroughly completed, it may be indeed unjustifiable due to being a time-consuming and costly process or even impossible to change and replace the used materials with other ones in order to enhance the *K* of such PMC structures. Thus, in such conditions when the composite structures have been produced, using different cooling scenarios (which were broadly discussed in the previous section) may be the only option for preventing intensive structural degradation with the presence of the self-heating phenomenon under fatigue loading.

*K* can favorably play a key role in limiting the intensive structural degradation of PMC structures. Enhancing *K* of PMCs will lead to increasing heat dissipation via conduction. This can minimize the stored internal energy, resulting in a relatively low temperature on a composite surface. This prevents the occurrence of premature thermal fatigue failure in such a structure, extending residual fatigue lifetime or reducing the structural degradation of the PMC structures. The most rational justification behind this can be rooted in how increasing the value of *K* functions, transferring the heat caused by the self-heating phenomenon with a higher rate from the surface of the PMC structure into the surrounding environment via convection and consequently cooling down a PMC specimen surface temperature. Therefore, implementation of PMC materials with higher *K* values is of key interest to tackle the issue of the self-heating effect resulting from high-amplitude vibrations or fatigue loading. In general, to characterize how the self-heating phenomenon of a PMC can be advantageously minimized through utilization of thermally conductive materials, the influence of numerous parameters which are analyzed in detail in the subsequent subsections, should be taken into consideration.

#### 4.2.1. Role of PMC Specimen Thickness

**Figure 62:**
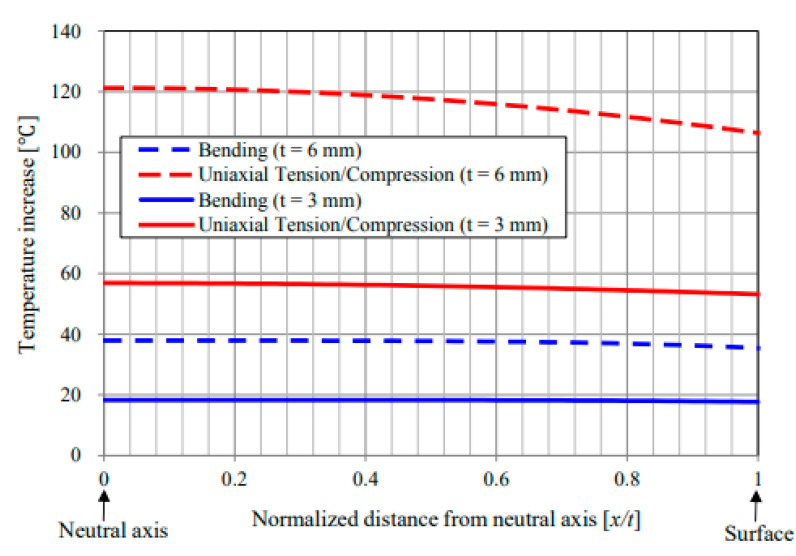
The cross-sectional temperature distribution in laminated GFRP composite specimens with thicknesses of 3 and 6 mm estimated under the assumption of one-dimensional heat transfer [178,183].

In the case of optimal materials design and selection, there may be a number of causative factors contributing to improving the *K* values of PMC structures to avoid the intensive structural degradation of such PMCs, one of which may be the specimen thickness. For this purpose, Ueki [178] provided analytical relations for the determination of the temperature growth during a fatigue test and the temperature distribution through the laminated specimen thicknesses with values of 3 mm and 6 mm. As a result of increasing the specimen thickness, the temperature rose significantly by almost 100%, ranging from 20 °C to 40 °C in bending mode, and varying from just below 60 °C to 120 °C in uniaxial tension/compression mode with loading conditions of *f* = 200 Hz, σ = 100 MPa and *R* = −1 (see Figure 62). In the case of axial loading, when the thickness was 6 mm, the temperature value dropped by about 15 °C, and the midplane of the specimen experienced the highest value of temperature. To put it differently, in the case of one-dimensional heat transfer under unchanged loading conditions, the thinner a PMC specimen is, the more uniform the temperature distribution in the cross-sectional direction and the lower the temperature value can be. For this reason, Mandell et al. [179] used a thin laminated composite with a thickness of 1.5 mm for fatigue testing. Furthermore, Hosoi et al. [180,181,182] implemented thin laminated specimens with a thickness of about 1 mm for fatigue testing in order to limit the self-heating effect through the thickness direction. According to the ASTM D3039 standard [13], unidirectional laminated composites with a thickness of 1 mm can be tested under axial fatigue loading.

#### 4.2.2. Role of Polymer Matrix

**Figure 63:**
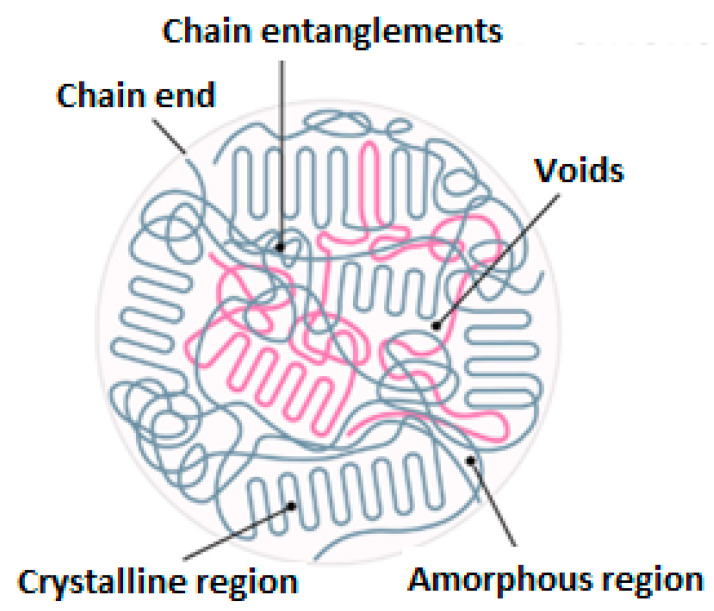
Demonstration of amorphous and crystalline polymer–matrix structures, adapted with permission from Ref. [192]. Copyright © 2021 Elsevier Ltd.

**Figure 64:**
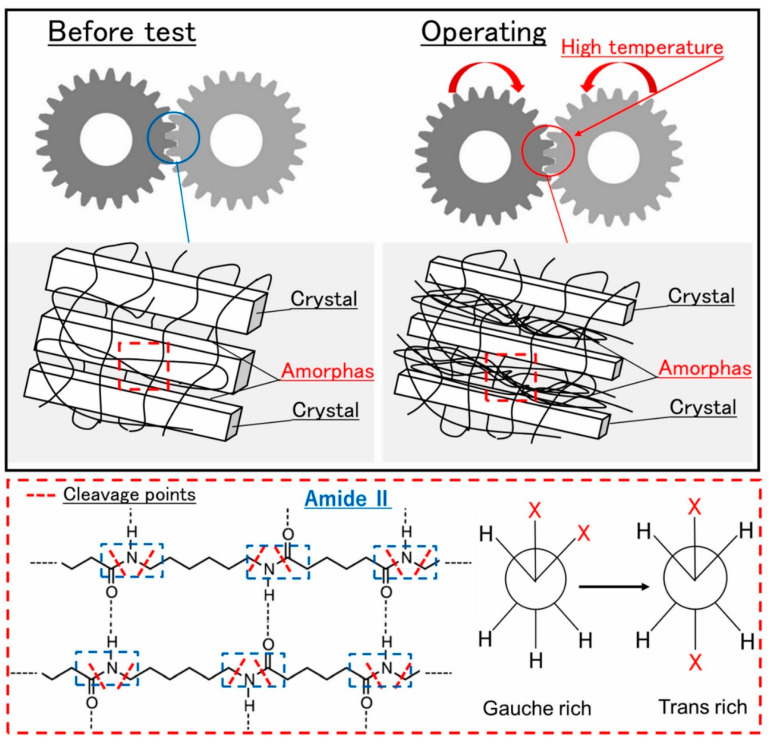
Schematic representation of the structural change of a PA 66 gear induced by self-heating effect during fatigue loading [193].

**Figure 65:**
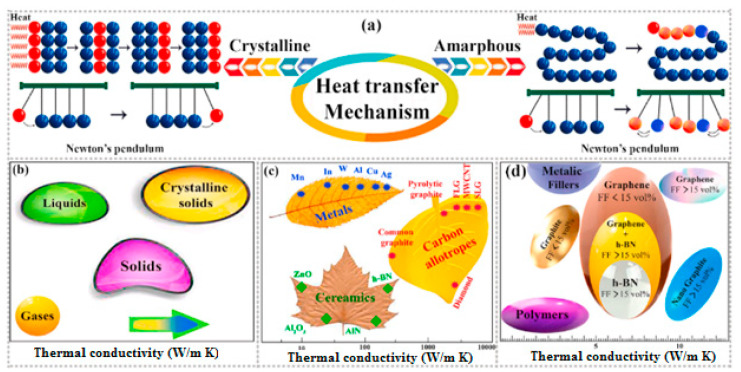
Schematic demonstration of (**a**) heat transfer mechanism in crystalline and amorphous polymers, (**b**) *K* levels in different phases of materials, (**c**) classifying common thermally conductive solid materials for various filler materials and (**d**) categorizing various filler-reinforced PMCs regarding their thermal conductivities, adapted with permission from Ref. [189]. Copyright © 2022 Elsevier Ltd.

It has been suggested that the self-heating effect can be controlled using appropriate polymers. Table 1 summarizes the *K* values for the most common thermoset and thermoplastic polymers. Although polymers may have manifold merits, their *K* values are relatively low, ranging from just below 0.15 up to roughly 0.5 for commonly used polymers [184,185,186,187,188,189,190,191]. This means that polymers may not be considered the prime candidates for improving the *K* values of PMC structures. It is worth mentioning that the primary causative factor contributing to the difference between the *K* values of such polymers can be explained by the polymer structures (i.e., level of crystallinity). A higher degree of crystallinity may lead to a greater *K* value. According to Table 1, a good illustration of this can be high-density polyethylene (HDPE), which offers a greater *K* value due to possessing a higher degree of crystallinity, as opposed to low-density polyethylene (LDPE). Figure 31 and Figure 63 depict both amorphous and crystalline polymer structures, wherein amorphous polymers consist of chain entanglements, chain ends, crystal–amorphous interfaces and voids [192], as the direct consequence of which the heat transfer mechanism along chains may be frequently interrupted. As an example, Figure 64 schematically reveals the influence of the self-heating phenomenon on the structural variation of a PA 66 gear during fatigue operation [193]. Additionally, the heat transfer mechanism in polymers with crystalline and amorphous zones is schematically shown in Figure 65a [189].

#### 4.2.3. Type of Fillers

As opposed to polymers which possess relatively low range of *K*, fillers offer a comparatively wider range of *K* values. Such materials can be categorized by their family groups (e.g., carbon allotropes, ceramics, metals and polymeric fibers). Table 2 reveals the *K* values of fillers which are commonly implemented in a variety of engineering applications. According to provided data in Table 2, among all thermally conductive filler candidates, leading the pack would be the carbon-based allotropes consisting of carbon nanotubes (CNTs), graphene, graphite and carbon fibers, offering ultrahigh *K* values of up to 6000 W/m K. The potential carbon-based fillers offer *K* values approximately 14 times greater than those of common metals and roughly 6000 times higher than that of glass fiber. These are followed by commonly used ceramics and metals, which also offer relatively high *K* values, ranging from 50 W/m K for steel to greater than 1000 W/m K for diamond. Polymer-based fibers such as Kevlar (aramid) as well as basalt fiber trail the pack, possessing comparatively unimportant *K* values of 0.04 W/m K or below, which are even lower than the *K* values of common engineering polymers. This means that such fibers do not have the capability to be implemented for improving the overall *K* of a PMC structure. Figure 65b–d schematically classify different materials in terms of their *K* values [189]. Therefore, carbon-based fillers can be selected as the prime candidates for enhancing the *K* of PMC structures, as discussed in the next section.

#### 4.2.4. Role of Fillers in Overall *K* of PMC Structures

One of the viable strategies for the purpose of enhancing the *K* values of PMCs is to implement ultrahigh-thermal-conductivity micro- or nanocarbon allotropes involving carbon nanotubes (CNTs) [203], graphene [204,205] and carbon fibers [206,207], as well as boron nitride (BN) platelets/nanosheets [188,208,209,210,211,212,213], silicon carbide (SiC) [214,215,216], silicon nitride (Si_3_N_4_) [217], gold (Au) [218], copper (Cu) [219,220,221,222], silver (Ag) [223,224,225], aluminum nitride (AlN) [226], aluminum oxide (Al_2_O_3_) [227] and hybrid fillers [188,228,229,230] during the fabrication of PMC structures. To predict how the overall *K* value of a typical PMC is influenced by adding fillers into the polymer matrix, the implementation of the rule of mixture equations is indeed impractical due to providing a huge mismatch, as opposed to experimental results [189,192]. The primary causative factors contributing to this mismatch between extracting *K* values using the rule of mixture and experiments can be sought in the (i) geometry of the filler, (ii) thickness of the surface coating and film, (iii) orientation of the filler, (iv) interface between the polymer matrix and filler and (v) loading fraction of the filler.

In the case of filler geometry, the size and shape of fillers play a key role in the *K* of PMCs. This can be explained by the aspect ratio of a filler, which is defined as the ratio of length to diameter for cylindrical fillers (e.g., CNTs) and the ratio of length to thickness for platelet fillers (e.g., graphene nanoplatelets (GNPs)). A greater aspect ratio may result in a relatively lower mismatch [231,232,233,234]. Additionally, the diameter plays a key role in determining the *K* of composites reinforced with spherical particles. PMCs filled with spherical fillers normally offer comparatively lower *K* due to a lack of perfect connection with each other, limiting the movement of phonons. In the case of fiber orientation, fillers mainly possess anisotropy of properties and heterogeneity, influencing the heat flow efficiency. Therefore, implementing fillers with controlled orientation using hot pressing [235], magnetic alignment [236] or electrospinning [237] can offer relatively high *K* values. For example, Pan et al. [235] reported a 350% increase in the *K* of epoxy/Al_2_O_3_ composite using the hot pressing orientation technique.

The loading fraction of fillers is another key factor in increasing or decreasing the *K* value of PMCs. From the experimental point of view for low and moderate filler fractions, Chudek et al. [238] mixed graphene nanoplatelets (GNPs) with polylactic acid (PLA) randomly using the hot pressing technique. They reported the *K* value of 1.72 W/m K for a GNP/PLA composite as the result of adding 15 wt.% GNPs, increasing the *K* of the host PLA polymer matrix by 406%. In addition, Raza et al. [239,240] reported the *K* values of 1.909 W/m K and 2.2 W/m K for GNP/silicone composites with a filler fraction of 20 wt.% fabricated by mechanical mixing and three-roll milling, respectively. Moreover, Wang et al. [241] illustrated the in-plane *K* value of 15.8 W/m K for GNP-nylon composites fabricated using vacuum-assisted filtration (VAF) and compression molding (CM) by adding 11.8 wt.% graphene, which was 62.2 times greater than that of the pure polymer. In another study, Wang et al. [203] fabricated polyoxymethylene (POM)/multiwalled carbon nanotube (MWCNT) and POM/GNP composites (PMCNT and PMGNP, respectively). They exhibited the through-plane *K* values of 1.95 W/m K and 4.24 W/m K for the PMCNT40 (with 40 wt.% MWCNT) and PMGNP48 (with 48 wt.% GNP), respectively, which were approximately 4.6 and 11.1 times greater than those of neat POM in the through-plane direction, respectively. Nevertheless, the in-plane *K* values of the PMCNT40 and PMGNP48 composites were 4.17 W/m K and 36.35 W/m K, respectively. The in-plane *K* of PMGNP48 composite was ca. 13 times greater than that of pure POM. In addition, Wang et al. [242] implemented single crystalline copper nanowires with large aspect ratios as fillers in an acrylate polymer matrix. They measured the *K* of 2.46 W/m K at an ultralow loading fraction, roughly 0.9 vol.%, increasing it by 13.5 times compared with the host polymer matrix. In another study, Balachander et al. [218] developed a highly conductive polydimethylsiloxane nanocomposite filled with a low volume fraction of gold nanowires. They reported the *K* value of 5 W/m K using just below 3 vol.% gold nanowire fillers, leading to a 30-fold increase in the *K* of polydimethylsiloxane (PDMS).

It should be mentioned that the upper limit of graphene loading fraction in PMCs is normally 20 vol.% to avoid electrical percolation [243], obtaining a moderate *K* value of 20 W/m K or below. However, there may be some exceptions. On the other hand, BNNS and fluorinated graphene (F-graphene) fillers as the prime candidates can be even implemented for manufacturing composites with high loading filler fractions. For this purpose, Wang and Wu [244] used F-graphene filler, exfoliated from commercial fluorinated graphite, with loading fraction of 93 wt.%. They reported the in-plane *K* value of 61.3 W/m K. In another study, Wu et al. [245] implemented BNNS with loading fraction 90 wt.% in a poly diallyl dimethyl ammonium chloride (PDDA) matrix. They showed a high in-plane *K* with the value just above 200 W/m K and a relatively low out-of-plane *K* of 1.0 W/m K.

According to the obtained data for *K* ranges of isotropic and anisotropic (in-plane direction) PMCs and *K* enhancement ($K_{c}/K_{m}$) reviewed in [243], the vast majority of PMCs possess *K* values higher than 1 W/m K. Nonetheless, a minority of them have values greater than 20 W/m K (see Figure 6 in [243]), which mainly happen at moderate and high filler loading fractions (more than 40 vol%). Additionally, *K* was reported to be increased compared to pure polymer matrix ($K_{c}/K_{m}$) by 10 times for most composites, 100 times for a minority of composites and by roughly 4200 times for nanofibrillated cellulose (NFC)/BNNS.

Furthermore, the mathematical models developed for quantifying the *K* of PMCs filled with particles have been reviewed in [192,246]. The proposed models are briefly presented in Table 3, wherein $K_{eff},K_{f}$ and $K_{m}$ denote the effective thermal conductivity of the filler-embedded polymer matrix, the thermal conductivity of the filler and the thermal conductivity of the host polymer matrix, respectively; φ, d and $R_{k}$ indicate the filler volume fraction, the filler diameter and the thermal interface resistance between the particle and the host polymer matrix, respectively; and $K_{CNT}$, L and t represent the K of a carbon nanotube, the length of a CNT and the thickness of the laminated composite, respectively. As can be concluded, the *K* of PMC is considerably lower than that of fillers (see Table 2). This significant difference can be explained by the high thermal resistance ($R_{k}$) at the filler–host interfaces and other factors, which are discussed later. As a recommendation, the results obtained from experiments can be compared with such data extracted from the proposed mathematical models in Table 3 in further studies by researchers. For this purpose, Wang et al. [247] illustrated that both the Maxwell and Nielsen models can accurately predict the *K* of PMCs when the filler volume fraction is lower than 10 vol.%, while the Nielsen model is in good agreement with experiments when the filler volume fraction is above 10%.

According to the previous studies reviewed in [243], as the amount of filler utilized increases, up to a specific limit depending on the type and geometry of fillers and polymers, the *K* value that can be achieved increases. This limitation for adding filler up to a certain level can be explained by agglomeration and/or saturation of fillers in the polymer matrix [255], the formation of voids and poor dispersion [256,257], etc.

#### 4.2.5. Influence of Hybrid Fillers in Overall *K* of PMC Structures

One of the viable solutions in order to achieve higher *K* values is to fabricate and implement hybrid fillers in polymeric materials; for targeting highly conductive hybrid composites, such hybrid fillers must possess adequate functionalities so as to make an interconnected structure with each other using chemical vapor deposition (CVD) (see for example [258]). For this objective, Bozlar et al. [259] illustrated a 1.3 times increase in *K* value of a host Al_2_O_3_-epoxy composite as the result of adding a low MWCNT mass fraction of 0.15 wt.%, obtaining the *K* value of 0.39 W/m K. In addition, Yu et al. [229] implemented hybrid fillers of single-walled carbon nanotubes (SWNTs) and graphite nanoplatelets (GNPs), with the hybrid loading mass fraction of 10%, consisting of 2.5 wt.% SWNTs and 7.5 wt.% GNPs. They reported a *K* value of 1.75 W/m K, which was roughly 5 times greater than that of pure epoxy. Teng et al. [260] studied the synergistic influence of combining MWCNTs and boron nitride (BN) flakes on the *K* of epoxy-based composites. In order to form the covalent bonds between the epoxy and fillers, the surfaces of such fillers were functionalized, leading to a reduction in the thermal interfacial resistance ($R_{k}$). They illustrated a *K* value of 1.913 W/m K as the result of adding 30 vol.% modified BN and 1 vol.% functionalized MWCNTs, obtaining a 7.43-fold increase in such a figure for pure epoxy (0.2267 W/m K). Im et al. [261] investigated the *K* behavior of a graphene oxide (GO)–MWCNT hybrid/epoxy composite. As they showed, adding merely 0.36 wt.% of MWCNT into 50 GO-epoxy composites resulted in the highest enhancement ratio (about 140%) relative to the GO/epoxy composite, which was explained by the formation of 3D heat conduction paths due to the addition of MWCNTs. Yu et al. [262] investigated how the *K* of an Al_2_O_3_-silicon composite is positively influenced by graphene. They demonstrated that adding 1 wt.% of graphene increased the *K* of Al_2_O_3_-silicon from 2.70 W/m K to 3.45 W/m K for a graphene-Al_2_O_3_-silicon composite. Sharma et al. [263] investigated how the *K* of SWCNT-metallic glass (MG) composites is influenced by the variation in the SWCNT volume fraction. They reported the values of 1.52 W/m K, 4.07 W/m K and 4.60 W/m K for the addition of 0%, 5% and 12% SWCNT. Additionally, Wu et al. [264] investigated the *K* of a continuous network of graphene foam (GF) filled with aligned graphene nanosheets (GNs). A relatively high in-plane *K* of 10.64 W/m K at the filler loading of 6.2 vol% was measured, which can be explained by the synergistic influence between 3D GF and graphene nanosheets.

#### 4.2.6. Improving Out-of-Plane Thermal Conductivity Value Using 3D Fillers

The self-heating phenomenon appearing in a PMC specimen during fatigue testing is often quantified by measuring the specimen’s surface temperature. If we assume the PMC specimen as a rectangular plate for simplicity, the total area of four lateral faces of a rectangular laminated PMC specimen (2W×t+2L×t, where L is length, W is width and t is thickness) is, in general, lower than the area of the two bases (2L×W) during standard fatigue testing. This means that using thermally conductive fillers through the thickness (out-of-plane) direction can act as a catalyst for transferring heat generation induced by the self-heating effect. According to the provided data for out-of-plane *K* values of PMCs (see Figure 7 in [243]), the out-of-plane *K* values of PMCs are relatively low. Numerous composites possess a *K* value of 4 W/m K or below, whereas a minority of them have a value higher than 10 W/m K when using a noncovalently functionalized boron nitride nanosheet (BNNS). Additionally, when a PMC has anisotropic and heterogeneous properties (e.g., transversely isotropic and orthotropic), the range of *K* values in the out-of-plane direction is significantly lower than that of other directions in general. Therefore, it is of primary significance to enhance the out-of-plane *K* of such composites.

**Figure 66:**
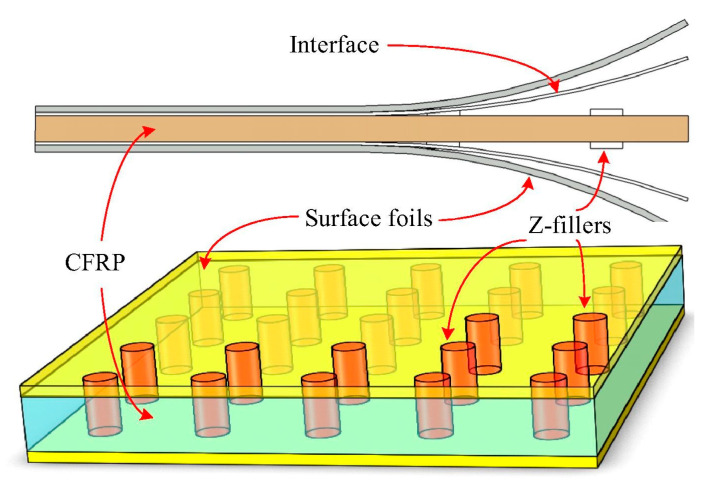
Schematic demonstration of z-filler CFRP laminated composites, adapted with permission from Ref. [265]. Copyright © 2016 Elsevier Ltd.

For the purpose of enhancing the *K* value in out-of-plane direction by constructing a 3D thermally conductive pathway, Yu et al. [265] prepared z-filler CFRP laminated composites filled with spherical copper particles and coated with aluminum foil in order to investigate the influence of z-filler volume fraction on the out-of-plane *K* value. Figure 66 reveals a schematic representation of the developed z-filler CFRP laminated composites shown by them. With the decrease in the distance between z-copper fillers (i.e., higher volume fraction), the measured *K* value increased. They reported a maximum increase of roughly 12 times in the out-of-plane *K* of z-filler laminated composites (7.6 W/m K) for a 2 mm distance between every two z-fillers, compared with unmodified CFRP composites (0.6 W/m K).

**Figure 67:**
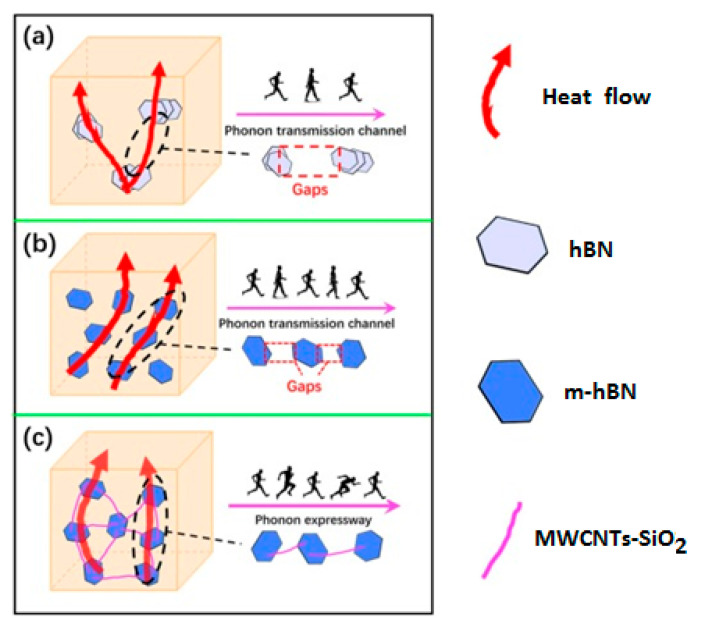
The schematic representation of thermally conductive pathways of (**a**) hBN/PVDF, (**b**) m-hBN/PVDF and (**c**) m-hBN/MWCNT-SiO2/PVDF composites, adapted with permission from Ref. [230]. Copyright © 2020 Elsevier Ltd.

The implementation of 3D fillers and the construction of interconnected three-dimensional (3D) graphene networks can be also a solution. As opposed to PMCs reinforced with dispersed graphene nanoplates (GNPs), implementing 3D GNPs in PMC structures can result in comparatively higher *K* values. This can be explained by adequate phonon transmission channels and a reduction in the thermal resistance in the continuously interconnected 3D GNPs [266]. Nevertheless, the high cost and unfavorable mechanical properties limit the application of 3D graphene-based polymer composites in many engineering fields involving thermoregulation applications [266]. Compared to the traditional polymer composites filled with randomly dispersed graphene nanosheets, constructing 3D GNs in polymer composites emerges as a more effective strategy to achieve high *K* values at relatively low filler loadings due to sufficient phonon transmission channels and reduced thermal resistance in the continuous interconnected 3D GNs. In another study, Hu et al. [230] constructed 3D hybrid networks in poly(vinylidene fluoride) composites via positively charged hexagonal boron nitride- and silica-coated carbon nanotubes (m-hBN/MWCNT-SiO_2_/PVDF). Three-dimensional networks could decrease the interfacial thermal resistance ($R_{k}$), leading to a higher level of phonon transmission (or decreasing phonon scattering at the filler–matrix interface) (see Figure 67). They measured a *K* value of 1.51 Wm·K for a 3D network of m-hBN/MWCNT-SiO2/PVDF with filler loading of 25 wt.%, and this *K* value was just below 6 times higher than that of pure PVDF.

#### 4.2.7. The Role of Interface Treatment, Coating and Core in Enhancing Out-of-Plane Thermal Conductivity

One of the other possible solutions to enhance the out-of-plane *K* value of the MC structures is to implement surface treatment (e.g., coating and films). Although films and coated layers are thermally conductive, their thicknesses may act as a driving factor in obtaining high *K* values; the thickness should be sufficient, neither very thick nor very thin, depending on the type of materials. A greater thickness may disadvantageously provoke a low in-plane *K* value. For this reason, Chen et al. [237] studied the influence of thickness on the in-plane *K* value of boron nitride nanosheet (BNNS)-poly vinylidene difluoride (PVDF) nanocomposite films. They reported a significant *K* reduction in BNNS- PVDF nanocomposite film, varying from 16.3 W/m K to 5 W/m K as the result of increasing thickness from 18 μm to 170 μm. Therefore, the thickness of a thermally conductive coating layer or film takes center stage in prompting phonon transmission for achieving high *K* values. This parameter is highly dependent on the material types, which should be taken into consideration before the manufacturing process of composite structures. On the other hand, controlling the fiber–matrix interface is of primary significance and is not an easy task due to the scattering of phonons at the interface. A sizable portion of phonon scattering is due to poor nonbonded van der Waals interactions in the interface of the polymer matrix and filler. The insufficient vibrations among atoms at the interface resulting from weak nonbonded interactions lead to a significant thermal resistance at the interphase region, and consequently a low *K* value [189,267]. To address this issue, a non-covalent filler surface modification (coating) can be implemented, which decreases the thermal resistance between fibers and the bulk polymer matrix [268]. The main approaches for preparing coating structures include silica deposition [269] and the self-polymerization of dopamine [270] and tannic acid [271] via bio-inspired polyphenols. The *K* value of such coated polymeric materials may vary and drop compared to that of uncoated composite, unless the coating layer thickness is below 10 nm [229]; i.e., the coated layer acts as a thermal resistance barrier if its thickness exceeds the mentioned value. In general, the interface between the filler and polymer and the interface between composite layers subjected to thermal fatigue play a key role in the service life of the composite structures, so a weak interface can provoke thermal fatigue delamination and structural degradation [272]. Nevertheless, there is still a lack of knowledge about how such surface treatments and the use of thermally conductive films can extend the fatigue life of PMC structures. As a recommendation, a comprehensive investigation of the effects of surface treatment on the overall fatigue performance of the PMC structures by taking into account the loading parameters (e.g., loading frequency, stress level and strain rate) is meaningful and necessary in future studies.

From another perspective, in addition to FRPs, composite sandwich panel structures have gained great interest, particularly in WTB, aerospace and marine industries, due to their superior specific flexural properties and cost-effectiveness [273,274]. Such structures may be subjected to cyclic loading and experience self-heating effects (e.g., WTB). Therefore, for such applications, the idea is to select an appropriate core with relatively high thermomechanical performance (i.e., high *K* value). Such structures involve laminated facesheets and cores. It has been suggested that the core can be appropriately selected to obtain thermally conductive sandwich structures. Typical cores with engineering applications can be primarily categorized into corrugated, truss, foam and honeycomb cores [275]. Among them, foam cores offer more uniform heat transfer distribution due to forming comparatively lower empty volumes in the core region (e.g., carbon foam) [276]. When it comes to finding or proposing an alternative solution for minimizing the catastrophic effect of the self-heating phenomenon, polymeric foams involving low-density polyethylene (LDPE) and polypropylene may not be good options due to exhibiting very low thermal conductivities and poor thermal instability under high temperature regimes; additionally, metallic foams including aluminum might be an ineffective solution despite possessing good thermal stability and *K*. The applications of metallic foams in polymeric sandwich structures have been limited due to the inherently weak bonding between the metallic core and the FRP facesheets and significant mismatch in their thermal expansion properties leading to facesheet/core delamination. Carbon foam, however, can be a prime candidate in terms of enhancing heat transfer through the thickness of lightweight sandwich panel structures [276,277,278]. Among available carbon foams, graphitic foam provides an outstanding *K* value with a low thermal expansion coefficient and density. For this reason, Sihn and Rice [279] conducted four-point bending fatigue tests for FRP facesheet/carbon foam core sandwich beams. In another study, Sihn et al. [276] measured the *K* of a sandwich structure composed of unidirectional graphite/epoxy facesheets and a carbon foam core. They reported the out-of-plane *K* values of 19.21 W/m K for the co-cured foam–facesheet sandwich with 5.99 mm thickness and 6.44 W/m K for the adhesively bonded sandwich specimens with 5.69 mm thickness. The main root of this comparatively high difference can be explained by interface thermal resistance ($R_{k}$), which was reported to be 0.0251 ${\mathrm{mm}}^{2}K/W$ for co-cured and 0.312 ${\mathrm{mm}}^{2}K/W$ for the co-cured and adhesively bonded sandwich specimens. Furthermore, Quintana and Mower [280] determined the *K* of sandwich panels. They reported *K* values of 60 W/m K and 10 W/m K, respectively, for an adhesively bonded Al facesheet–graphitic foam core, which was approximately 10 times greater than that of a CFRP facesheet–graphitic foam core sandwich panel.

On the other hand, honeycomb cores have received significant attention in advanced industries because they offer superior specific stiffness and strength [281], not because of their thermomechanical properties. Nonetheless, there are insufficient data to characterize whether honeycombs can effectively tackle the problematic issue of structural degradation induced by self-heating during fatigue loading. To correctly answer this, the *K* value of a typical honeycomb should be quantified first. For this reason, Bezazi et al. [282] developed an analytical in-plane model for determining the *K* of honeycombs, allowing us to understand whether the honeycomb acts as a heat sink or an insulator. It should be mentioned that, regarding minimizing the self-heating effect in sandwich panel structures, the through-thickness *K* may be more important and should be taken into consideration in further studies.

On the one hand, the amount of energy dissipation via conduction in honeycomb sandwich structures may be relatively lower compared to that in laminated FRP composites due to honeycomb sandwich structures possessing higher empty volume in the core region, which consequently leads to a low-density material/structure. A good illustration of this is a Nomex honeycomb with a density of 48 kg/m^3^ [283], which is more than 20 times lighter than engineering polymers such as epoxy at a constant volume. It should be mentioned that what primarily provokes thermal fatigue failure in a composite structure is a high level of stored internal energy. Assuming that the temperature is time-independent, one can conclude that the rate of stored internal energy ($\Delta \dot{u}$) in a sandwich panel with such a core remains constant and may be more than 20 times lower than the same figure for a composite without a core according to $\Delta \dot{u}=\rho c_{p}\frac{\partial T}{\partial t}$ (see Equation (13)). However, this assumption can be rational if and only if the steady-state heat transfer (i.e., stationary regime) occurs. Otherwise, the appearance of greater temperature accelerates the rate of internal energy storage and consequently maximizes the effect of self-heating. In general, it is difficult to exactly characterize the relation between the density of the core and the amount of internal energy because the density of the core may influence the heat capacity ($c_{p}$).

For this reason, Rajaneesh et al. [284] investigated the performance of CFRP/Nomex-honeycomb sandwich beams under four-point bending fatigue using the time–temperature superposition (TTS) principle by ignoring the influence of temperature on the honeycomb core behavior. However, it has not been proved yet whether the classical TTS principle can be used for high loading frequency (e.g., in the ultrasonic range).

One of the most challenging issues related to the honeycomb core sandwich panels may be developing accurate and nonlinear models for the purpose of predicting the residual life or structural degradation of such structures reliably. The primary reason behind this can be sought in the complexity of modeling the interfaces between facesheets. For this reason, Hu and Wang [285] developed a theoretical model in order to study the thermal fatigue delamination damage of auxetic and non-auxetic honeycomb layer/substrate structures under thermal cycling.

## 5. Conclusions

**Figure 68:**
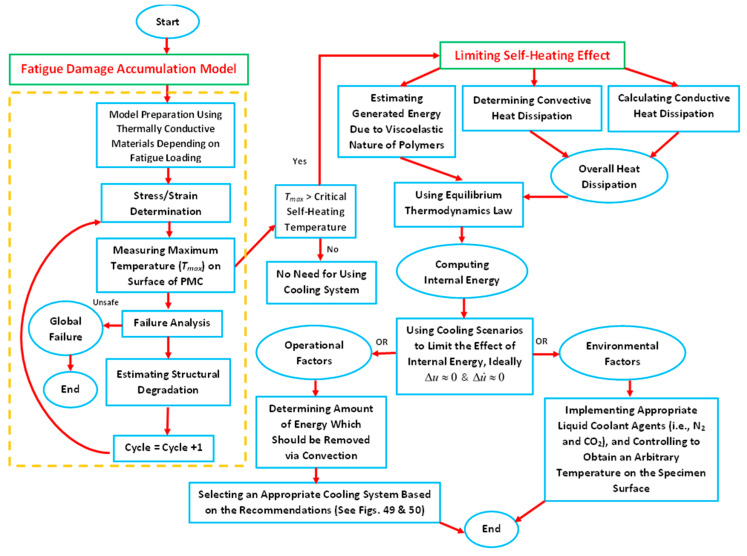
Schematic model for estimating structural degradation and preventing the appearance of self-heating phenomenon using cooling scenarios.

This paper systematically reviewed and discussed the appearance of the self-heating effect in polymers and PMCs under fatigue loading, including the theoretical and physical aspects. For this purpose, the fatigue fracture mechanisms in a PMC structure induced by the self-heating phenomenon were analyzed at three different scales (i.e., nano, micro, and macro). The practical solutions implemented by various teams for addressing the issue of a nonstationary self-heating regime induced during fatigue loading were then broadly reviewed. The viable solutions were classified based on optimal materials design and selection and cooling scenarios, considered the main core of the current paper. In the case of the former, selecting thermally conductive materials was proposed. Improving the *K* values of PMC structures was proposed. To increase the in-plane and out-of-plane *K* values of PMC structures, the influences of filler type (e.g., BN and carbon allotropes), the size (i.e., nano-, micro- or macroscale) and shape (e.g., 2D or 3D) of thermally conductive fillers, the filler volume fraction, the type of polymer in terms of the level of polymer crystallinity, the implementation of thermally conductive foams (e.g., carbon and graphite foams) and the use of coatings and interfacial films were analyzed, and these factors are taken into account during the manufacturing process of PMCs. From the other perspective, to limit the effect of internal energy, the influence of environmental factors (i.e., relative humidity and coolant agent temperature) and operational factors (i.e., mainly average convective heat transfer coefficient ($\bar{h}$)) was analyzed. In the case of operational factors, the influence of different cooling techniques, including air cooling, liquid cooling (e.g., water, liquid N_2_ and CO_2_ as the coolant agents), multiphase cooling (e.g., spray cooling) and the newly developed pulse–pause techniques, was investigated. In addition, the vital role of measuring heat flux via convection as well as quantifying $\bar{h}$ and improving the value of this parameter was broadly discussed as a guideline for future studies. Using cooling scenarios will decrease the amount of internal energy and consequently cool down the PMC specimen temperature. The diagram below represents the summary of the paper and provides a practical guideline for limiting the self-heating effect in PMC structures (see Figure 68). The diagram involves the characterization of (i) fatigue damage accumulations as well as searching for viable solutions to (ii) limit the self-heating effect and consequently limit the structural degradation in PMCs. The maximum temperature on the specimen surface is determined from the former using numerical simulations or experiments. If the maximum temperature is below the critical self-heating temperature, the specimen remains safe and without any requirement for implementing a cooling system or changing the ambient temperature. Otherwise, an appropriate cooling system and/or coolant agent should be selected according to the level of internal heat generation. The fatigue damage mechanisms and aspects connected with preventing structural degradation are briefly summarized in Section 5.1, Section 5.2 and Section 5.3.

### 5.1. Scale-Based Fatigue Damage Mechanisms

Due to the self-heating effect, the larger shrinkage of the polymer matrix compared to thermally conductive fibers may provoke chemical stress. However, to the best of the authors’ knowledge, the level of chemical shrinkage is sufficiently low below the critical self-heating temperature (i.e., before the beginning of phase III in the nonstationary regime—see Figure 24), which can be negligible. Furthermore, the self-heating phenomenon can cause thermal stress concentration in addition to the applied mechanical stress during fatigue. This can be explained by the CTE mismatch between fibers and the neighboring polymer matrix. The covalent bonds among polymer chains and the nonbonded electrostatic and van der Waals interactions begin to be weaker as the result of thermal stress. On the other hand, laminated PMC structures show different CTE values in different directions (e.g., for CTE, values are different in longitudinal and transverse directions of a transversely isotropic PMC structure such as CFRP). This difference can be explained by using layers with different staking sequences and the anisotropy behavior of layers. These factors can initiate and/or accelerate the degradation process of cyclically loaded PMC structures, which leads to quite complex fracture mechanisms and consequently the sudden fatigue failure in the structure. The main conclusions associated with the scale-based fatigue damage mechanisms with the presence of the self-heating effect are summarized as follows:

1. Polymer degradation due to the self-heating effect, which is associated with residual crosslinking, can initiate the crack nucleation process and propagate the damage.
2. To avoid structural degradation in PMCs, it is necessary to measure and quantify the size of cracks and delaminations in both the micro- and macroscale.
3. Delamination and interfacial debonding damage can be the critical forms of fatigue damage mechanisms. To characterize this in continuum mechanics, it is assumed that both fibers and polymer matrix are perfectly bonded. This means that there is a continuity in the displacement field, and consequently a continuity in the strain field, as opposed to the stress field wherein there may be discontinuity due to the dependency of stress on viscoelastic moduli. Therefore, measuring the strain field can be an indicator of damage caused by the self-heating effect in a PMC structure.

### 5.2. Cooling Scenarios and Techniques

As a practical guideline, before implementation of any cooling scenario, it is of primary significance to monitor whether the cyclically loaded PMC structure operates in a stationary or nonstationary regime. This can be understood by measuring the critical self-heating temperature. If. the specimen’s surface temperature is below the critical temperature, there is no need to implement any cooling technique. Otherwise, implementing an appropriate cooling technique is vital to provide uniform temperature distribution as well as limit the PMC specimen surface temperature growth induced by the self-heating effect. For this reason, two possible scenarios were discussed, involving environmental factors (i.e., relative humidity and ambient temperature of coolant agent) and operational parameters (i.e., Nusselt number, thermal conductivity of fluid around the specimen and characteristic length of the specimen). The operational parameters were explained by the average convective heat transfer coefficient ($\bar{h}$). The main points related to cooling techniques and scenarios are as follows:

1. Conducting fatigue testing exposed to an environment with high moisture content can degrade the mechanical properties of a PMC specimen unless the moisture does not penetrate through the specimen’s thickness. This means that the time of fatigue testing should be sufficiently low, which may be achieved in ultrasonic frequency.
2. Reduction in ambient temperature using liquid coolant agents such as water, liquid nitrogen and liquid carbon dioxide is a viable solution. An appropriate coolant system can be selected by making an optimization based on high thermal capacity, low viscosity, anticorrosion properties, nontoxicity and affordable price.
3. The operational factors, which have a direct influence on the fatigue life of PMCs, are directly connected with $\bar{h}$. A greater $\bar{h}$ offers a larger amount of heat removal through convection. Therefore, fatigue life extension can be obtained by enhancing this parameter.
4. The discussed cooling techniques, including air cooling, liquid cooling, passive cooling, pulse–pause air cooling, ring-type Peltier cooling and spray cooling, can be implemented for removing the heat induced by the self-heating effect. This will consequently extend the fatigue life of a typical PMC structure.
5. Air cooling, as the simplest cooling method, is applicable for removing relatively low heat flux (i.e., 5 kW or below according to recommendations, see Figure 49 and Figure 50 for more details). However, for removing higher amounts of heat up to 80 kW, liquid-based cooling techniques are the prime candidates. For amounts higher than 80 kW, phase change techniques (e.g., spray cooling using the latent heat of vaporization of water) can be implemented.
6. The pulse–pause air cooling technique and the use of cryogenic nitrogen and pulse–pause simultaneously as a cooling technique have only been implemented for the VHCF regime in an ultrasonic frequency range.
7. Quantifying the heat flux during fatigue testing leads to a reliable classification and standardization according to the type of fatigue loading, the type of materials used in the PMC structure and the specimen geometry.
8. The temperature distributions might not be uniform in in-plane directions and through the thickness, which, in connection with the heterogeneity of PMCs, results in quite complex fracture mechanisms.

### 5.3. Optimal Material Design and Selection

In the real life, a typical PMC structure may undergo a low number and/or very high number of cycles by experiencing different mechanical loading conditions, as well as various hygrothermal effects (i.e., moisture content and ambient temperature). Such a PMC structure should be designed and fabricated by considering all the below-mentioned material aspects, which are directly connected with improving thermal conductivity. It is then crucial to carry out comprehensive studies for a wide range of fatigue tests (i.e., LCF, HCF and VHCF regimes) to quantify the effect of material aspects on the *K* value and limit the structural degradation. The primary conclusions relevant to material aspects are as follows:

1. Implementing a thermally conductive filler with a sufficient volume fraction increases the *K* value of PMCs. Otherwise, it may be difficult to obtain relatively high *K* values because of phonon scattering accelerated by defects and imperfections involving chain dangling ends, entanglement and irregular orientation. Moreover, the addition of such a filler should be appropriately balanced from the mechanical point of view.
2. Using polymers with a high level of chain crystallinity is recommended since it can enhance the *K* values of polymers.
3. Thermally conductive carbon allotropes (e.g., carbon fiber, CNT, GN), ceramics (e.g., BN) and metals (Cu, Ag, Au) can be the prime candidates for improving the thermal conductivity of a typical pure polymer matrix.
4. Although implementing 3D fillers offers greater *K* values, using 2D fillers with greater aspect ratios (i.e., length to diameter for cylindrical fillers and the ratio of length to thickness for platelet fillers) may also offer quite good *K* values. In addition, 2D fillers can be easily implemented during the fabrication process of composites. Therefore, using 2D fillers with a high aspect ratio is preferable.
5. Implementing thermally conductive nanoparticles (e.g., BN, CNT and GN) is preferable to implementing micro- or macrofillers because thermally conductive nanoparticles have a significantly larger surface area to volume ratio.
6. Since the CTE of a typical polymer is generally higher than that of thermally conductive fillers, the residual thermal stresses during fatigue loading may appear at the interface/interphase region between the reinforcement and polymer, accelerating the molecular separation and lowering nonbonded van der Waals forces at the interphase zone between the filler surface and polymer chains. This will cause interfacial debonding and consequently high interfacial thermal resistance ($R_{k}$) between fillers and polymer chains, leading to relatively low *K* values. Therefore, it is promising to use fillers with low CTE values (e.g., carbon allotropes that have almost zero or even negative CTE values).
7. Using carbon/graphite foam can increase the *K* value, consequently limiting the self-heating effect.
8. Using films and surface coating has been proposed as a technique to achieve strong phonon transmission, consequently improving the *K* value.
9. Since thermal conductivity is temperature-dependent, it is noteworthy to measure and quantify the influence of self-heating temperature induced during fatigue loading on this parameter for an investigated PMC. This will help to evaluate the amount of heat flux via conduction in a more precise way.

## 6. Gap Analysis and Future Directions

As discussed in the previous sections, various studies have been conducted for characterizing the fatigue damage accumulation induced by self-heating at different scales during fatigue loading. To limit the self-heating effect, some cooling scenarios and thermally conductive materials were implemented by researchers, which were broadly discussed. Nevertheless, the challenging issues connected with understanding fatigue damage mechanisms, cooling techniques and material selections, which should be taken into consideration in future studies, are as follows:

1. It is worth conducting further studies to quantify the residual crosslinking in different engineering PMCs at various fatigue loading levels with the presence of the self-heating effect.
2. There are no studies in the open literature about how the self-heating phenomenon can be quantitively influenced by changing the level of chain crystallinity of the polymer matrix. Therefore, the effect of polymer chain crystallinity alongside the self-heating phenomenon on the overall fatigue performance of PMC structures should be studied under different loading conditions in the future.
3. Although using image-based techniques such as X-ray CT scanning offers the opportunity to measure the volume of microcracks and delaminations, there is still a lack of knowledge and understanding to quantify the size of delaminations and cracks caused by the self-heating effect in PMC structures.
4. It is of key interest to experimentally and numerically characterize the strain distribution at the fiber–matrix interface and measure the strain between every two layers as an indicator of fatigue damage accumulation.
5. Although a PMC structure is subjected to a complex type of loading during its real lifetime, the vast majority of studies have focused on merely one-directional loading to characterize the structural degradation mechanism of PMC coupons. Therefore, it is noteworthy to investigate the structural degradation mechanisms under more complex fatigue loading (e.g., random and multiaxial loading) considering numerous practical problems, where self-heating may appear in such loading conditions.
6. To limit the fatigue damage propagation in PMC structures, it is useful to conduct further studies to investigate the effect of different stacking sequences, as well as various types of nanoparticles in polymers as modified FRP composites.
7. To limit the self-heating effect, it is of primary significance to conduct a comprehensive investigation of different liquids for the purpose of selecting an appropriate coolant system by making an optimization based on high thermal capacity, low viscosity, anticorrosion properties, nontoxicity and affordable price.
8. Although the mentioned techniques indeed operate based on increasing $\bar{h}$, there are no data about this parameter from the previous studies wherein cooling techniques had been implemented to minimize the influence of the self-heating effect. Thus, it would be a practical guideline if this parameter is also reported when using a cooling method for cyclically loaded PMC structures in further studies.
9. There are insufficient data to understand how liquid-based cooling techniques can effectively address the heat generation caused by the self-heating effect in PMCs.
10. In the pulse–pause cooling technique, the investigation of the optimal pulse–pause ratio at a specific applied stress/displacement loading is still ongoing. In addition, no results have been reported about the capability of pulse–pause technique in terms of removing the amount of heat energy. Another challenging issue associated with this technique is the characterization of whether it is possible to use this technique for a relatively low range of frequency (e.g., 1000 Hz or below). Moreover, since the common fatigue tests are mainly conducted at a continuous strain rate, using the pulse–pause technique leads to discontinuity of applied loading. It should be investigated whether this discontinuity has an influence on fatigue performance. Therefore, it is noteworthy to conduct research in this area to comprehensively quantify and generalize the effect of pulse–pause ratio and find the optimal pulse–pause ratio as a function of material configuration, loading conditions and geometry of the PMC specimens.
11. It is recommended to quantify and report the heat flux during fatigue testing to justify the reason behind selecting a specific cooling technique in future studies.
12. It is of high importance to compare the fatigue fracture mechanisms of PMCs using cooling techniques versus natural air convection as this will allow the effectiveness of such cooling techniques to be understood.
13. Although implementing thermally conductive fillers is preferable, the thermomechanical performance of such materials should be characterized in further studies.
14. Quantification of the influence of thermally conductive fillers on limiting the self-heating effect and consequently extending the fatigue life of PMC structures in the presence of the self-heating effect is of key interest.
15. It is useful to conduct various comparative studies to characterize the influence of 2D and 3D fillers under different levels of fatigue loading on limiting/accelerating the self-heating effect in PMCs.
16. The effect of scale-based fillers on the fatigue performance of PMC structures with the presence of the self-heating effect can be taken into consideration for the future studies.
17. Quantifying the admissible thermally conductive film thickness is of key importance, resulting in limiting the self-heating effect
18. Since PMCs have anisotropic properties, it is useful to study how the self-heating can be limited in the in-plane and the out-of-plane directions by measuring *K* values.
19. Whether the use of cores accelerates the rate of thermal fatigue failure is still an open question. Additionally, it is unknown how the maximum temperature values on the specimen surfaces under fatigue loading are influenced by variations in core thickness. As a recommendation, it would be useful to conduct comprehensive studies to quantify the fatigue behavior of sandwich structures with a wide range of core types with different thicknesses in the presence of the self-heating effect by controlling loading conditions (e.g., mainly loading frequency, stress levels, stress ratios for both LCF and VHCF regimes) and compare the obtained data with such data for composites without cores.

## Figures and Tables

**Table 1:** Thermal conductivity (*K*) of commonly used polymers.

Polymer Matrix	*K* (W/m K)	Refs.
Elium	0.18	[184]
Epoxy resin (EP)	0.148, 0.22	[185,186]
Polyimide (PI)	0.2, 0.27	[187,189]
Polycarbonate (PC)	0.19	[188]
Polyetheretherketone (PEEK)	0.25	[190]
Polyphenylene sulfide (PPS)	0.22–0.25	[190,194]
Polysulfone (PSU)	0.28	[190]
Polytetrafluoroethylene (PTFE)	0.25	[190]
Nylon-6 (PA6)	0.29	[190]
Nylon-6.6 (PA66)	0.23	[190]
Poly (ethylene terephthalate) (PET)	0.24	[190]
Polymethylmethacrylate (PMMA)	0.18	[190]
Polypropylene (PP)	0.17–0.22	[190]
Low-density polyethylene (LDPE)	0.32–0.40	[190]
High-density polyethylene (HDPE)	0.38–0.51	[190]
Polyester	0.091	[191]
Polystyrene (PS)	0.14	[192]

**Table 2:** Thermal conductivity of commonly used fibers.

Filler	*K* (W/m K)	Ref.
Graphene (GNP)	2000–6000	[194,195]
Carbon nanotube (CNT)	2000–6000	[194,196,197]
Diamond	2000	[196,198]
Pitch-based carbon fiber	400–1100 (along the axis)	[194,196]
PAN-based carbon fiber	8–70 (along the axis)	[196]
Carbon black	6–174	[196]
Graphite	100–800	[196,199]
Boron nitride (BN)	250–1300	[196,200]
Silver (Ag)	427	[198]
Copper (Cu)	398	[198]
Gold (Au)	315	[198]
Aluminum (Al)	247	[198]
Tungsten (W)	155	[198]
Nickel	158	[196]
Zink (Zn)	115	[189]
Steel	50	[189]
Silicon carbide (SiC)	270	[198]
Glass	1–1.03	[191,201]
Kevlar (aramid)	0.04	[191]
Basalt	0.031–0.038	[202]

**Table 3:** Typical models for determining *K* of filler-embedded PMCs.

Authors	Filler Shape	Model	Remarks
Hasselman et al. [248]	Sphere	$\begin{array}{l} \frac{K_{eff}}{K_{m}}=\frac{K_{f}(1+2\alpha )+2K_{m}+2\phi [K_{f}(1-\alpha )-K_{m}]}{K_{f}(1+2\alpha )+2K_{m}-\phi [K_{f}(1-\alpha )-K_{m}]}\\ where\;\;\;\;\alpha =2R_{k}K_{m}/d \end{array}$	Appropriate for moderate volume fraction, *ϕ* < 0.4
Maxwell [249]	Sphere	$\frac{K_{eff}}{K_{m}}=\frac{K_{f}+2K_{m}+2f[K_{f}-K_{m}]}{K_{f}+2K_{m}-f[K_{f}-K_{m}]}$	Assuming perfect thermal contacts in polymer–filler interface (i.e., *R_k_* = *α* = 0), and *ϕ* < 0.4
Eucken [250]	Sphere	$\frac{K_{eff}}{K_{m}}=\frac{1-\phi }{1+\phi /2}$	Assuming nanoparticles act like nanopores (i.e., *α*→∞), and *ϕ* < 0.4
Hasselman et al. [248]	Sphere	$\frac{K_{eff}}{K_{m}}=\frac{1+2\alpha +2\phi (1-\alpha )}{1+2\alpha -\phi (1-\alpha )}$	Suitable when *K_f_* ≫ *K_m_*, e.g., using CNTs and GNPs
Ma and Na [251]	Sphere	$(1-\phi )\frac{K_{m}-K_{eff}}{K_{m}+2K_{eff}}+\phi \frac{K_{f}-K_{eff}(1+\alpha K_{f}/K_{m})}{K_{f}+2K_{eff}(1+\alpha K_{f}/K_{m})}=0$	Appropriate for high volume fraction, and *ϕ* > 0.4
Every et al. [252]	Sphere	${\left(1-\phi \right)}^{3}={\left(\frac{K_{m}}{K_{eff}}\right)}^{\left(1+2\alpha )/(1-\alpha \right)}{\left[\frac{K_{eff}-K_{f}(1-\alpha )}{K_{m}-K_{f}(1-\alpha )}\right]}^{3/(1-\alpha )}$	Assuming *α* = 0 or *K_f_* ≫ *K_m_*, and *ϕ* > 0.4
Bryning et al. [253]	Cylinder	$\begin{array}{l} \frac{K_{eff}}{K_{m}}=\frac{\left(3+\phi \left(\beta_{⊥}+\beta_{∥}\right)\right)}{\left(3-\phi \beta_{⊥}\right)}\\ where\\ \beta_{⊥}=\frac{2\left(d\left(K_{CNT}-K_{m}\right)-2R_{k}K_{CNT}K_{m}\right)}{d\left(K_{CNT}+K_{m}\right)+2R_{k}K_{CNT}K_{m}}\\ \beta_{∥}=\frac{L\left(K_{CNT}-K_{m}\right)-2R_{k}K_{CNT}K_{m}}{LK_{m}+2R_{k}K_{CNT}K_{m}} \end{array}$	Appropriate for CNT-embedded composites, e.g., for N-N-Dimethylformamide (DMF)-processed composites $R_{k}=(2.4\pm 1.3)\times 10^{-9}\;m^{2}K/W$, and for surfactant-processed composites $R_{k}=(2.6\pm 0.9)\times 10^{-8}\;m^{2}K/W$
Hasselman et al. [248]	Cylinder	$\frac{K_{eff}}{K_{m}}=\frac{\left[\phi \left(K_{f}/K_{m}-1-2K_{f}/\left(dR_{k}\right)\right)+\left(1+K_{f}/K_{m}+2K_{f}/\left(dR_{k}\right)\right)\right]}{\left[\phi \left(1-K_{f}/K_{m}+2K_{f}/\left(dR_{k}\right)\right)+\left(1+K_{f}/K_{m}+2K_{f}/\left(dR_{k}\right)\right)\right]}$	Developed for a continuous matrix phase with dilute concentrations of dispersions with cylindrical geometry
Hasselman et al. [248]	Flat plate	$K_{eff}=\frac{K_{f}}{\phi \left(1-K_{f}/K_{m}+2K_{f}/\left(tR_{k}\right)\right)+K_{f}/K_{m}}$	Used for flat plate dispersions oriented perpendicular to the heat flow with thickness of t
Nielsen [254]	Various particle shapes	$\begin{array}{l} K_{eff}=\frac{1+AB\varphi }{1-B\psi \varphi }\;\\ \mathrm{where}\\ B=\left(\frac{K_{f}/K_{m}-1}{K_{f}/K_{m}+A}\right);\;\;\psi =1+\left(\frac{1-\varphi_{m}}{\varphi_{m}{}^{2}}\right)\varphi \end{array}$	Appropriate for moderate volume fraction, *ϕ* < 0.4. *A* is the shape coefficient for the filler particles; *φ_m_* is the maximum filler volume fraction

## Data Availability

Not applicable.
